# Photoactivatable Fluorophore for Stimulated Emission Depletion (STED) Microscopy and Bioconjugation Technique for Hydrophobic Labels

**DOI:** 10.1002/chem.202004645

**Published:** 2020-11-26

**Authors:** Michael Weber, Taukeer A. Khan, Lukas J. Patalag, Mariano Bossi, Marcel Leutenegger, Vladimir N. Belov, Stefan W. Hell

**Affiliations:** ^1^ Department of NanoBiophotonics Max Planck Institute for Biophysical Chemistry Am Faßberg 11 37077 Göttingen Germany; ^2^ Department of Optical Nanoscopy Max Planck Institute for Medical Research Jahnstraße 29 69120 Heidelberg Germany; ^3^ present address: Stratingh Institute for Chemistry Zernike Institute for Advanced Materials University of Groningen Nijenborgh 4 9747 AG Groningen The Netherlands

**Keywords:** fluorescence, optical superresolution, photoactivation, protein labelling, stimulated emission depletion (STED)

## Abstract

The use of photoactivatable dyes in STED microscopy has so far been limited by two‐photon activation through the STED beam and by the fact that photoactivatable dyes are poorly solvable in water. Herein, we report ONB‐2SiR, a fluorophore that can be both photoactivated in the UV and specifically de‐excited by STED at 775 nm. Likewise, we introduce a conjugation and purification protocol to effectively label primary and secondary antibodies with moderately water‐soluble dyes. Greatly reducing dye aggregation, our technique provides a defined and tunable degree of labeling, and improves the imaging performance of dye conjugates in general.

## Graphical abstract

**ONB‐2SIR**, a photoactivatable dye resistant against two‐photon uncaging by 775 nm STED light is synthesized and used to perform STED microscopy before and after photoactivation without undesired activation by the STED light. The effects of the low water‐solubility of ONB‐2SIR on antibody conjugation was overcome by glycan labelling with defined degree of labelling (DOL) and a phase separation purification method.

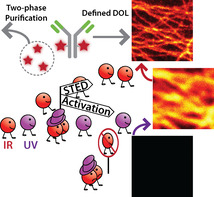

## Introduction

The conversion of a non‐fluorescent compound into a fluorescent form and back is at the heart of fluorescence microscopy (nanoscopy) with diffraction‐unlimited spatial resolution. While the earliest methods (STED,^[1]^ GSD,^[2]^ (S)SIM,^[3]^ RESOLFT^[4]^) utilized structured light patterns to modulate the emission capability of fluorophores in order to make adjacent details distinguishable, the methods called STORM,^[5]^ PALM,^[6]^ GSDIM^[7]^ or dSTORM^[8]^ made nearby features distinguishable by individually switching the marker molecules to an emissive state for a brief period of detection and localization. The combined use of single molecule switching (activation and de‐activation) for separation and patterned illumination for localization (MINFLUX^[9]^) allows achieving even molecule‐scale resolution in lens‐based fluorescence microscopy. In any case, all superresolution microscopy approaches require fluorophores with properties tailored to the particular imaging technique.

**Scheme 1:**
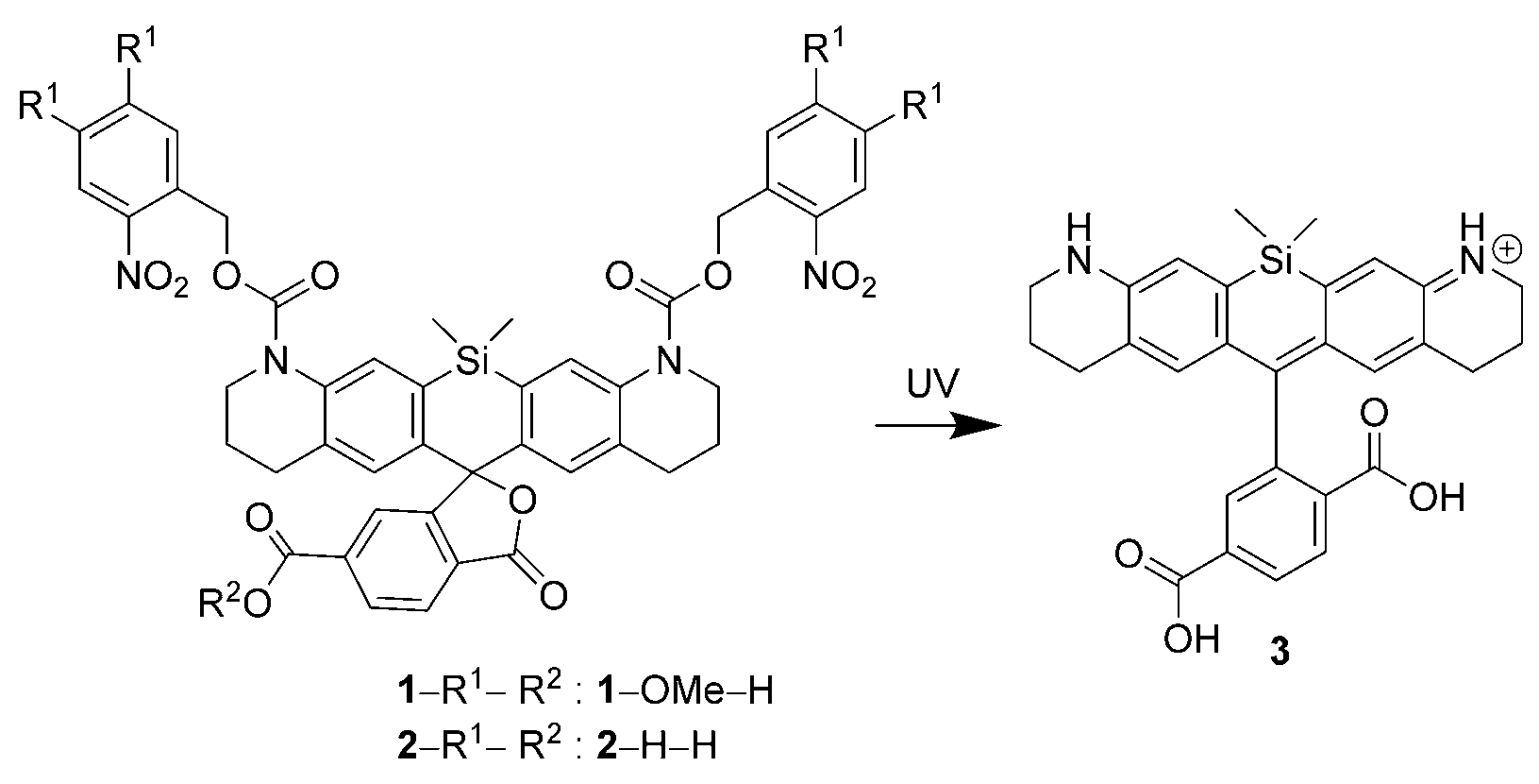
Photoactivation of SiR by photocleavage of *ortho*‐nitrobenzyl carbamates.

Multiple pathways can be taken in order to convert non‐fluorescent molecules to fluorophores and vice versa. Besides reversibly photo‐switchable fluorescent dyes, which rarely provide sufficient contrast between the non‐fluorescent and fluorescent states, irreversibly ‘photoactivatable’ dyes are the most common and abundant. Photoactivation (i.e. conversion into a state in which the dye can be excited to fluorescence) of organic dyes is often based on a Wolff rearrangement^[10]^ or Norrish Type II reactions.^[11]^ The most important features of a good photoactivatable fluorophore are a high photoconversion degree, bright fluorescence, good water‐solubility, and insensitivity to light other than the specific activation wavelength. Among the most useful types of fluorophores suitable for superresolution microscopy are rhodamines, carbopyronines, silicon rhodamines and related xanthene structures with extended conjugated systems. The switching capacity for these structures is often achieved by transfer to a poorly conjugated system; in particular, cyclic and uncharged derivatives (esters, amides) formed upon an intramolecular nucleophilic attack. While such transformations significantly decrease water‐solubility, thus making bioconjugation quite challenging, the hydrophilic properties can be restored by the use of polar groups and linkers.^[12]^ Such „masked“ fluorophores have been extensively used for single‐molecule localization microscopy.^[13]^ Nonetheless, the combination of a photoactivation and STED imaging offers unique possibilities in superresolution, such as counting the number of fluorophores participating in STED imaging.^[14]^ The spatial control of the active fluorophores can be used to protect the fluorophores from bleaching by the STED laser and to increase the spatial resolution. On the other hand, realizing this combination brings about additional complexity, since high resolution STED microscopy entails relatively high photon fluxes eliciting uncontrolled activation by two‐photon absorption from the typically pulsed STED laser beam.^[13b]^ Silicon rhodamines (SiR) are widely used in fluorescence microscopy and STED nanoscopy, especially for live‐cell imaging. A photoactivatable SiR with photocleavable 4,5‐dimethoxy‐2‐nitrobenzyl (*DiMeO‐ONB*) groups was reported and applied in photoactivation localization microscopy (PALM) of actin.^[13d]^ However, this caging group is cleaved by the STED light, which precludes its use in STED nanoscopy. We found that the activation by the STED beam may be as high as 10 % after one scan, thus making any prior sequential (time‐lapse) measurements of other fluorophores impossible due to progressively increasing activation of *DiMeO‐ONB*. We introduce a photoactivatable SiR (ONB‐2SiR, see Scheme 1), which has been designed to overcome this drawback. In STED nanoscopy, ONB‐2SiR produces images with excellent quality and without two‐photon activation. Without the use of complex solubilizing modifications of the structure, we introduce a general conjugation and purification technique, which overcomes the limitations associated with low water‐solubility and dye aggregation that would lead to low degrees of labelling (DOL) and unspecific antibody staining. We expect this protocol to be applicable and efficient for bioconjugation of any hydrophobic dye or molecule, far beyond caged xanthene fluorophores.

## Results and Discussion

**Scheme 2:**
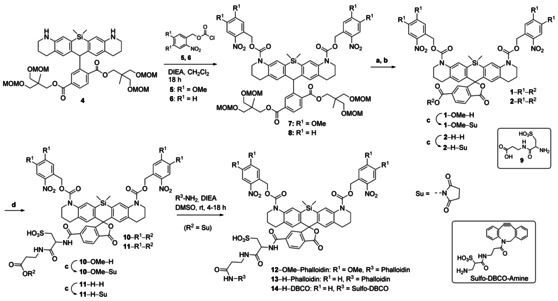
Synthesis of leuco‐dyes, photoactivatable dyes and their conjugates decorated with a hydrophilizer linker, phalloidin and strained alkyne. Reagents and conditions: (a) LiOH, THF, H_2_O, 55 °C, 48 h; (b) DDQ, CH_2_Cl_2_, H_2_O, rt, 18 h; (c) TSTU, DIEA, DMSO, rt, 1–18 h; (d) **9**, DIEA, DMSO, rt, 1–18 h.

**Figure 1:**
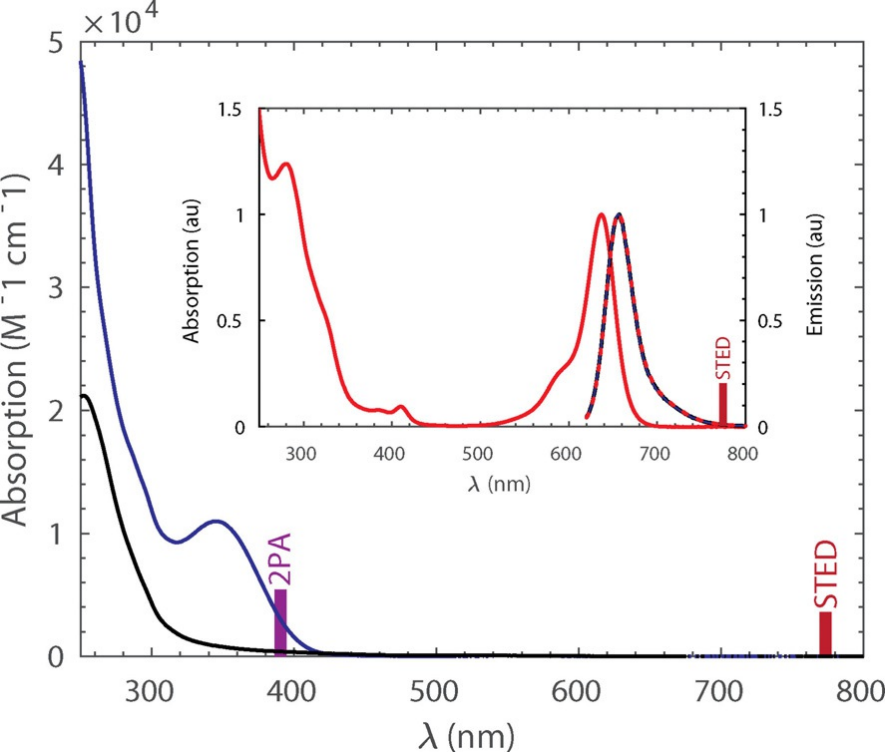
Absorption of compounds **1**‐OMe‐H (blue) and **2**‐H‐H (black) in a 1:1 mixture of acetonitrile and aq. phosphate buffer (100 mm, pH 7). The inset shows the normalized absorption and fluorescence of **3** (red) together with the fluorescence of **1**‐OMe‐H and **2**‐H‐H after UV activation.

A key process in STED microscopy is the rapid transfer of the excited fluorophores from the first excited singlet state S_1_ back to a high vibrational level of the ground state S_0_ by stimulated emission. To avoid the undesired excitation by the stimulating beam, its wavelength is typically chosen at the red edge of the fluorophore emission band. The most popular laser wavelengths for STED are 590 nm, 660 nm, and particularly 775 nm which is readily available and widely used due to its low photo‐toxicity and high tissue penetration. To achieve a high resolution, the cross section of stimulated emission, which is proportional to the emission probability at that wavelength, should be maximized. As a compromise between STED efficiency and spurious excitation by the STED beam, fluorophores emitting between 610–680 nm (emission maximum) are depleted at 775 nm. To date, the best performing dyes in STED microscopy are mostly xanthenes featuring high photostability, large fluorescence quantum yields and extinction coefficients of about 10^5^ cm^−1^ m ^−1^. An emission maximum at about 660 nm can be achieved with rhodamines, carbopyronines, SiRs and germano‐rhodamines. We chose a photoactivatable SiR^[13d]^ as a scaffold because of the excellent STED performance of SiRs with a 775 nm STED beam^[15]^ and the compatibility of these dyes with live‐cell experiments.[^15^, ^16^] The *ortho*‐nitrobenzyl photocaging groups, which can be chemically introduced into the SiR structure are shown in Scheme 1. Importantly, this caging strategy leads to a fluorophore without an electric charge and with poor solubility in water. In order to compensate for these undesirable properties, we used a „universal solubilizer“^[12]^ (see Scheme 2), as had been previously applied on a diazoketone caged rhodamine to improve its poor solubility. The relatively high STED beam intensities applied in STED nanoscopy can lead to adverse two‐photon induced photoreactions in the UV region.^[13b]^ The *DiMeO‐ONB* photocleavable group has substantial absorption around 400 nm, a region where two‐photon interactions with the 775 nm STED beam can take place (Figure 1). To prevent or reduce this effect, we decided to shift the absorption of the photocleavable group to shorter wavelengths by using the *ortho*‐nitrobenzyl (*ONB*) group without additional substituents. However, this choice may lead to complications because the light transmission of the optical components of the microscope is low in the UV region, and aberrations are difficult to compensate at the wavelength of 350–370 nm required for the one‐photon activation (1PA) of *oNB*, compared to the 405 nm wavelength often used for cleaving *DiMeO‐ONB*. Nevertheless, many microscopes are equipped with an excitation light source <400 nm (e.g. for DAPI imaging), which turned out to be sufficient for uncaging of *ONB* through a 1PA process.

In Scheme 2, we show the synthesis of caged SiR bearing the 6‐carboxyl group for bioconjugation. The complete synthetic procedures are given in the Supporting Information. Compound **4** reacted with commercially available chloroformate (**5**) and freshly prepared *o*‐nitrobenzyl chloroformate^[17]^ (**6**) to give caged *leuco*‐SiR **7** and **8**. Saponification of esters **7** and **8** using aq. LiOH in THF yielded the desired dicarboxylic acids with 35 % and 37 % yield, respectively. These intermediates were oxidized with DDQ to obtain the target caged SiR **1**‐OMe‐H and **2**‐H‐H in 90 % yield Scheme 2). The free carboxyl group at C‐6 of the pendant phenyl ring was used as a bioconjugation handle. To overcome the poor water‐solubility of the caged dye, we attached a short sulfonate linker **9** (the „universal solublizer“^[12]^) to the dye before bioconjugation. Compounds **1**‐OMe‐H and **2**‐H‐H were converted to *N*‐hydroxysuccinimidyl esters and reacted with hydrophilizer **9** to give the more water‐soluble compounds **10**‐OMe‐H and **11**‐H‐H with 57 % and 96 % yields, respectively (Scheme 2). The hydrophilized caged dyes were converted to NHS esters **10**‐OMe‐Su and **11**‐H‐Su, and these active esters were then used to produce conjugates with antibodies and ligands. The NHS esters **10**‐OMe‐Su and **11**‐H‐Su reacted with aminophalloidin in DMSO to provide the phalloidin conjugates **12**‐OMe‐Phalloidin and **13**‐H‐Phalloidin. Similarly, the dibenzocyclooctyne containing dye **14**‐H‐DBCO was prepared by reacting the sulfo‐DBCO‐amine with NHS ester **11**‐H‐Su. The compound **14**‐H‐DBCO was used to obtain antibody conjugates of ONB‐2SiR by means of click chemistry.

**Figure 2:**
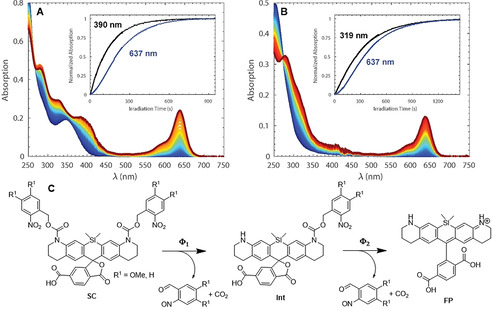
Uncaging experiments with compounds **1**‐OMe‐H (**A**) and **2**‐H‐H (**B**) in a 1:1 mixture of acetonitrile and aq. phosphate buffer (100 mm, pH 7). The time is color‐coded from blue to red. Solutions were stirred and irradiated with 365 nm light, and the absorption was monitored at constant intervals (at 20 °C). The insets show the temporal evolutions at 637 nm (blue symbols), where only the final product absorbs, and at a wavelength where the intermediate and the final product absorb (A: 316 nm or B: 390 nm, black symbols), along with a global fit (black/blue lines) fitting a scheme with two consecutive reactions. (**C**) Proposed uncaging process, consisting of two consecutive photochemical reactions.

Since the photo‐physical properties are only weakly influenced by additional linkers,^[12]^ the spectroscopic analysis was performed on the compounds without a hydrophilizer linker. The absorption spectra of compounds **1**‐OMe‐H, **2**‐H‐H and the reference compound **3** (uncaged dye) are given in Figure 1. Due to the limited solubility of the caged compounds, studies were performed in mixtures of buffered aqueous solutions (phosphate 100 mm, pH 7) and acetonitrile (1:1). The replacement of the *DiMeO‐ONB* groups with *ONB* resulted in a hypsochromic shift of the absorption maximum of almost 100 nm. At 355 nm, the ratio of the absorption coefficients (compound **1**‐OMe‐H/ compound **2**‐H‐H) is 12.6. Nevertheless, compound **2**‐H‐H has a residual absorption „foot“ in the range 350–405 nm, which is sufficient for activation with a commercially available laser or wide‐field illumination source. Both compounds show no detectable absorption and fluorescence in the red before activation. The activation of both compounds was performed in bulk experiments with a 365 nm LED source. To evaluate the quantum yields of the uncaging reactions, 1–2 μm solutions of caged compounds were irradiated in a custom setup with UV light until the starting material was consumed (Figure 2 and **SI3**–**4**). The growth of the absorption maximum of the photoproduct **3** (insets in Figure 2) shows a bi‐exponential behavior suggesting a consecutive two‐step reaction (Figure 2 C). The temporal evolution at the wavelength where all the products (including the byproducts produced by the caging groups) are expected to absorb (i.e. 316 nm or 390 nm), shows a mono‐exponential growth in agreement with this hypothesis.

To confirm this reaction sequence, LC‐MS experiments (Figures S1, S2) were performed in the course of irradiation (at the same irradiation conditions as shown in Figure 2 A, **B**). The presence of the mono‐caged fluorophores as intermediates was confirmed by MS analysis (Figures S1, S2). In addition, we observed these intermediates predominantly in the closed (lactone) forms, even under acidic conditions used in HPLC runs (0.1 % TFA in aqueous MeCN). Thus, we assume that these compounds are also in the closed form at pH 7, and the only products absorbing at 640 nm are the final, completely uncaged substances.

Remarkably, a clean photoreaction is observed throughout the whole irradiation period, with three compounds in each case (**1**‐OMe‐H and **2**‐H‐H) present in the proposed sequence (the nitroso benzaldehydes were not detected). Only traces of byproducts absorbing at 250–700 nm were observed. The emission quantum yield and fluorescence lifetime of the fluorescent product (in the reaction mixtures at the end of the photolysis) were identical in both cases to the reference compound **3** (Figures S**3**), excluding the presence of any observable fluorescent byproducts.

By integrating the areas under the HPLC peaks, the concentration changes of the three reactants were calculated. A global fit to consecutive reaction schemes yielded the values for $\left(\epsilon_{SC,Int}^{\lambda_{irr}}\times \phi_{1,2}\right)$ listed in Table 1. The absorption coefficients of the starting compounds (double‐caged **1**‐OMe‐H and **2**‐H‐H) are known, but the ones of the intermediates (single‐caged) are difficult to measure (only reaction constants were extracted from the fits). However, assuming that the absorption coefficient of the intermediate at the irradiation wavelength (365 nm) is half of the starting compound, the quantum yield of the second reaction was evaluated and listed in the table. The conversion quantum yield for the second step is slightly larger than for the first step. The uncaging efficiencies under irradiation with 365 nm wavelength for each step are 8–10 times higher for the *ONB* cage than for the *DiMeO‐ONB* cage.

**Table 1:** Reaction parameters of the photochemical reactions. Extinction coefficients and quantum yields are measured at irradiation wavelength of 365 nm.^[a]^

Compd.	Extinction coefficient of starting compound: $\epsilon_{SC}^{\lambda_{irr}}\left[\frac{1}{M\;cm}\right]$	Extinction coefficient of intermediate: $\epsilon_{Int}^{\lambda_{irr}}=\frac{1}{2}\epsilon_{SC}^{\lambda_{irr}}\;\left[\frac{1}{M\;cm}\right]$	$\epsilon_{SC}^{\lambda_{irr}}\times \phi_{1}$	$\epsilon_{Int}^{\lambda_{irr}}\times \phi_{2}$	Quantum yield of first reaction: $\phi_{1}$	Quantum yield of second reaction:$\phi_{2}$
**1**‐OMe‐H	8800	4400	44	32	0.005	0.007
**2**‐H‐H	575	287	28	16	0.049	0.056

[a] The *DiMeO‐ONB* analog shows a high signal (9.5 % relative to the signal after UV activation) after scanning once with only the STED beam, whereas the ONB‐2SiR analog shows only a very minor, negligible signal increase.

**Figure 3:**
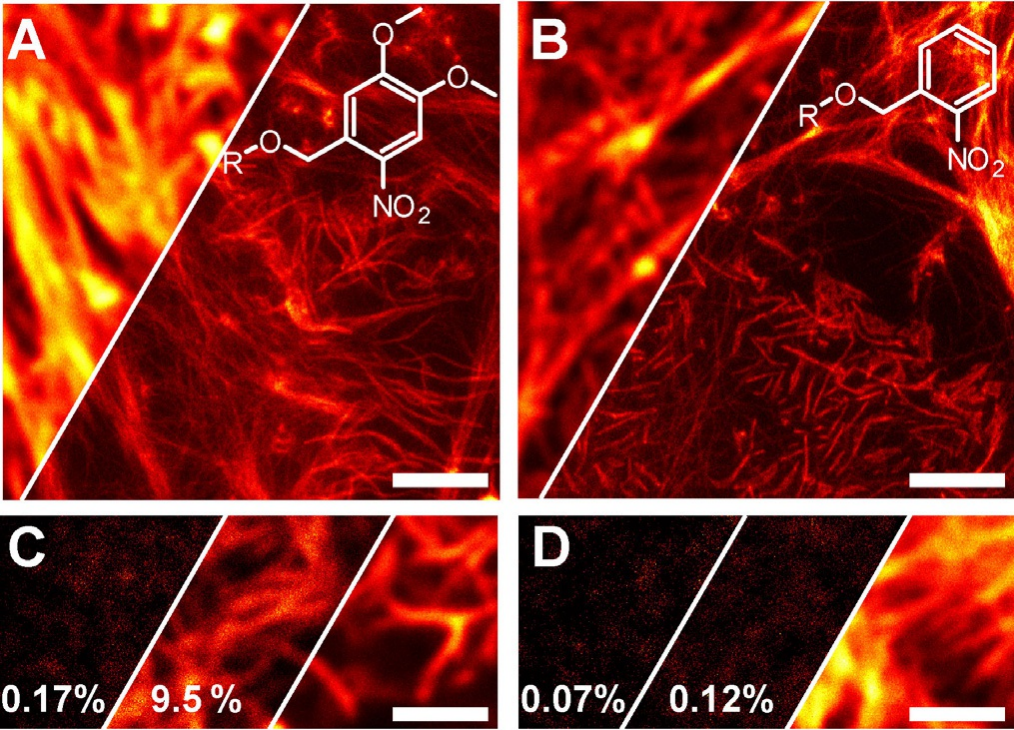
Images of actin filaments in fixed HeLa cells labelled with compounds **12**‐OMe‐Phalloidin (**A**, **C**) and **13**‐H‐Phalloidin (**B**, **D**). **A**, **B**: Confocal (left) and STED (right) images after activation with UV light below 400 nm wavelength. **C**, **D**: Confocal image sequence of the same sample region, (from left to right): after preparation; after one scan with exposure to STED light only; and after UV activation. The numbers in **C** and **D** show the signals relative to the signal after UV activation. Scale bar: 2 μm.

The phalloidin conjugates **12**‐OMe‐Phalloidin and **13**‐H‐Phalloidin were applied in fixed HeLa cells and imaged with a commercial STED microscope (Abberior Instruments) featuring a STED laser emitting pulses of ≈1.2 ns duration at 775 nm wavelength. The oil‐immersion objective lens had a numerical aperture of 1.4. The results are shown in Figure 3. The images before activation and the full‐size images are shown in Figure S**5**. ONB‐2SiR exhibits a negligible signal before UV activation, whereas the *DiMeO‐ONB* analogue shows a significantly higher (but for most applications still acceptable) signal under the same conditions. Both conjugates were activated with a broadband 400 nm LED to saturation and imaged by STED microscopy. Since both dyes share the same activated chromophore, the images exhibit the same resolution. The two‐photon activation (2PA) of the dyes by the STED light was tested by scanning a fresh region several times in the following sequence: (i) first confocal image scan directly after preparation; (ii) scan with STED light only (image not shown); (iii) second confocal image scan; (iv) UV activation (image not shown); (v) third confocal image scan. In step (ii) the STED beam had the same power as used for the STED images in Figure 3 A–**B**, namely approx. 90 mW time‐averaged power, amounting to ≈430 MW cm^−2^ of pulse peak intensity.

The blue shift in the absorption of the *ONB* (with respect to *DiMeO‐ONB*) resulted in a remarkable absence of 2PA with the nanosecond‐pulsed 775 nm STED beam. The undesired residual activation of the *ONB* analogue, as well as the fluorescence before UV activation, remained negligible compared to the signal after UV activation. The activation of the *ONB* analogue was also tested at 405 nm wavelength, which has been reported to be an efficient activation light for the *DiMeO‐ONB* cage.^[13d]^ Only sparse activation was observed (Figure S6A, B).

To allow the labelling of different cellular structures, the NHS‐ester of the *ONB* analogue **11**‐H**‐**Su was used to produce antibody conjugates with primary/secondary antibodies. First attempts using a standard labeling protocol (<3 % DMSO or DMF in aqueous buffer) were unsuccessful, probably due to the poor solubility of the dyes. The solubilizing linker seems to be insufficient on its own to provide good conjugation of the caged dye. The same results were obtained with the *DiMeO‐ONB* variant **10**‐OMe‐Su. Since further solubilizing chemical modifications would require a new synthesis route, we opted for adapting the conjugation and purification routines.

**Figure 4:**
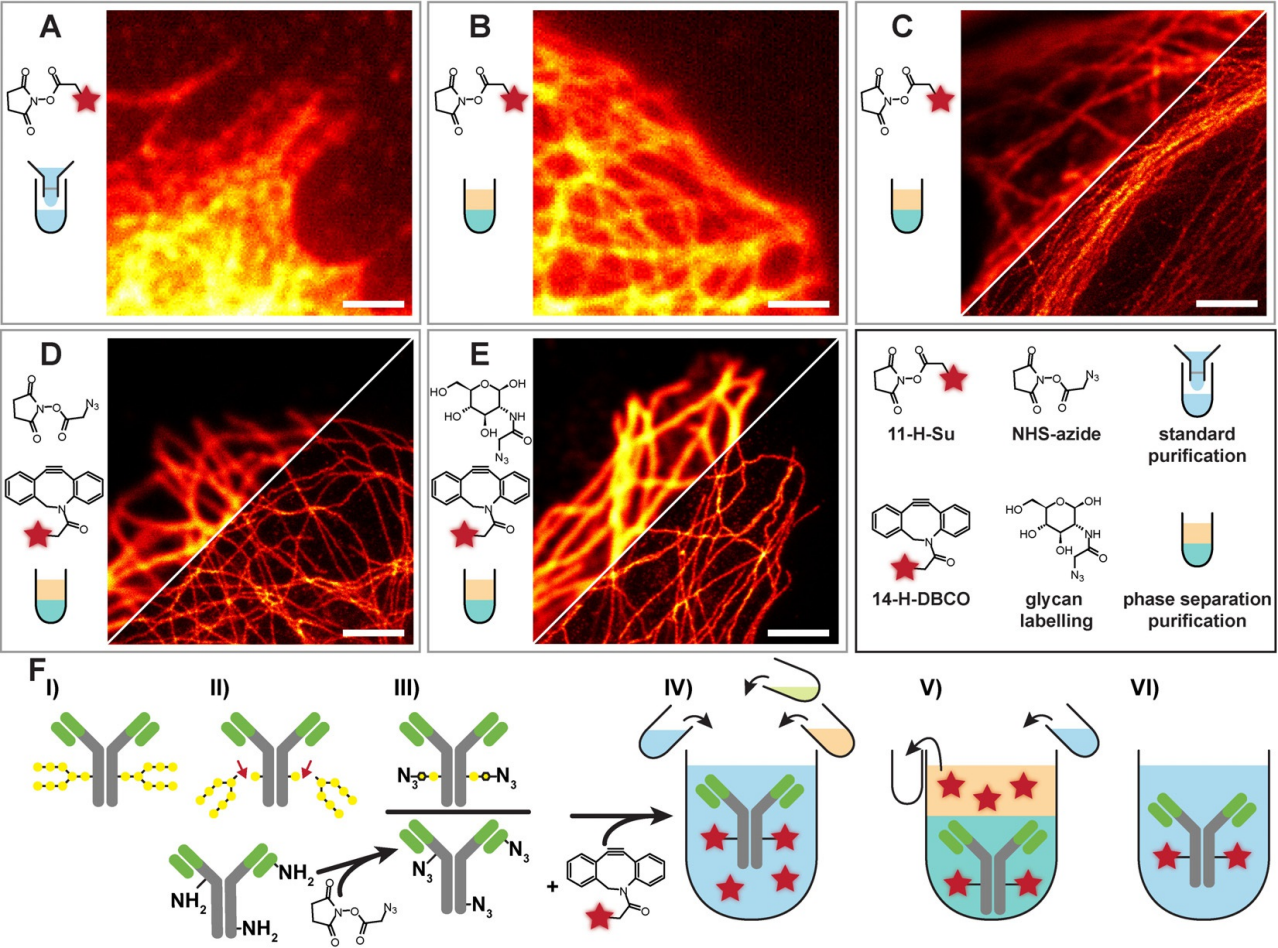
Fixed U2OS cells labeled with primary antibody against tubulin and secondary antibody conjugated with **11**‐H**‐**Su (**A**–**C**) or **14**‐H‐DBCO (**D**, **E**) using standard (**A**) or phase‐separation purification (**B**–**E**) with NHS (**A**–**C**), NHS‐azide (**D**) or glycan‐azide (**E**). **F** shows the antibody modification and purification protocol: **I**–**III)** cleavage of glycans, attachment of azide modified sugars or azide introduction through NHS‐azide, **IV**) reaction with **14**, addition of water, saturated ammonium sulfate solution and *tert*‐butanol, **V**) removal of organic phase, addition of buffer, **VI**) final antibody solution. Scale bar: 2 μm.

We increased the amount of organic solvent (DMSO or DMF) in the coupling reaction to 30 % to facilitate the dissolution and reaction of the lipophilic dye. After conjugation, the antibodies aggregated, and the unreacted dye could not be completely separated from the protein using size‐exclusion chromatography, dialysis or MWCO spin filters. Cells stained with these antibodies were used for fluorescence microscopy as shown in Figure 4 A. The labelled microtubules are weakly visible in the wide‐field image with a high intra‐ and extracellular background, which can be attributed to the unreacted dye. For obtaining better performing antibodies, we first optimized the purification of the conjugates, to remove the unreacted dye from the antibody solution.

In order to separate the lipophilic dye from the labelled antibodies, we developed a new separation technique based on the three phase partitioning (TPP) method.^[18]^ We used *tert*‐butanol as an organic co‐solvent and saturated ammonium sulfate solution to induce a phase separation in the *tert*‐butanol/DMF/water system. The lipophilic dye accumulates in the organic (*tert*‐butanol) phase and can be removed (together with most of the DMF), whereas the antibody remains in the aqueous phase. Before saturated (NH_4_)_2_SO_4_ and *tert*‐butanol were added to the antibody dye solution, the mixture was diluted with distilled water to compensate the loss by the water content of the organic phase. The amounts of distilled water, saturated (NH_4_)_2_SO_4_ and *tert*‐butanol can be adjusted to obtain the desired volume of the aqueous phase (to concentrate or dilute the protein), salt concentration (salts partition mainly into the aqueous phase) and volume of the organic phase. The salt concentration was kept low and the purified antibodies were diluted with buffer, in order to prevent the antibody from precipitating. Wide‐field, confocal and STED images using labelled secondary antibodies purified by this method are shown in Figure 4 B **–C**. The wide‐field image shows dense labelling of the microtubules without intra‐ or extracellular background. The STED images are punctuated, whereas the confocal images show homogenous labelling. Variations in the ratio between antibody and NHS‐ester **11**‐H‐Su did not significantly improve the image quality. The aggregation of the antibodies was still observed, even with the new purification method. The punctuated STED images also indicated a low DOL.

With the new purification technique at hand, we started optimizing the conjugation procedure to improve the DOL and reduce the aggregation of the antibodies upon labelling. Since the desired aminolysis of NHS‐esters is compromised by hydrolysis, and the concentration of the dye in water is an important parameter, we exchanged the reactive groups to a hydrolytically stable azide alkyne pair in a strain‐promoted „click chemistry“. First, azide groups were introduced into antibodies by converting free amines to azides with *N*‐hydroxysuccinimidyl azidoacetate. The azide‐ containing antibodies were purified by size exclusion chromatography. Then, the modified antibodies reacted with a dye bearing a dibenzocyclooctene (DBCO) residue **14**‐H‐DBCO and were purified using the new method. The confocal and STED images obtained with these antibodies are shown in Figure 4 D. The structures appear densely labelled even in the STED images. However, partial precipitation of the protein after purification was still observed (which often happens in the course of labelling with hydrophobic dyes). The low water solubility of the dye putatively limits the maximal amount of dye attachable to the antibody before precipitation occurs. Thus, the DOL needs to be precisely controlled between the minimum, defined by the fluorescence needed for obtaining informative images, and the maximum levels, limited by aggregation. In order to control the DOL and target the conjugation to a specific site of the antibodies, we enzymatically modified the glycans on the heavy chain of the antibody.^[19]^ The number of glycan chains attached to the IgG antibodies, which is two for most species, then defines the DOL. Different types of antibodies (secondary and primary) were tested with this conjugation and purification technique, and no aggregation was observed, even after storage for months in solution. The confocal and STED images of tubulin labelled with primary and secondary antibodies are presented in Figure 4 E. Notice that no background is observed despite dense labelling of the structure.

The photoactivatable dye ONB‐2SiR conjugated to primary or secondary antibodies was further tested for different imaging applications. Although it was primarily designed for STED imaging, the dye can also be used in PALM. Figure S6C shows a PALM image obtained using 405 nm light for uncaging, which we show to induce sparse activation of the *ONB* caged fluorophore.

The resilience against 2PA protects the dye from bleaching and allows using ONB‐2SiR to extend the number of channels of a STED microscope equipped with a nanosecond‐pulsed 775 nm STED beam. First, an image is acquired with the signal from a normal fluorescent dye (i.e. uncaged). This dye is then bleached using intense excitation. Subsequently, a second caged dye is activated by UV light and imaged. The activated second dye may feature similar spectral properties as the first dye, such that it can be imaged under similar conditions as the first dye. Thereby, a second detection window can be added to any spectral channel of a fluorescence microscope. This method is particularly useful for STED microscopy with 775 nm STED wavelength as it neither requires attribution by the excitation and/or detection wavelengths of the fluorophores, nor by their fluorescence lifetimes, nor by the linear un‐mixing of their signals.

To test the concept for visualizing different cellular structures, one of the primary antibodies was conjugated with compound **14**‐H‐DBCO, and two others were labelled with primary and secondary antibodies decorated with Alexa 594 and Abberior STAR 635P fluorophores. The combinations of primary and secondary antibodies amplify the signals, whereas the primary antibodies decorated only with **14**‐H‐DBCO have two fluorophores per antibody via the glycan labeling, and produce darker images.

**Figure 5:**
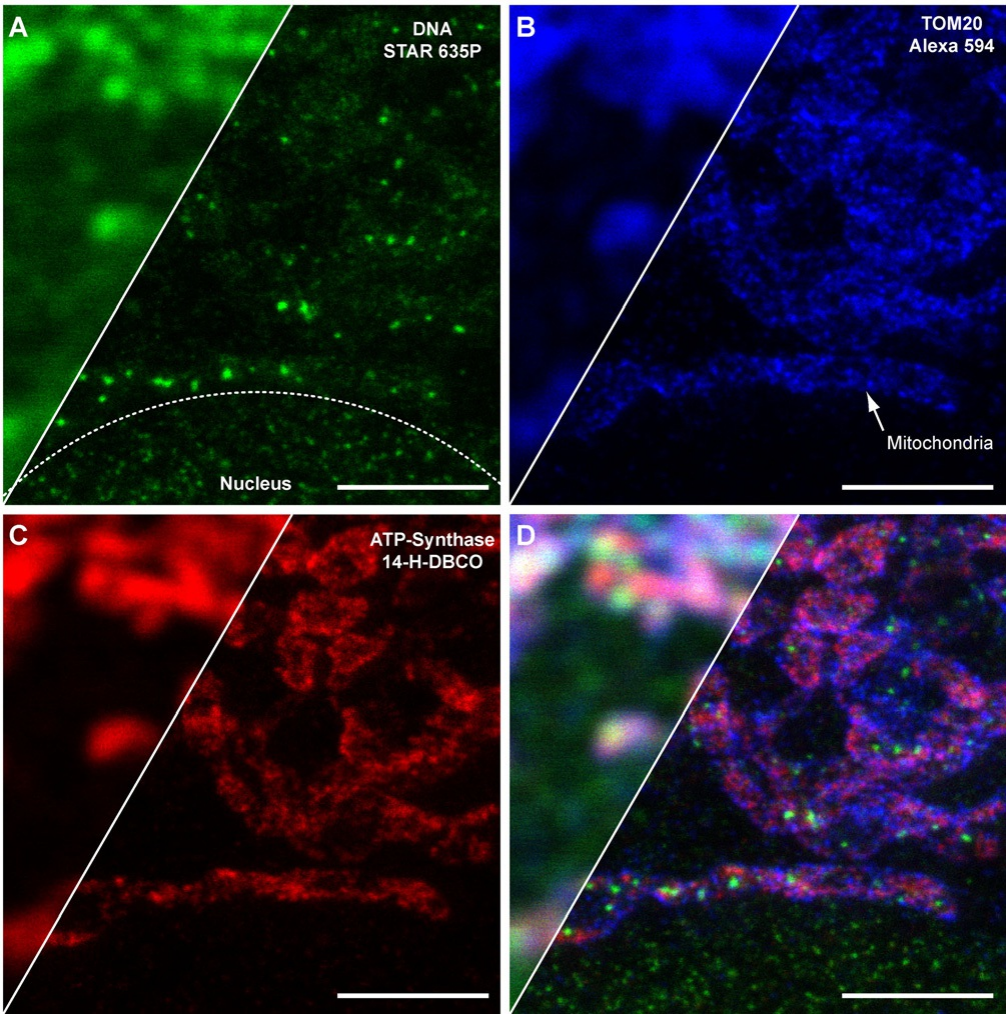
Confocal (left) and STED (right) images of fixed U2OS cells labelled with primary antibody against DNA (**A**: green), TOM20 (**B**: blue) and ATP‐Synthase B (**C**: red) labelled with **14**‐H‐DBCO (activated by UV light below 400 nm wavelength) and secondary antibodies labelled with STAR 635P (green) and Alexa 594 (blue). The images show mitochondri.a and a part of the nucleus (bottom of the images). An overlay of all colors is shown in **D**. Scale bar: 2 μm.

The three‐color images with these fluorophores are presented in Figure 5. The full images are shown in Figure S**7**. Although the ONB‐2SiR‐decorated primary antibodies are unamplified by secondary antibodies, the signal of ONB‐2SiR greatly exceeds any residual unbleached STAR 635P, which can be seen by the low signal after activation in the nucleus at the bottom of the image compared to the bright signal in the mitochondria (see Figure 5 C). The structure, labelled with primary antibody conjugated to ONB‐2SiR, shows a comparable image quality to the STAR 635P primary/secondary antibody‐labelled structure, even though the latter is with amplification.

## Conclusions

We synthesized a new photoactivatable SiR fluorophore (ONB‐2SiR) for STED microscopy, which is stable against two‐photon activation with the routinely applied STED pulses at 775 nm wavelength. The fluorophore is also suitable for single‐molecule localization microscopy, such as PALM, or the more recent nanoscopy called MINFLUX. The versatility of ONB‐2SIR should enable the same sample preparation for different microscopy techniques to realize spatial resolutions ranging from diffraction‐ to label‐limited. The ability to optimize sample preparation and access the labelled structure also with STED microscopy is very helpful for single‐molecule localization techniques as well. To compensate the intrinsic hydrophobicity of caged dyes like ONB‐2SIR, we introduced a new coupling and purification protocol that can be used to conjugate aggregating and poorly water‐soluble dyes to antibodies. The protocol provides definite and constant values of DOL and reduces the antibody aggregation while preserving their affinity.

## Conflict of interest

The authors declare no conflict of interest.

## Supporting information

As a service to our authors and readers, this journal provides supporting information supplied by the authors. Such materials are peer reviewed and may be re‐organized for online delivery, but are not copy‐edited or typeset. Technical support issues arising from supporting information (other than missing files) should be addressed to the authors.

Supplementary

## References

[1a] S. W. Hell , J. Wichmann , Opt. Lett. 1994, 19, 780–782; 19844443 10.1364/ol.19.000780

[1b] V. Westphal , S. O. Rizzoli , M. A. Lauterbach , D. Kamin , R. Jahn , S. W. Hell , Science 2008, 320, 246–249; 18292304 10.1126/science.1154228

[1c] S. Berning , K. I. Willig , H. Steffens , P. Dibaj , S. W. Hell , Science 2012, 335, 551. 22301313 10.1126/science.1215369

[2a] S. W. Hell , M. Kroug , Appl. Phys. B: Lasers and Opt. 1995, 60, 495–497;

[2b] S. Bretschneider , C. Eggeling , S. W. Hell , Phys. Rev. Lett. 2007, 98, 218103. 17677813 10.1103/PhysRevLett.98.218103

[3a] M. G. Gustafsson , Proc. Natl. Acad. Sci. USA 2005, 102, 13081–13086; 16141335 10.1073/pnas.0406877102 PMC1201569

[3b] R. Heintzmann , T. M. Jovin , C. Cremer , J. Opt. Soc. Am. A 2002, 19, 1599–1609. 10.1364/josaa.19.001599 12152701

[4] S. W. Hell , S. Jakobs , L. Kastrup , Appl. Phys. A 2003, 77, 859–860.

[5a] M. J. Rust , M. Bates , X. Zhuang , Nat. Methods 2006, 3, 793–796; 16896339 10.1038/nmeth929 PMC2700296

[5b] M. Bates , B. Huang , G. T. Dempsey , X. Zhuang , Science 2007, 317, 1749–1753. 17702910 10.1126/science.1146598 PMC2633025

[6a] E. Betzig , G. H. Patterson , R. Sougrat , O. W. Lindwasser , S. Olenych , J. S. Bonifacino , M. W. Davidson , J. Lippincott-Schwartz , H. F. Hess , Science 2006, 313, 1642–1645; 16902090 10.1126/science.1127344

[6b] S. T. Hess , T. P. K. Girirajan , M. D. Mason , Biophys. J. 2006, 91, 4258–4272. 16980368 10.1529/biophysj.106.091116 PMC1635685

[7a] J. Fölling , M. Bossi , H. Bock , R. Medda , C. A. Wurm , B. Hein , S. Jakobs , C. Eggeling , S. W. Hell , Nat. Methods 2008, 5, 943–945; 18794861 10.1038/nmeth.1257

[7b] I. Testa , C. A. Wurm , R. Medda , E. Rothermel , C. von Middendorf , J. Fölling , S. Jakobs , A. Schönle , S. W. Hell , C. Eggeling , Biophys. J. 2010, 99, 2686–2694. 20959110 10.1016/j.bpj.2010.08.012 PMC2956215

[8] M. Heilemann , S. van de Linde , M. Schuttpelz , R. Kasper , B. Seefeldt , A. Mukherjee , P. Tinnefeld , M. Sauer , Angew. Chem. Int. Ed. 2008, 47, 6172–6176; 10.1002/anie.200802376 18646237

[9a] K. C. Gwosch , J. K. Pape , F. Balzarotti , P. Hoess , J. Ellenberg , J. Ries , S. W. Hell , Nat. Methods 2020, 17, 217–222; 31932776 10.1038/s41592-019-0688-0

[9b] F. Balzarotti , Y. Eilers , K. C. Gwosch , A. H. Gynna , V. Westphal , F. D. Stefani , J. Elf , S. W. Hell , Science 2017, 355, 606–612. 28008086 10.1126/science.aak9913

[10] V. N. Belov , C. A. Wurm , V. P. Boyarskiy , S. Jakobs , S. W. Hell , Angew. Chem. Int. Ed. 2010, 49, 3520–3523; 10.1002/anie.201000150 20391447

[11] G. A. Krafft , W. R. Sutton , R. T. Cummings , J. Am. Chem. Soc. 1988, 110, 301–303.

[12] B. Roubinet , M. Bischoff , S. Nizamov , S. Yan , C. Geisler , S. Stoldt , G. Y. Mitronova , V. N. Belov , M. L. Bossi , S. W. Hell , J. Org. Chem. 2018, 83, 6466–6476. 29749224 10.1021/acs.joc.8b00756

[13a] L. M. Wysocki , J. B. Grimm , A. N. Tkachuk , T. A. Brown , E. Betzig , L. D. Lavis , Angew. Chem. Int. Ed. 2011, 50, 11206–11209; 10.1002/anie.201104571 PMC3588110 21953685

[13b] V. N. Belov , G. Y. Mitronova , M. L. Bossi , V. P. Boyarskiy , E. Hebisch , C. Geisler , K. Kolmakov , C. A. Wurm , K. I. Willig , S. W. Hell , Chem. Eur. J. 2014, 20, 13162–13173; 25196166 10.1002/chem.201403316

[13c] J. B. Grimm , B. P. English , H. Choi , A. K. Muthusamy , B. P. Mehl , P. Dong , T. A. Brown , J. Lippincott-Schwartz , Z. Liu , T. Lionnet , L. D. Lavis , Nat. Methods 2016, 13, 985–988; 27776112 10.1038/nmeth.4034

[13d] J. B. Grimm , T. Klein , B. G. Kopek , G. Shtengel , H. F. Hess , M. Sauer , L. D. Lavis , Angew. Chem. Int. Ed. 2016, 55, 1723–1727; 10.1002/anie.201509649 PMC4736676 26661345

[14] J. G. Danzl , S. C. Sidenstein , C. Gregor , N. T. Urban , P. Ilgen , S. Jakobs , S. W. Hell , Nat. Photonics 2016, 10, 122–128.

[15] G. Lukinavičius , K. Umezawa , N. Olivier , A. Honigmann , G. Yang , T. Plass , V. Mueller , L. Reymond , I. R. Corrêa, Jr. , Z.-G. Luo , C. Schultz , E. A. Lemke , P. Heppenstall , C. Eggeling , S. Manley , K. Johnsson , Nat. Chem. 2013, 5, 132–139. 23344448 10.1038/nchem.1546

[16a] G. Kostiuk , J. Bucevičius , R. Gerasimaitė , G. Lukinavičius , J. Phys. D: Appl. Phys. 2019, 52, 504003;

[16b] T. Stephan , A. Roesch , D. Riedel , S. Jakobs , Sci. Rep. 2019, 9, 12419. 31455826 10.1038/s41598-019-48838-2 PMC6712041

[17a] R. G. Dyer , K. D. Turnbull , J. Org. Chem. 1999, 64, 7988–7995;

[17b] G. E. Negri , T. J. Deming , ACS Macro Lett. 2016, 5, 1253–1256. 10.1021/acsmacrolett.6b00715 35614735

[18] C. Dennison , R. Lovrien , Protein Expression Purif. 1997, 11, 149–161. 10.1006/prep.1997.0779 9367811

[19a] J. Sjögren , W. B. Struwe , E. F. J. Cosgrave , P. M. Rudd , M. Stervander , M. Allhorn , A. Hollands , V. Nizet , M. Collin , Biochem. J. 2013, 455, 107–118; 23865566 10.1042/BJ20130126 PMC3778708

[19b] B. Ramakrishnan , P. K. Qasba , J. Biol. Chem. 2002, 277, 20833–20839. 11916963 10.1074/jbc.M111183200

