# Supplementary material for: Photoactivatable Fluorophore for Stimulated Emission Depletion (STED) Microscopy and Bioconjugation Technique for Hydrophobic Labels
Source: Chemistry. 2020 Nov 26;27(1):451–8. doi: 10.1002/chem.202004645 (PMC7839434; doi:10.1002/chem.202004645)
Supplement: Supplementary file 1 — Supplementary [file CHEM-27-451-s001.pdf]

Chemistry–A European Journal

Supporting Information

Photoactivatable Fluorophore for Stimulated Emission Depletion (STED) Microscopy and Bioconjugation Technique for Hydrophobic Labels

Michael Weber,[a] Taukeer A. Khan,[a] Lukas J. Patalag,[a, c] Mariano Bossi,[b] Marcel Leutenegger,[a] Vladimir N. Belov,[a] and Stefan W. Hell*[a, b]

Table of Contents

Experimental Procedures	2
Scheme S1 and S2	4
Experimental procedures and analytical data	5
Scheme S3	8
Experimental procedures and analytical data	9
Scheme S4	11
Experimental procedures and analytical data	11
Scheme S5	14
Experimental procedures and analytical data	15
Supplementary figures	16
1H and 13C NMR and HPLC spectra	22
Supplementary references	35

Experimental Procedures

Thin layer chromatography: Analytical TLC (normal phase) was performed on Merck Millipore ready-to-use aluminium sheets coated with silica gel 60 (F_{254}). Compounds were detected by exposing TLC plates to UV-light (254 or 366 nm).

Preparative flash column chromatography: Flash column chromatography was performed using regular silica gel 60 (40−63 µm) from Macherey-Nagel, or cartridges from Interchim (PF-SIHC, 15 µM, 25 or 40 g SiO_2), or Teledyne Isco (RediSepRf, 35 µM, 24 or 40 g SiO_2). Automated separations were performed with an Isolera One system (Biotage AG, Sweden) using the type of cartridge and solvent gradient indicated.

High-performance liquid chromatography: Analytical HPLC was performed on a Knauer Azura liquid chromatography system with a binary P 6.1L pump, UV diode array detector DAD 6.1L, an injection valve with a 20 µL loop and two electrical switching valves V 2.1S with 6-port multiposition valve head. Analytical columns: Knauer Eurospher II 100-5 C18, 5 µm, 150×4 mm or Interchim Uptisphere Strategy C18-HQ, 10 µm, 250×4.6 mm, flow rate 1.2 mL/min, unless stated otherwise.

Preparative HPLC was performed on an Interchim puriFlash 4250 2X preparative HPLC/Flash hybrid system (Interchim) with a 5 mL injection loop, a 200-600 nm UV-Vis detector and an integrated ELSD detector. Preparative column: Interchim Uptisphere Strategy C18-HQ 10 µm, 250×21.2 mm, flow rate: 20 mL/min, unless specified otherwise.

NMR spectra were recorded at 25°C with an Agilent 400-MR spectrometer at 400 MHz (^{1}H), 376.4 MHz (^{19}F), and 100.6 MHz (^{13}C). ^{13}C NMR spectra were also acquired with a Bruker Avance III HD 500 MHz (BBO Prodigy probe) instrument. Chemical shifts (δ) are reported in ppm. All ^{1}H spectra are referenced to tetramethylsilane (TMS; δ = 0 ppm) using the signals of added TMS (0.03% v/v) or the residual protons of $CHCl_3$ (7.26 ppm) in $CDCl_3$, CHD_2OD (3.31 ppm) for CD_3OD, DMSO-d_5 (2.50 ppm) for DMSO-d_6.^{13}C NMR spectra are referenced to TMS (δ = 0 ppm) using the signals of added TMS (0.03% v/v) or the solvent: $CDCl_3$ (77.16 ppm), CD_3OD (49.00 ppm), DMSO-d_6 (39.52 ppm). Multiplicities of signals are described as follows: s = singlet, d = doublet, t = triplet, q = quartet, p = pentet, m = multiplet or overlap of non-equivalent resonances; br = broad signal. Coupling constants (J) are given in Hz.
ESI-MS were recorded on a Varian 500-MS spectrometer (Agilent). ESI-HRMS were recorded on a MICROTOF spectrometer (Bruker) equipped with ESI ion source (Apollo) and direct injector with LC autosampler Agilent RR 1200.

Spectroscopy: Stocks solutions (2-3 mM) of compounds **1**-OMe-H, **2**-H-H, and **3**, were prepared in acetonitrile. Final dilutions for spectroscopic characterization and irradiation experiments were prepared in 1:1 mixtures of acetonitrile and phosphate buffer at a concentration of 10-20 µM. All measurements were performed in quartz cuvettes with four clear faces (Hellma GmbH & Co.). Absorption and emission spectra were recorded in a Cary Series UV-Vis-NIR Spectrophotometer (Agilent Technologies), and a Cary Eclipse Fluorescence Spectrophotometer (Agilent Technologies), respectively. Fluorescence lifetime was measured in a Fluorescence Lifetime Spectrometer (FluoTime 300, PicoQuant). Irradiation experiments were performed in a home-build setup[1], using a 365 nm LED as irradiation source (M365-L2, Thorlabs), a Deuterium/Xenon lamp (DH-2000-BAL, Ocean Optics) as an illumination source and a diode array spectrometer (FLAME-S-UV-VIS-ES, Ocean Optics). The intensity of the irradiation light was calibrated with a chemical actinometer (Azobenzene in MeOH). The samples were kept at 20 °C and continuously stirred with a Peltier-based temperature control (Luma 40, Quantum Northwest, Inc.). The absorption of the samples was recorded at a right angle with respect to the irradiation source, at fixed irradiation intervals until complete conversion to the final product. At fixed intervals, a small amount of sample was extracted to perform LC-MS experiments (Shimadzu LCMS-2020).

Antibody Conjugation and Purification: All necessary chemicals for antibody conjugation were purchased from Sigma-Aldrich. For NHS-ester conjugated antibodies 400 µL (~1 mg) secondary goat anti rabbit antibody (111-005-003, Dianova) was mixed with 40 µL 1 M $NaHCO_3$ and 200 µL DMF premixed with 100 µg NHS-ester dye. After one hour reaction under rigorous stirring, the sample was purified using either a standard size exclusion purification with a PD-10 (GE Healthcare) column or by phase separation. For the phase separation purification 1.2 mL distilled water, 182 µL saturated $(NH_4)_2SO_4$ and 1.82 mL *tert*-butanol was added, quickly vortexed and separated into two phases after a short centrifugation pulse. The aqueous phase (~400 µL) was diluted to two times its volume with PBS. The conjugated antibodies were aliquoted and stored at -20 °C. The antibodies did not show any degradation even after months of storage at 5 °C.

To modify antibodies with azide-groups, 1 mg (~400 µL) secondary goat anti rabbit antibody was mixed with 40 µL 1 M $NaHCO_3$ and 1.32 µL DMSO premixed with 13.2 µg *N*-hydroxysuccinimidyl azidoacetate. The mixture was stirred for 1 h and purified using a PD-10 (GE Healthcare) size exclusion column. TBS was used as elution buffer. The glycan labelled antibodies (250 µg) were produced using the commercial GlyClick (Genovis) enzyme system and eluted in TBS (150 mM NaCl, 50 mM Tris-HCl, pH 7.6). To the final volume of 200 µL (1 mg *N*-hydroxysuccinimidyl azidoacetate modified antibodies or 250 µg glycan azide antibodies), 50 µL DMF and 50 µg of azide reactive DBCO-dye was added. The mixture was stirred over night at room temperature. Then it was diluted with 600 µL distilled water, 90 µL saturated $(NH_4)_2SO_4$ and 900 µL *tert*-butanol, quickly vortexed and separated into two phases after a short centrifugation pulse. The aqueous phase (~ 200 µL) was diluted to two times its volume with TBS. Optionally, the buffer can be exchanged using a regenerated cellulose MWCO spin filter (Microcon YM-50 50 kDa MWCO). The conjugated antibodies were aliquoted and stored at -20 °C.

Cell fixation and staining: HeLa and U2OS cells were grown on coverslips and then fixed with 4% (w/v) paraformaldehyde for 15 min or with cold methanol (-20°C) for 4 min. The cells were permeabilizied with 0.1% Triton X-100 in PBS for 5 min. After blocking with 2% (w/v) BSA in PBS, the cells were treated with antibodies diluted in the same buffer for 1 h. Between multiple antibody treatments, the cells were washed with 2% (w/v) BSA in PBS. The phalloidin conjugates were dissolved in DMSO and applied on the fixed cells for 1 h at a final concentration of ~100 µg/mL in PBS. The samples were mounted in PBS (wide-field, confocal and STED imaging) or 20 mM Hepes buffer (pH 7 + 150 mM NaCl) and sealed using silicon resin (TwinSeal Picodent). Used antibody: ATP-Synthase B (ab5432, Abcam), TOM 20 (sc-11415, SantaCruz), DNA (61014, Progen), alpha-Tubulin (ab18251, Abcam).

Imaging: The wide-field images were taken on a Leica DM6000 microscope (Leica HCX Pl APO 100×/1.4-0.7 NA oil) using an A4 filter cube (Leica, excitation: 360/40 nm, detection: 470/40 nm) for activation and APC HC filter cube (AHF F36-540, excitation: 600/37 nm, detection: 675/67 nm) for imaging.
For the STED images a commercial Abberior Instruments Expert Line microscope equipped with a UPLANSAPO 100×/1.4 NA oil-immersion objective and a 775 nm 40 MHz STED laser of ~ 1.2 ns pulse duration was used. The fluorophores were excited with a 640 nm and a 561 nm 40 MHz pulsed laser and detected in two color channels 615/20 nm and 685/70 nm. Between the acquisitions of the multiplexed color channel, the sample was bleached using a 595 nm laser. The caged dyes were activated using a wide-field 405 nm led illumination (CoolLED) with a broad spectrum extending far below 400 nm.
The single molecule and PALM images were performed on a home-built microscope and analyzed as previously described[2]. In brief, the sample was illuminated through an oil immersion objective (Olympus UPLANSAPO 100×/1.4 NA oil) using a 642 nm laser in total internal reflection (TIRF) mode and imaged onto an EMCCD camera using a bandpass emission filter (700/75 nm). The molecules were sparsely activated using a 405 nm laser.

Results and Discussion

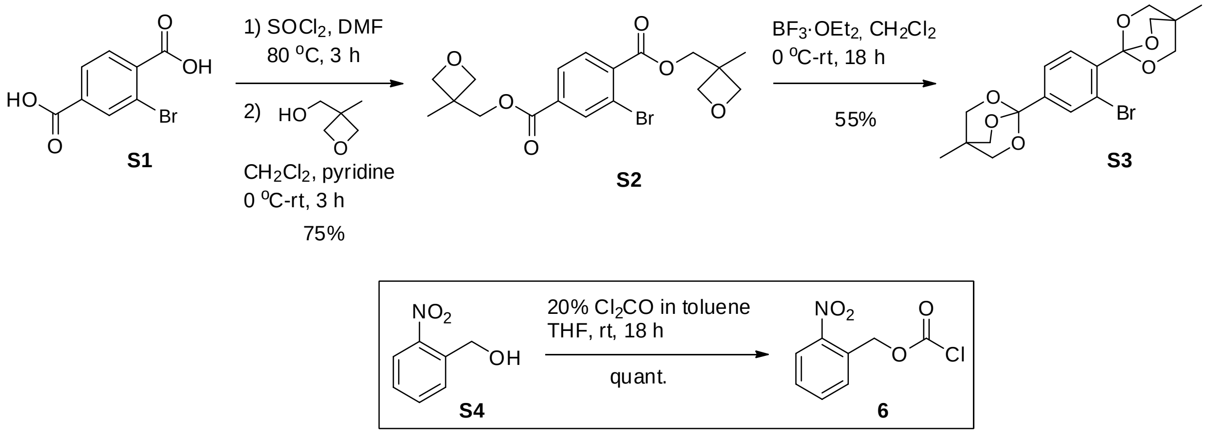

Scheme S1. Synthesis of orthoester **S3** and *o*-nitrobenzyl chloroformate **6**

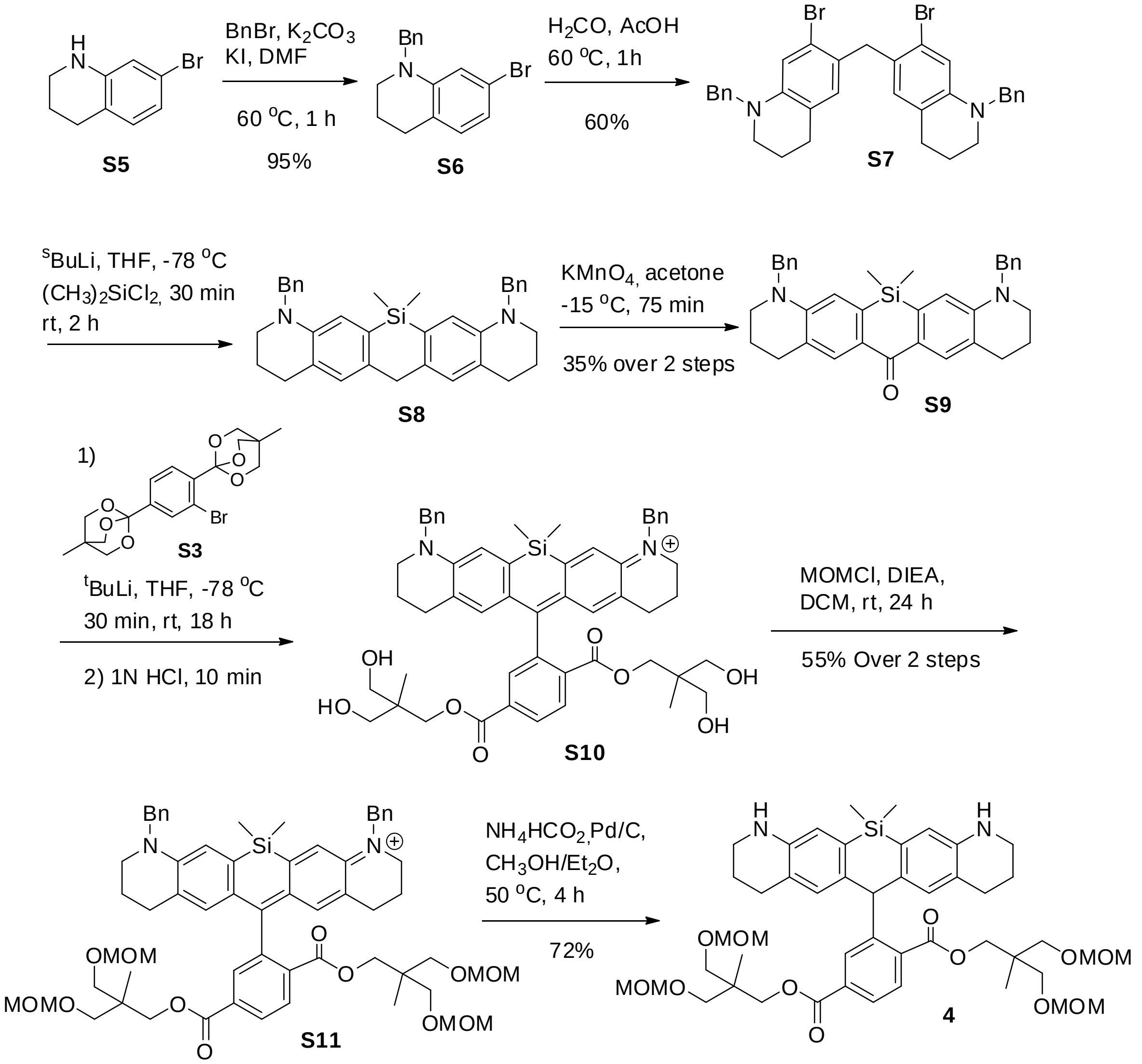

Scheme S2. Synthesis of leuco-Si-rhodamine **4**

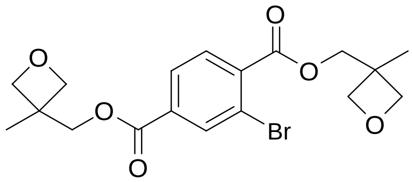

Bis((3-methyloxetan-3-yl)methyl) 2-bromoterephthalate (S2):[3] A mixture of 2-bromoterephthalic acid (3.0 g, 12.2 mmol), thionyl chloride (10.0 mL), and DMF (2 drops) was stirred at reflux for 3 h. The mixture was concentrated in vacuum and co-evaporated twice with toluene. The crude acid chloride was taken up in CH_2Cl_2 (18 mL) and was added to a solution of 3-methyl-3-oxetanemethanol (3.12 g, 30.6 mmol, 2.5 eq) and pyridine (2.2 mL, 61 mmol, 5 eq) in CH_2Cl_2 (12 mL) at 0 °C, and stirred for 10 min. The ice bath was removed, and the reaction mixture was stirred at room temperature for 3 h. The reaction mixture was diluted with water and extracted with CH_2Cl_2 (3 ×). The combined organic solutions were washed with brine, dried (Na_2SO_4), filtered, and evaporated. Flash chromatography (0–75% EtOAc/hexanes, linear gradient) afforded the diester (**S2**) as a colourless gum (4.90 g, 90%). The NMR spectra corresponded to the published data [3].

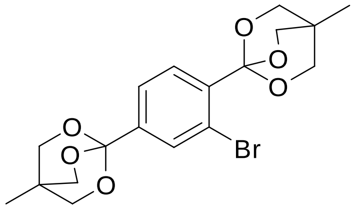

1,1'-(2-Bromo-1,4-phenylene)bis(4-methyl-2,6,7-trioxabicyclo[2.2.2]octane) (S3):[3] A solution of diester, S**2** (4.85 g, 11.7 mmol) in CH_2Cl_2 (30 mL) was cooled to 0 °C under nitrogen, $BF_3{\cdot}OEt_2$ (0.81 mL, 5.9 mmol, 0.5 eq) was added; the reaction mixture was stirred for 10 min, allowed to warm to room temperature and stirred for 18 h. Et_3N (4 mL) was added, and the solution stirred for 15 min, concentrated in vacuum and purified by flash chromatography (0–20% EtOAc/hexanes, linear gradient, with constant 40% v/v CH_2Cl_2 and 1% Et_3N) to provide 2.8 g (57%) of **S3** as a white solid. ^{1}H NMR (DMSO-*d*$_6$, 400 MHz) δ 7.63 (d, *J* = 8.3 Hz, 1H), 7.62 (d, *J* = 1.6 Hz, 1H), 7.42 (dd, *J* = 8.2, 1.7 Hz, 1H), 3.98 (s, 6H), 3.97 (s, 6H), 0.81 (s, 3H), 0.80 (s, 3H). Other analytical data were identical to the reported values [3].

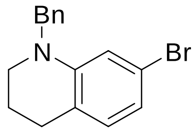

1-Benzyl-7-bromo-1,2,3,4-tetrahydroquinoline (S6): 7-Bromo-1,2,3,4-tetrahydroquinoline[1] (6.4 g, 30 mmol, 1 eq), benzyl bromide (4.0 mL, 36 mmol, 1.2 eq), K_2CO_3 (12.5 g, 90.5 mmol, 3 eq), and KI (1 g, 6 mmol, 0.2 eq) were combined in DMF (45 mL) and stirred at 60 °C for 6 h. The reaction mixture was diluted with water and extracted with EtOAc (3 ×). The combined organic solutions were washed with water and brine, dried (Na_2SO_4), filtered, and concentrated in vacuo. Flash chromatography (0–15% Et_2O/hexanes, linear gradient) on regular SiO_2 (100 g) yielded compound **S6**[2] (8.4 g, 92%) as a gum that crystallized into an off-white, low-melting solid upon standing. ^{1}H NMR ($CDCl_3$, 400 MHz) δ 7.34 – 7.30 (m, 2H), 7.27 – 7.20 (m, 3H), 6.80 (d, *J* = 7.9 Hz, 1H), 6.66 (dd, *J* = 7.9, 1.9 Hz, 1H), 6.62 (d, *J* = 1.9 Hz, 1H), 4.44 (s, 2H), 3.38 – 3.30 (m, 2H), 2.73 (t, *J* = 6.3 Hz, 2H), 1.99 – 1.91 (m, 2H).

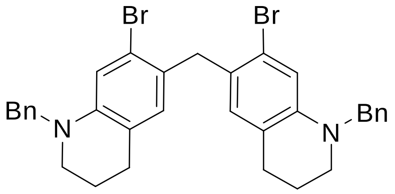

Bis(1-benzyl-7-bromo-1,2,3,4-tetrahydroquinolin-6-yl)methane (S7):[4] 1-Benzyl-7-bromo-1,2,3,4-tetrahydroquinoline **S6** (3.3 g, 10.9 mmol) was taken up in AcOH (15 mL). Formaldehyde (37% w/w in H_2O, 2.2 mL, 29 mmol, 2.7 eq) was added dropwise, and the solution stirred at 60 °C for 1 h. The reaction mixture was concentrated in vacuo to remove AcOH, diluted with saturated $NaHCO_3$, and extracted with EtOAc (3 ×). The combined organic solvents were washed with saturated $NaHCO_3$ and brine, dried (Na_2SO_4), filtered, and evaporated. The residue was triturated with Et_2O to obtain a solid which was isolated by filtration, washed with Et_2O, and dried to provide **S7** as an off-white solid[3] (2.5 g, 75%). ^{1}H NMR ($CDCl_3$, 400 MHz) δ 7.37 – 7.29 (m, 4H), 7.26 – 7.24 (m, 6H), 6.72 (s, 2H), 6.64 (s, 2H), 4.43 (s, 4H), 3.88 (s, 2H), 3.28 (t, *J* = 6.3 Hz, 4H), 2.67 (t, *J* = 6.3 Hz, 4H), 1.97 – 1.91 (m, 4H).

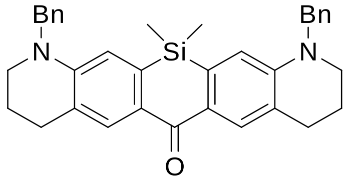

1,11-Dibenzyl-13,13-dimethyl-1,2,3,4,8,9,10,11-octahydrosilino[3,2-g:5,6-g']diquinolin-6(13*H*)-one (S9): A solution of dibromide **S7** (1.00 g, 1.62 mmol) in THF (ca. 10 mL) was cooled to −78 °C (in a dry ice – acetone bath) under nitrogen. *sec*-Butyllithium (1.4 M in cyclohexane, 3.5 mL, 4.9 mmol, 3.0 eq) was added, and the reaction mixture stirred at −78 °C for 30 min. Dichlorodimethylsilane (0.36 mL, 2.9 mmol, 1.8 eq) was then added dropwise. The dry ice – bath was removed, and the reaction mixture stirred at room temperature for 2 h. Then saturated aq. NH_4Cl was added carefully with stirring (5 mL), the reaction mixture diluted with water, and extracted with EtOAc (3 ×). The combined organic solutions were washed with brine, dried (Na_2SO_4), filtered, and concentrated in vacuum to afford compound **S8** as a greenish-brown material. The crude substance (**S8**) was taken up in acetone (15 mL) and cooled to −15 °C. $KMnO_4$ (513 mg, 3.2 mmol, 2 eq) was added in 4 equal portions at 15 min intervals (45 min in total) under vigorous stirring. After stirring for another 30 min at −15 °C, the brown mixture was diluted with CH_2Cl_2, filtered through Celite, and concentrated. Flash chromatography on silica gel (15 g, 5–40% EtOAc/hexanes, linear gradient) provided 319 mg (37% over 2 steps) of ketone (**S9**) as a yellow solid. ^{1}H NMR ($CDCl_3$, 400 MHz) δ 8.10 (s, 2H), 7.33 – 7.28 (m, 4H), 7.25 – 7.21 (m, 6H), 6.56 (s, 2H), 4.57 (s, 4H), 3.45 – 3.42 (m, 4H), 2.88 (t, *J* = 6.2 Hz, 4H), 2.04 – 1.98 (m, 4H), 0.10 (s, 6H); ^{13}C NMR ($CDCl_3$, 101 MHz) δ 185.09, 147.55, 138.5, 138.0, 130.6, 129.6, 128.7, 127.1, 126.6, 123.7, 113.6, 54.9, 50.2, 28.2, 22.0, -1.4. HRMS (ESI) calcd for $C_{35}H_{36}N_2OSi$ $[M+H]^+$ 529.2670, found 529.2669.

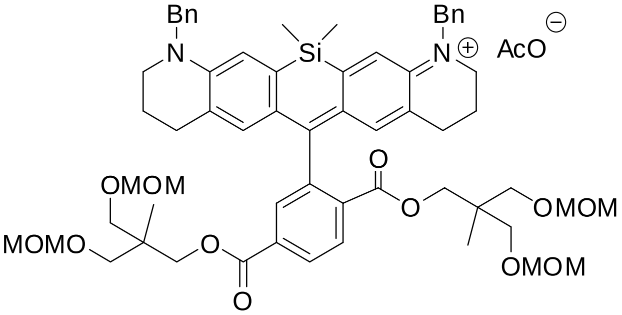

1,11-Dibenzyl-6-(2,5-bis((3-(methoxymethoxy)-2-((methoxymethoxy)methyl)-2-methylpropoxy)carbonyl)phenyl)-13,13-dimethyl-2,3,4,8,9,10,11,13-octahydrosilino[3,2-g:5,6-g']diquinolin-1-ium acetate (S11): A solution of bromide **S3** (0.975 g, 2.36 mmol, 5 eq) in THF (58 mL) was cooled to −78 °C under nitrogen. *tert*-Butyllithium (1.7 M in pentane, 2.8 mL, 4.7 mmol, 10 eq) was added dropwise, and the reaction stirred for 30 min at −78 °C. Then a solution of ketone **S9** (250 mg, 0.47 mmol, 1 eq) in THF (6 mL) was added dropwise. The reaction mixture was allowed to slowly warm to room temperature while stirring overnight (18 h). The reaction mixture was "quenched" by slow addition of 1 M aq HCl (13 mL), which caused an immediate colour change from pale orange to deep blue. After stirring for 10 min, the mixture was diluted with water and extracted with 15% *i*PrOH/$CHCl_3$ (3 ×). The combined organic solutions were dried (Na_2SO_4), filtered, and concentrated under vacuum. Silica gel chromatography (0–20% MeOH/CH_2Cl_2, linear gradient, with constant 1% v/v AcOH additive) provided the 3-hydroxy-2-(hydroxymethyl)-2-methylpropyl diester (**S10**) as a deep blue solid. Diester (**S10**) was taken up in CH_2Cl_2 (25 mL), *N,N*-diisopropylethylamine (1.2 mL, 7.1 mmol) and chloromethyl methyl ether (539 µL, 7.09 mmol) were added, and the reaction was stirred at room temperature for 24 h while protected from light. The solution was diluted with water and extracted with CH_2Cl_2 (3 ×). The combined organic solution were dried (Na_2SO_4), filtered, and evaporated. The residue was purified by flash chromatography (0–10% MeOH/CH_2Cl_2, linear gradient, with constant 1% v/v AcOH additive) to afford 275.6 mg (55% over 2 steps) of **S11** as a dark blue solid. ^{1}H NMR (CD_3OD, 400 MHz) δ 8.32 (d, *J* = 1.1 Hz, 2H), 7.82 (s, 1H), 7.39 – 7.34 (m, 4H), 7.32 – 7.24 (m, 6H), 7.14 (s, 2H), 6.60 (s, 2H), 4.93 (AB quartet, v_A = 1978 Hz, v_B = 1962 Hz, J_{AB} = 17.0 Hz, 4H), 4.54 (s, 4H), 4.40 (s, 4H), 4.32 (s, 2H), 4.03 (s, 2H), 3.70 (t, *J* = 5.7 Hz, 4H), 3.48 (AB quartet, v_A = 1399 Hz, v_B = 1386 Hz, J_{AB} = 9.4 Hz, 4H), 3.23 (s, 6H), 3.21 (s, 6H), 3.15 (AB quartet, v_A = 1267 Hz, v_B = 1258 Hz, J_{AB} = 9.4 Hz, 4H), 2.53 (t, *J* = 6.2 Hz, 4H), 1.95 – 1.92 (m, 4H), 1.06 (s, 3H), 0.67 (s, 3H), 0.26 (s, 3H), 0.22 (s, 3H); ^{13}C NMR (MeOH-*d*$_4$, 101 MHz) δ 166.9, 165.1, 164.7, 151.7, 146.6, 140.2, 137.8, 136.1, 134.6, 133.4, 131.1, 130.9, 129.5, 128.7, 127.8, 127.5, 126.4, 125.1, 120.7, 96.3, 96.3, 69.9, 69.4, 67.8, 67.5, 55.1, 54.1, 54.0, 51.6, 39.3, 39.1, 27.0, 20.6, 16.5, 16.5, -2.6, -3.4; HRMS (ESI) calcd for $C_{61}H_{77}N_2O_{12}Si$ $[M]^+$ 1057.5240, found 1057.5245.

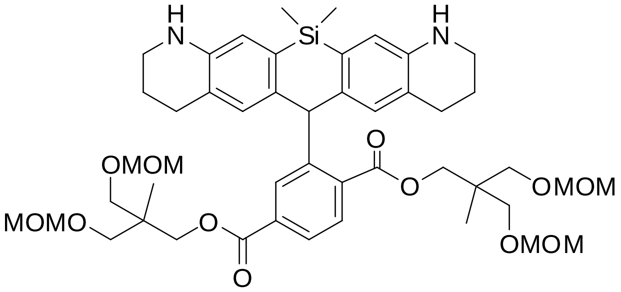

6-(2,5-Bis(3-(methoxymethoxy)-2-((methoxymethoxy)methyl)-2-methylpropoxy)carbonyl)phenyl)-13,13-dimethyl-2,3,4,8,9,10,11,13-octahydrosilino[3,2-g:5,6-g']diquinoline (4): A two-neck round-bottom flask equipped with a reflux condenser was charged with Si-rhodamine **S11** (63 mg, 0.059 mmol). The flask was sealed and evacuated/backfilled with nitrogen (3 ×). MeOH (6.6 mL) and Et_2O (2 mL) were added, followed by Pd/C (10% w/w, 30.5 mg, 29.5 µmol, 0.5 eq) and ammonium formate (55 mg, 0.88 mmol, 15 eq). Reaction mixture was stirred at 50 °C for 4 h, cooled to room temperature, and filtered through Celite. The filter-pad was washed with CH_2Cl_2, the filtrate concentrated in vacuo. Silica gel chromatography (10 g SiO_2, 5–50% EtOAc/hexanes, linear gradient) afforded 40 mg (76%) of compound **4** as a greenish-yellow gum. ^{1}H NMR ($CDCl_3$, 400 MHz) δ 7.72 – 7.62 (m, 3H), 6.85 (s, 2H), 6.70 (s, 2H), 6.37 (s, 1H), 4.62 (s, 4H), 4.53 (s, 4H), 4.46 (s, 2H), 4.14 (s, 2H), 3.56 (s, 4H), 3.40 (s, 4H), 3.34 (s, 6H), 3.31 – 3.21 (m, 4H), 3.26 (s, 6H), 2.73 – 2.51 (m, 4H), 1.93 – 1.77 (m, 4H), 1.16 (s, 3H), 0.98 (s, 3H), 0.60 (s, 3H), 0.40 (s, 3H). ^{13}C NMR ($CDCl_3$, 101 MHz) δ 168.7, 165.4, 149.9, 137.8, 132.9, 132.8, 132.6, 131.6, 130.8, 129.4, 125.4, 123.9, 119.0, 96.8, 96.7, 96.6, 71.8, 70.2, 70.0, 68.0, 67.1, 55.2, 55.1, 42.0, 39.6, 39.5, 26.9, 22.0, 17.6, 17.4, 0.54, -0.47. HRMS (ESI) calcd for $C_{47}H_{66}N_2O_{12}Si$ $[M+H]^+$ 879.4458, found 879.4460.

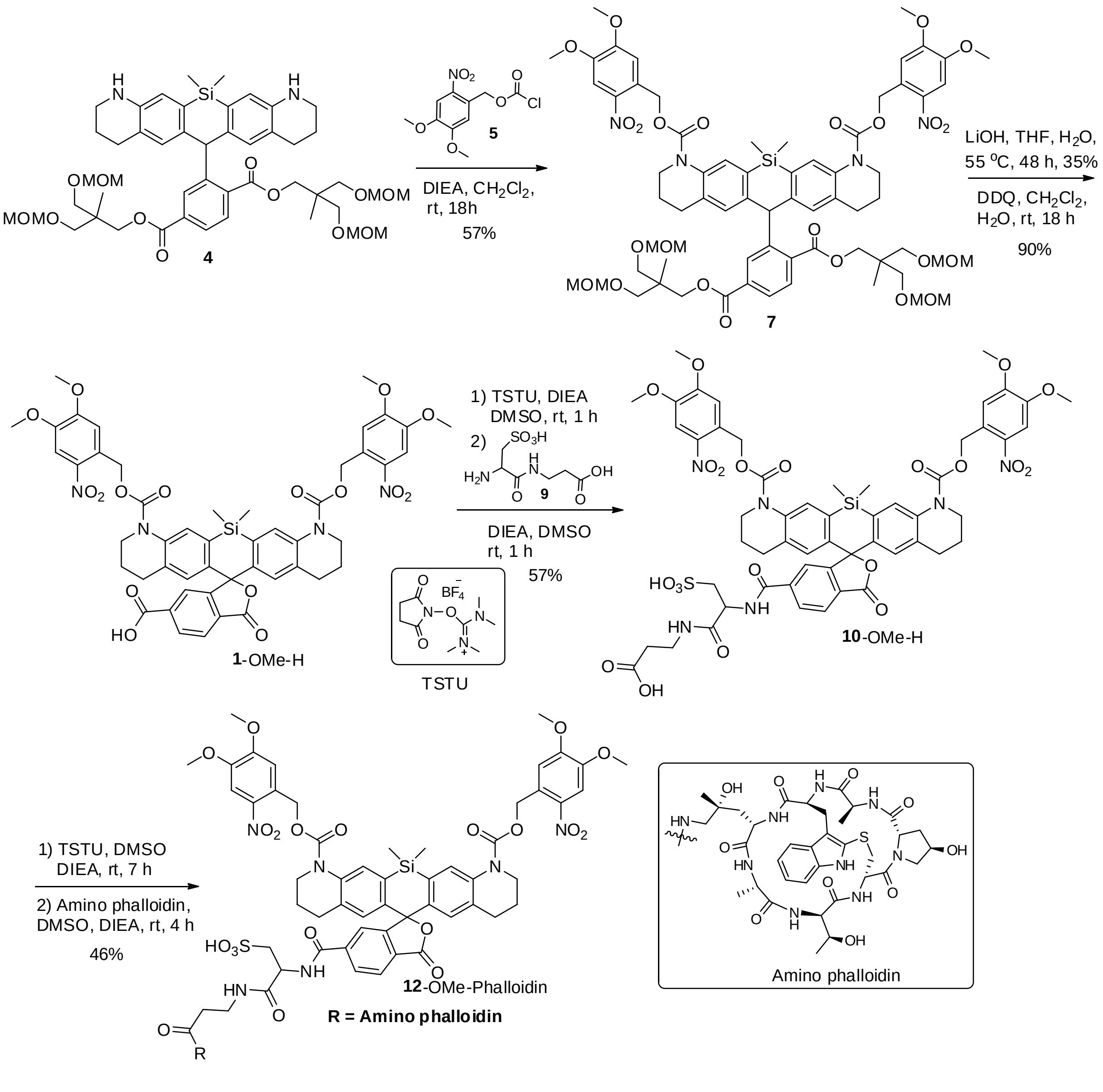

Scheme S3. NVOC2-QSiR-6-hydrophilizer-phalloidin conjugate.

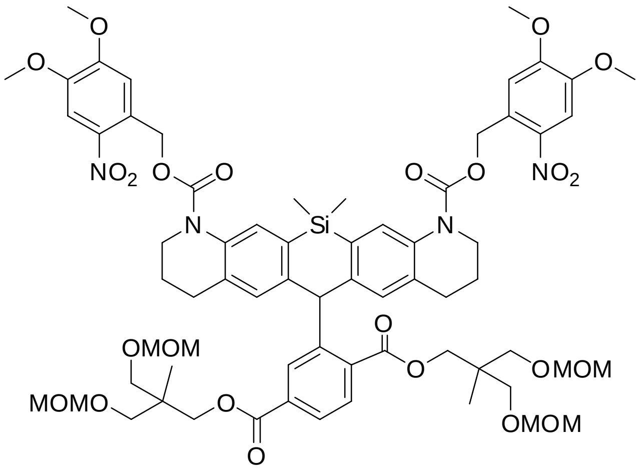

Compound 7: Compound **4** (120 mg, 0.137 mmol) was dissolved in CH_2Cl_2 (4 mL) under nitrogen. 4,5-Dimethoxy-2-nitrobenzyl chloroformate, **5** (151 mg, 0.546 mmol, 4 eq) in CH_2Cl_2 (2 mL) was added, followed by *N,N*-diisopropylethylamine (126 µL, 0.73 mmol, 5.3 eq). The reaction mixture was protected from light, stirred at room temperature for 18 h, diluted with 10% w/v aqueous citric acid and extracted with EtOAc (3×). The combined organic solutions were washed with brine, dried ($MgSO_4$), filtered, and evaporated. Flash chromatography (25 g SiO_2, 10–100% EtOAc/hexanes, linear gradient, with constant 40% v/v CH_2Cl_2 additive) provided the compound **7** (106 mg, 57%) as a light orange foam. ^1H NMR ($CDCl_3$, 400 MHz) δ 7.93 (s, 2H), 7.80 – 7.74 (m, 1H), 7.73 – 7.67 (m, 3H), 6.98 (s, 2H), 6.95 – 6.86 (m, 2H), 6.58 (s, 1H), 5.61 – 5.26 (m, 4H), 4.62 (s, 4H), 4.52 (s, 4H), 4.46 (s, 2H), 4.14 (s, 2H), 3.94 (s, 6H), 3.84 – 3.70 (m, 10H), 3.56 (s, 4H), 3.39 (s, 4H), 3.34 (s, 6H), 3.24 (s, 6H), 2.73 – 2.51 (m, 4H), 1.90 (p, *J* = 6.5 Hz, 4H), 1.15 (s, 3H), 0.97 (s, 3H), 0.60 (s, 3H), 0.32 (s, 3H); ^{13}C NMR ($CDCl_3$, 126 MHz) δ 168.3, 165.1, 154.1, 153.3, 148.5, 148.2, 143.2, 140.0, 136.0, 133.2, 132.8, 132.6, 131.4, 129.8, 129.8, 127.5, 125.9, 110.7, 108.2, 96.7, 96.6, 70.2, 69.9, 68.1, 67.3, 64.5, 56.4, 56.2, 55.2, 55.1, 47.4, 45.0, 39.7, 39.5, 27.4, 23.2, 17.6, 17.4, 1.0, -0.8; HRMS (ESI) calcd for $C_{67}H_{84}N_4O_{24}Si$ $[M+Na]^+$ 1379.5137, found 1379.5122.

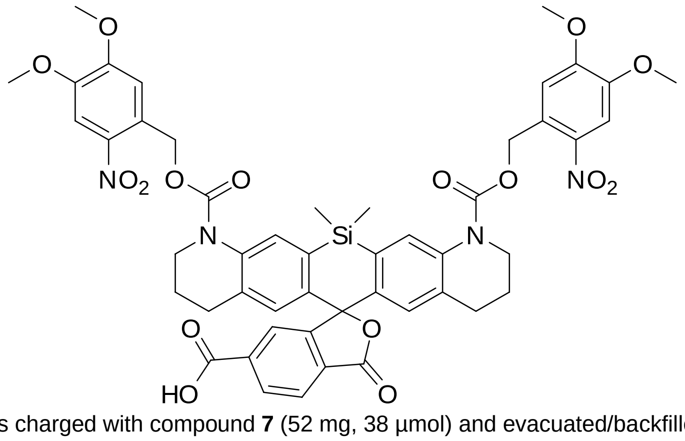

Compound 1-OMe-H**:** A vial was charged with compound **7** (52 mg, 38 µmol) and evacuated/backfilled with nitrogen (3 ×). Freshly degassed THF (3.2 mL) and 1 M aq LiOH (0.9 mL, 0.8 mmol, 20 eq) were added, and the mixture was stirred at 55 °C for 48 h (protected from light). The reaction mixture was cooled to room temperature, acidified with 1 M HCl (3 mL), diluted with water, and extracted with CH_2Cl_2 (3 ×). The organic solutions were dried ($MgSO_4$), filtered, and evaporated. The residue was purified by flash chromatography on silica gel (0–10% methanol in CH_2Cl_2) to get the di-acid target compound (13 mg, 35%) as an off-white solid which was used in the next step.The diacid was dissolved in 10:1 CH_2Cl_2/H_2O (3.3 mL); DDQ (15 mg, 0.07 mmol, 5 eq) was added, and the reaction stirred at room temperature for 18 h (protected from light). The reaction mixture was concentrated to dryness and was purified by flash chromatography on silica gel (10 g, 0–5% methanol/CH_2Cl_2, linear gradient) to afford the caged dye **1**-OMe-H as an off-white solid (4.5 mg, 90%). Analytical HPLC: >98% purity (4.6 mm x 75 mm 2.6 µm C18 column; 1 µL injection; A/B 20:80 → 100:0, A – acetonitrile, B – water + 0.05% trifluoroacetic acid; 10 min run; 1 mL/min flow. ^1H NMR (DMSO-d_6, 400 MHz) δ 8.11 – 8.01 (m, 2H), 7.93 (s, 2H), 7.71 (s, 2H), 7.60 (t, *J* = 1.0 Hz, 1H), 7.20 (s, 2H), 6.78 (s, 2H), 5.51 – 5.34 (m, 4H), 3.87 (s, 6H), 3.79 (s, 6H), 3.70 – 3.66 (m, 4H), 2.71 – 2.53 (m, 3H), 1.76 (p, *J* = 6.3 Hz, 4H), 0.31 (s, 3H), 0.19 (s, 3H). ^{13}C NMR (DMSO-d_6, 126 MHz) δ 169.2, 165.8, 155.0, 153.5, 154.0, 148.2, 140.0, 138.1, 137.7, 132.3, 130.7, 130.3, 128.8, 126.5, 125.8, 123.4, 112.5, 108.3, 88.6, 64.4, 56.2, 56.1, 44.6, 27.0, 22.5, -0.9, -1.0. HRMS (ESI) calcd for $C_{49}H_{46}N_4O_{16}Si$ $[M+H]^+$ 975.2751, found 975.2737.

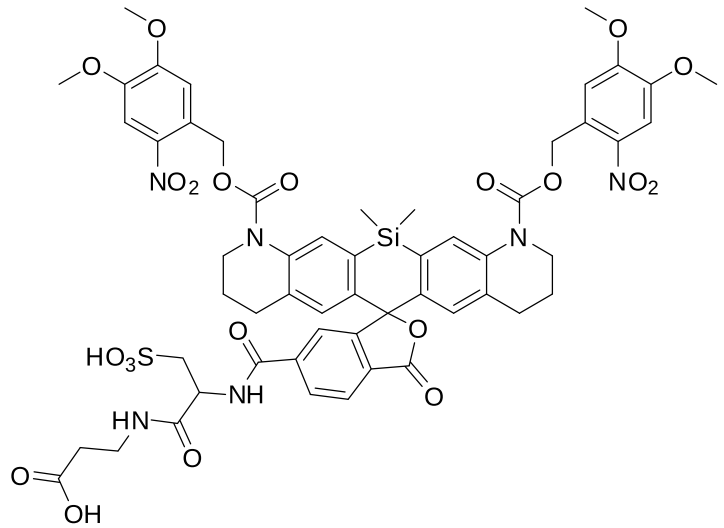

Compound 10-OMe-H**:** To a solution of acid **1**-OMe-H (2 mg, 2 µmol) in DMSO (200 µL) were added *N,N*-diisopropylethylamine (9.0 µL, 54 µmol) and TSTU (1.15 mg, 3.84 µmol). The reaction mixture was protected from light and stirred at room temperature for 1 h. To the NHS ester formed in the reaction mixture, a hydrophylizer linker, $H_2NCH(CH_2SO_3H)CONH(CH_2)_2CO_2H$ (1.95 mg, 7.80 µmol), was added, and the reaction mixture kept for 15 min in the ultrasonic bath at room temperature (shaken from time to time). Water (20 µL) was added and the reaction mixture was stirred at room temperature for another 45 min. After complete conversion (HPLC control), the reaction was "quenched" by addition of acetic acid (3.6 µL, 62 µmol), frozen in a bath with acetone – dry ice mixture, dried by lyophilization and purified by prep. HPLC (Interchim, gradient A:B 70:30 → 100:0 over 30 min, A – acetonitrile, B – water + 0.1% trifluoroacetic acid) to obtain compound **10**-OMe-H (1.4 mg 57%) as a colourless solid. 95% HPLC area (column 4.6 mm x 75 mm, 2.6 µm Kinetex C18 100; A/B 20:80 → 100:0, A – acetonitrile, B – water + 0.05% trifluoroacetic acid; 10 min run; 1 mL/min flow). HRMS (ESI) calcd for $C_{55}H_{56}N_6O_{21}SSi$ $[M-H]^+$ 1195.2916, found 1195.2945.

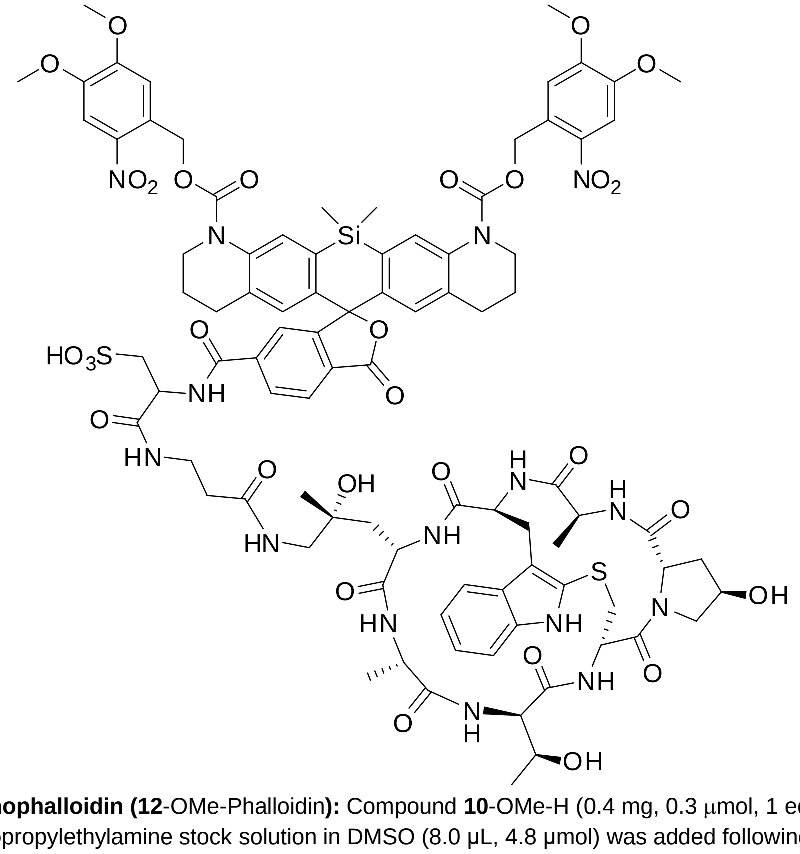

2 x NVOC-QSiR-6-h-aminophalloidin (12-OMe-Phalloidin**):** Compound **10**-OMe-H (0.4 mg, 0.3 µmol, 1 eq) was dissolved in DMSO (70 µL) and 10% *N,N*-diisopropylethylamine stock solution in DMSO (8.0 µL, 4.8 µmol) was added following by TSTU (0.4 mg, 1.2 µmol; 3.3 µL of a stock solution containing 3.0 mg TSTU in 25 µL DMSO). The reaction mixture was protected from light and stirred at room temperature for 1 h. HPLC displayed ca. 90% conversion to the corresponding NHS ester. Then aminophalloidin (1.0 mg, 1.3 µmol) in DMSO (25 µL) and 10% *N,N*-diisopropylethylamine stock solution in DMSO (4.0 µL, 2.4 µmol) were added. The reaction mixture was stirred at room temperature for 4 h. After completion of the reaction (HPLC control), the reaction mixture was frozen,

lyophilized and purified by reverse phase HPLC (A/B 0:100 → 100:0 over 20 min, A – acetonitrile, B – water + 0.1% trifluoroacetic) to afford 0.3 mg (46%) of target compound **12**-OMe-Phalloidin. Analytical HPLC: 95% HPLC area (4.6 mm x 75 mm, 2.6 µm, C18 column; 1 µL injection; A:B 20:80 → 100:0, A – acetonitrile, B – water + 0.05% trifluoroacetic acid; 10 min run; 1 mL/min flow). HRMS (ESI) calcd for $C_{90}H_{103}N_{15}O_{30}S_2Si$ $[M-H]^+$ 1964.6123, found 1964.6141.

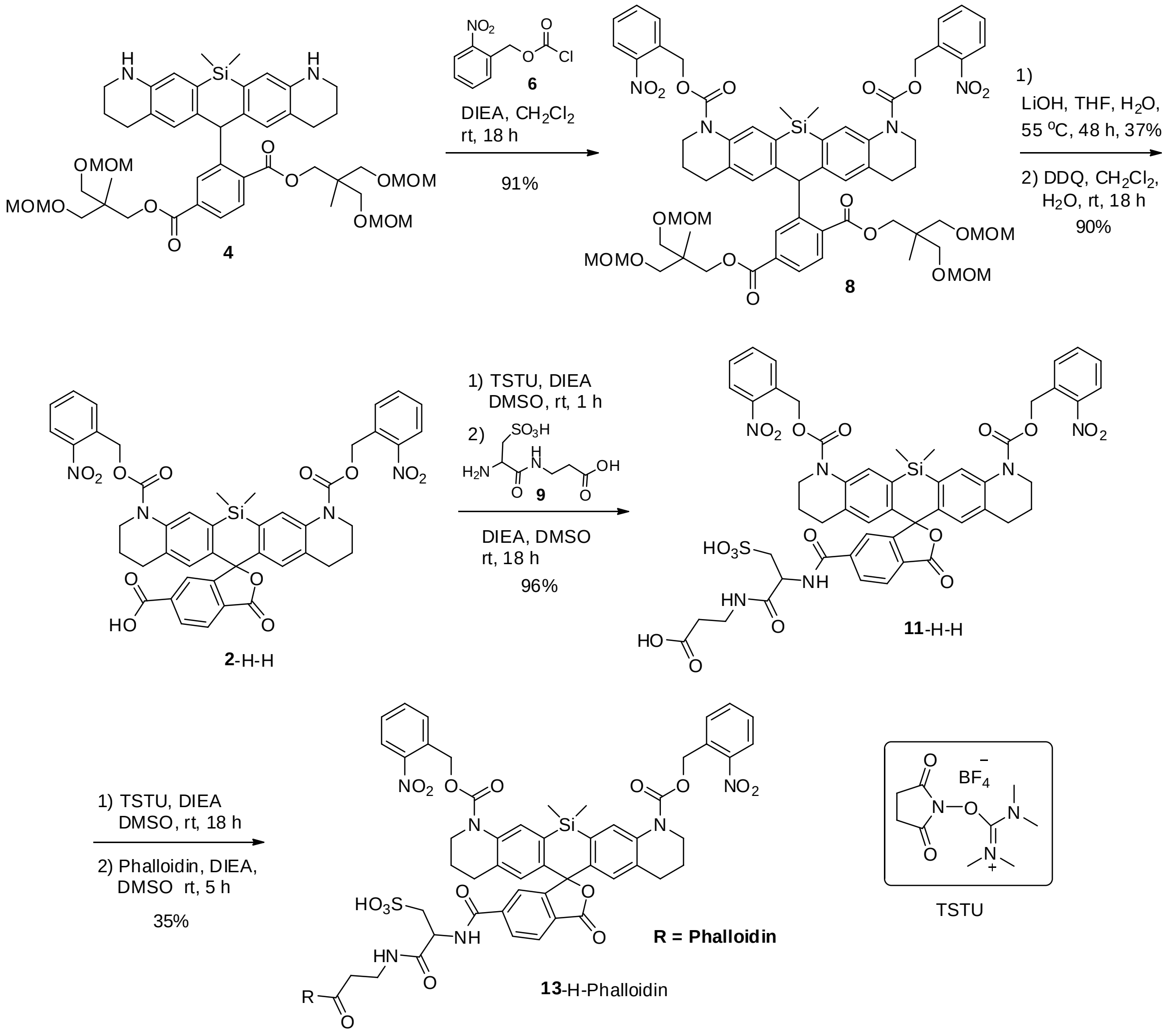

Scheme S4. Synthesis of phalloidin conjugate of dye **13** decorated with a hydrophilizer linker

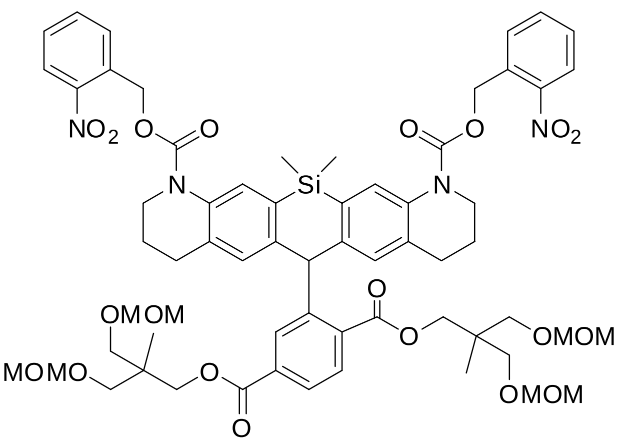

Bis[(2-nitrophenyl)methyl] 6-[2,5-bis({3-(methoxymethoxy)-2-[(methoxymethoxy)methyl]-2-methylpropoxy}carbonyl)phenyl]-13,13-dimethyl-3,4,8,9,10,13-hexahydrosilino[3,2-*g*:5,6-*g'*]diquinoline-1,11(2*H*,6*H*)-dicarboxylate (8): Compound **4** (18 mg, 20.5

µmol) was dissolved in CH_2Cl_2 (1.0 mL) under nitrogen. The freshly prepared solution of 2-nitrobenzyl chloroformate[5] (**6**) (17.6 mg, 81.6 µmol, 4 eq) in CH_2Cl_2 (0.5 mL) was added, followed by *N,N*-diisopropylethylamine (22 µL, 120 µmol, 6 eq). The reaction mixture was stirred at room temperature for 18 h (protected from light), diluted with 10% w/v aqueous citric acid and extracted with EtOAc (3 ×). The combined organic solutions were washed with brine, dried (Na_2SO_4), filtered, and evaporated. The crude product was purified by flash chromatography on silica gel (25 g; 10–100% EtOAc/hexanes, linear gradient, with constant 40% v/v CH_2Cl_2 additive) provided **8** (23 mg, 91%) as an off-white solid. ^{1}H NMR ($CDCl_3$, 400 MHz) δ 8.09 (dd, *J* = 8.2, 1.3 Hz, 2H), 7.93 (s, 2H), 7.82 – 7.72 (m, 2H), 7.70 (dd, *J* = 8.1, 1.7 Hz, 1H), 7.64 (td, *J* = 7.6, 1.3 Hz, 2H), 7.57 (dd, *J* = 7.9, 1.5 Hz, 2H), 7.49 – 7-45 (m, 2H), 7.01 (s, 2H), 6.61 (s, 1H), 5.62 (AB quartet, v_A = 2255.85 Hz, v_B = 2241.63 Hz, J_{AB} = 14.8.0 Hz, 4H), 4.62 (s, 4H), 4.51 (s, 4H), 4.47 (s, 2H), 4.14 (s, 2H), 3.83 – 371(m, 4H), 3.57 (s, 4H), 3.39 (s, 4H), 3.34 (s, 6H), 3.24 (s, 6H), 2.73 – 257 (m, 4H), 1.92 – 186 (m, 4H), 1.16 (s, 3H), 0.97 (s, 3H), 0.60 (s, 3H), 0.34 (s, 3H); ^{13}C NMR ($CDCl_3$, 101 MHz) δ 168.3, 165.1, 154.1, 148.6, 147.5, 143.3, 136.0, 133.8, 133.2, 132.8, 132.7, 132.6, 131.6, 131.3, 130.0, 129.8, 129.1, 128.6, 125.8, 125.0, 96.7, 96.6, 70.2, 69.9, 68.1, 67.3, 64.4, 55.2, 55.1, 47.5, 45.1, 39.7, 39.5, 27.4, 23.2, 17.6, 17.4, 0.31, -0.59; HRMS (ESI) calcd for $C_{63}H_{76}N_4O_{20}Si$ $[M+Na]^+$ 1259.4714, found 1259.4713.

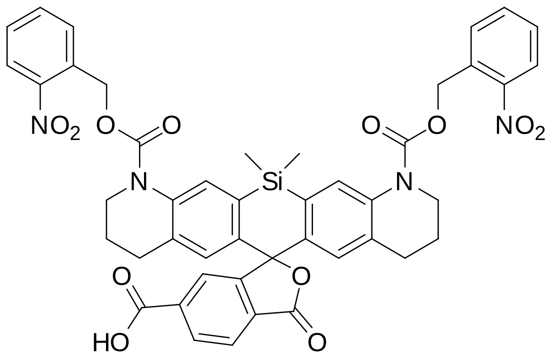

2 x 2-NBnOC-QSiR-6-Carboxylic acid (2-H-H): A vial was charged with **8** (23.4 mg, 18.9 µmol), sealed, and evacuated/backfilled with nitrogen (3 ×). Freshly degassed THF (1.5 mL) and 1 M aq LiOH solution (0.40 mL, 0.38 mmol, 20 eq) were added, and the mixture was stirred at 55 °C for 48 h (protected from light). The reaction mixture was cooled to room temperature, acidified with 1 M HCl (1 mL), diluted with water, and extracted with CH_2Cl_2 (3 ×). The combined organic solutions were dried (Na_2SO_4), filtered, and evaporated. The residue was purified by flash chromatography on silica gel (0–10% methanol in CH_2Cl_2) to get the desired leuco-diacid **2**-H-H (6.0 mg, 37%) as an off-white solid which was used in the next step.The di-acid (5.0 mg, 5.8 µmol, 1 eq) was dissolved in 10:1 CH_2Cl_2/H_2O (1.1 mL); DDQ (6.6 mg, 29 µmol, 5 eq) was added, and the reaction mixture stirred at room temperature for 18 h (protected from light). The mixture was concentrated in vacuo and was purified by flash chromatography on silica gel (10 g; 0–5% methanol/CH_2Cl_2, linear gradient) to afford diacid **2**-H-H as an off-white solid (“closed” form, 4.5 mg, 90%). Analytical HPLC: >98% HPLC area (4.6 mm x 75 mm, 2.6 µm, C18 column; A:B 20:80 → 100:0, A – acetonitrile, B – water + 0.05% trifluoroacetic acid; 10 min run; 1 mL/min flow; ^{1}H NMR (DMSO-*d6*, 400 MHz) δ 8.11 (dd, *J* = 8.1, 1.3 Hz, 2H), 8.08 – 8.00 (m, 2H), 7.98 (s, 2H), 7.79 – 7.75 (m, 2H), 7.71 – 7.57 (m, 5H), 6.83 (s, 2H), 5.49 (AB quartet, v_A = 2199.6 Hz, v_B = 2193.8 Hz, J_{AB} = 14 Hz, 4H), 3.83 – 3.57 (m, 4H), 2.73 – 2.53 (m, 4H), 1.77 (m, 4H), 0.37 (s, 3H), 0.24 (s, 3H). ^{13}C NMR (DMSO-*d6*, 101 MHz) δ 169.4, 165.8, 155.5, 153.4, 147.5, 138.2, 137.6, 134.1, 132.4, 131.4, 130.7, 130.5, 130.2, 129.9, 129.5, 128.8, 126.4, 125.6, 124.9, 123.2, 114.3, 88.5, 64.1, 44.7, 26.9, 22.4, -0.5, -0.7. HRMS (ESI) calcd for $C_{45}H_{38}N_4O_{12}Si$ $[M+H]^+$ 855.2328, found 855.2318.

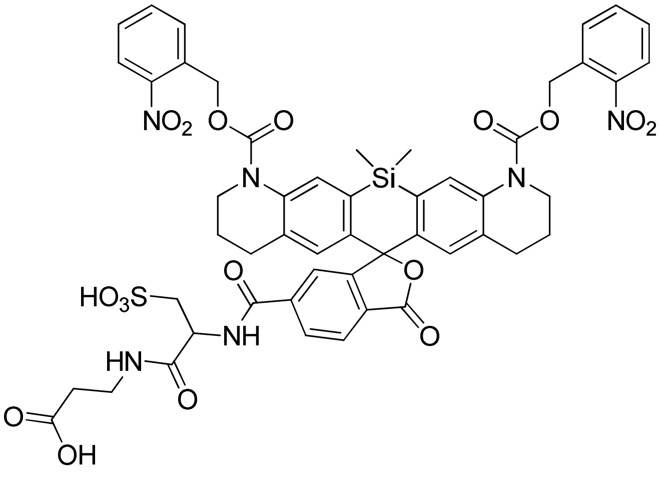

Compound 11-H-H: To a solution of acid **2**-H-H (4.1 mg, 4.8 µmol) and TSTU (2.9 mg, 9.6 µmol, 2 eq) in anhydrous DMSO (300 µL) was added *N,N*-diisopropylethylamine (21.9 µL, 125 µmol, 5 eq). The reaction mixture was protected from light and stirred at room

temperature for 1 h (monitored by HPLC). To NHS ester formed in the reaction mixture, hydrophilizer (**9**)[6] (4.0 mg. 14.4 µmol) was added and the reaction mixture was sonicated in the ultrasonic bath at room temperature (shaken from time to time) for 15 min. Water (20 µL) was added and the reaction mixture was further stirred at room temperature for 18 h (monitored by HPLC). After completion of the reaction (HPLC comtrol), 9 µL of acetic acid were added. The reaction mixture was frozen and dried by lyophilization. The residue was separated by prep. HPLC; A:B 20:80 → 100:0 over 25 min (A – acetonitrile, B – water + 0.1% trifluoroacetic acid) to afford acid **11**-H-H (5 mg, 96%) as a solid. Analytical HPLC: >98% HPLC area, t_R = 6.1 min (column 4.6 mm x 75 mm, Kinetex 2.6 µm C18 100; 20 – 80% CH_3CN/H_2O, linear gradient, with constant 0.05% v/v TFA additive; 10 min run; 1 mL/min flow; HRMS (ESI) calcd for $C_{51}H_{48}N_6O_{17}SSi$ $[M-H]^+$ 1075.2493, found 1075.2505.

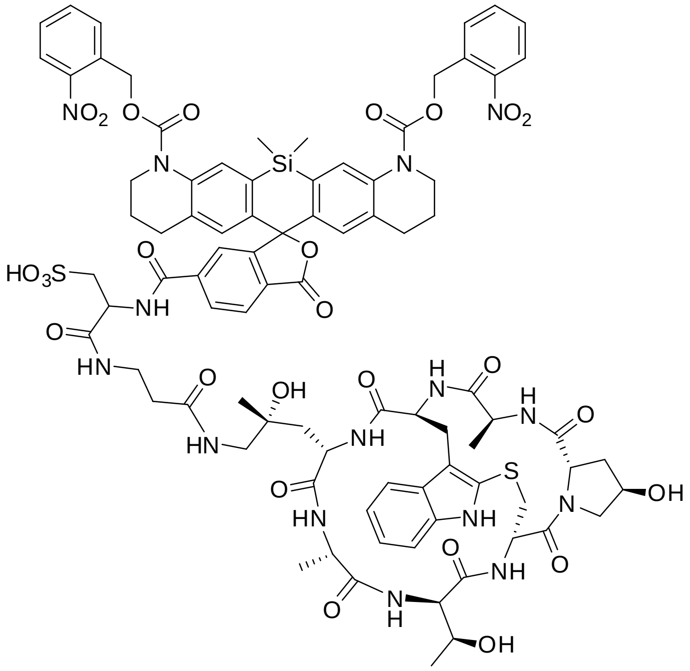

Compound 13-H-Phalloidin **(R = *N*-hydroxysuccinimidyloxy / NH-phalloidin):** Acid **11**-H-H (1.0 mg, 0.93 µmol, 1 eq) was combined with TSTU (0.56 mg, 1.86 µmol, 2 eq) and *N,N*-diisopropylethylamine (1.6 µL, 9.3 µmol, 10 eq), in anhydrous DMSO (300 µL), and the reaction mixture stirred at room temperature for 1 h (monitored by HPLC) and protected from light. HPLC analysis showed the degree of conversion to compound **11**-H-Su (t_R = 6.4 min) of ca. 90%; t_R = 6.4 min (for HPLC conditions, see compound **11**-H-H). $C_{55}H_{51}N_7O_{19}SSi$, M = 1173.3, $[M-H]^+$ found 1172.3. Aminophalloidin tosylate (1.0 mg, 1.0 µmol) was added, and the reaction mixture was stirred for additional 5 h at room temperature. The reaction mixture was frozen in a bath with dry ice and acetone and dried by lyophilization. The residue was purified by reverse phase HPLC (A:B = 0:100 → 100:0 over 20 min, A – acetonitrile, B – water + 0.1% trifluoroacetic acid) to afford **13**-H-Phalloidin (600 µg, 35%) as a solid. Analytical HPLC: 95% area (250 x 4.6 mm, 10 µm, C18 column; Solvent A: water + 0.1% TFA; solvent B: ACN + 0.1% TFA. 0-3 min: 40% B, 3–15 min: 40–100% B; 20 min run; 1.2 mL/min flow); HRMS (ESI) calcd for $C_{86}H_{95}N_{15}O_{26}S_2Si$ $[M-H]^+$ 1844.5711, found 1844.5640.

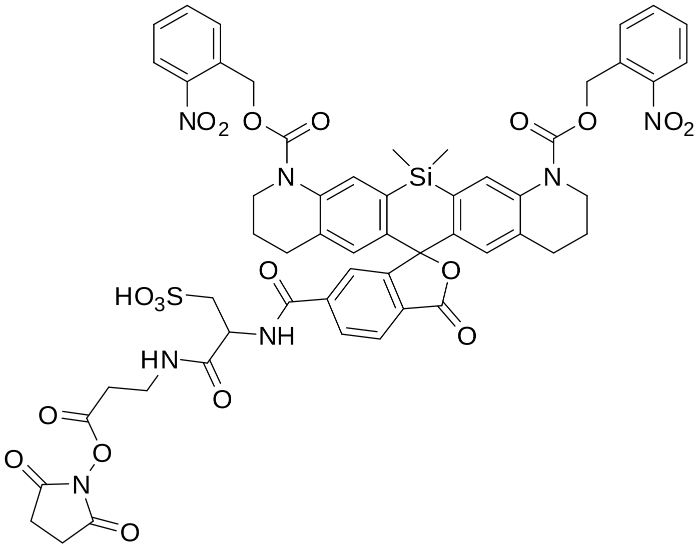

Compound 11-H-Su**:** For conjugation with antibodies, compound **11**-H-Su was generated with ca. 90% conversion from 0.29 mg (270 nmol) of compound **11**-H-H in dry DMSO (50 μL) in the course of addition of 3.6 μL (2.1 umol) of 10% $i\mathrm{Pr_2Net}$ in DMSO and 1.0 μL of the stock solution of 3.0 mg TSTU in 25 μL DMSO; reaction time 1 h.

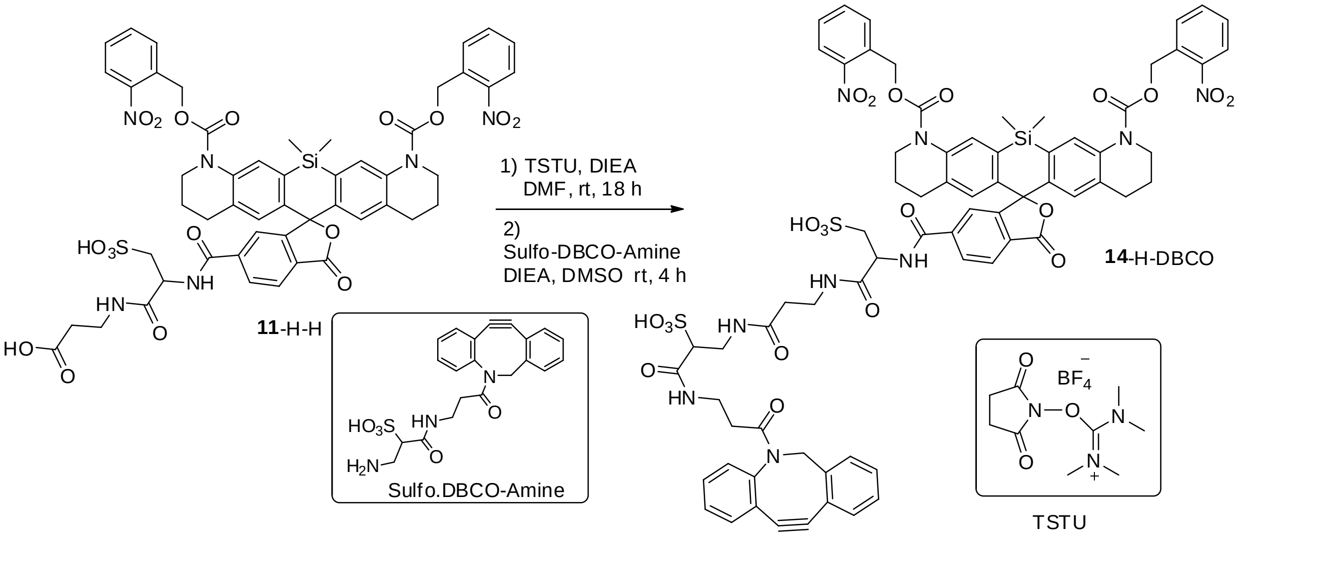

Scheme S5. Synthesis of the sulfo-DBCO-amine conjugate of dye **14**-H-DBCO for "click chemistry".

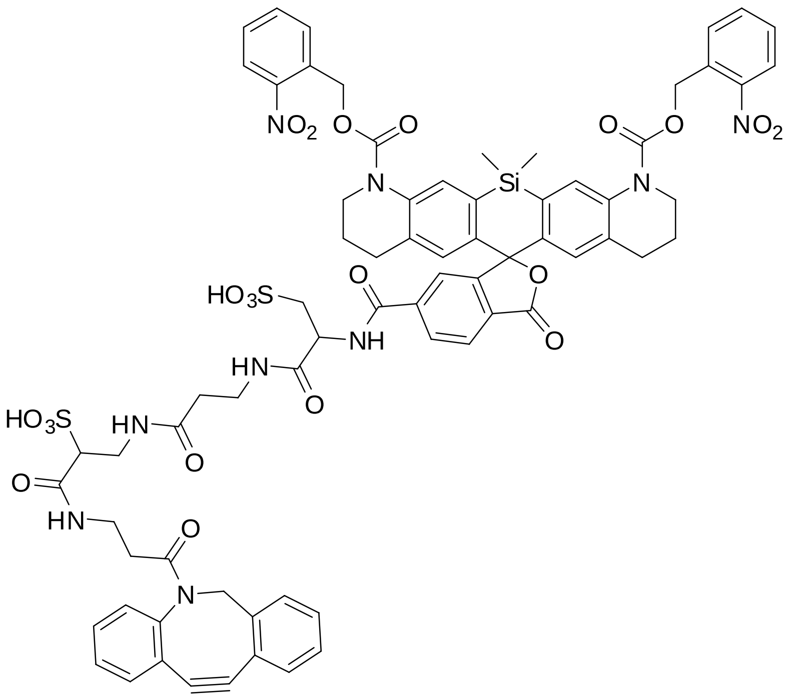

2 x 2-NBnOC -QSiR-6-hydrophylizer-linker-Sulfo-DBCO (14-H-DBCO**):** NHS ester of acid **11**-H-H (2.0 mg, 1.7 µmol) was dissolved in anhydrous DMSO (300 µL), then *N,N*-diisopropylethylamine (4.2 µL, 23.8 µmol) and DBCO-amine (2.2 mg, 5.1 µmol) were added. The reaction mixture was stirred at room temperature for 4 h (protected from light), and the course of the reaction monitored by HPLC. The reaction mixture was frozen, dried by lyophilisation, and the residue purified by prep. HPLC (A/B 50:50 → 90:10 over 20 min, A – acetonitrile, B – water + 20mM TEA-FA, pH-6.6) to afford compound **14**-H-DBCO (1.0 mg, 45%) as a solid. Analytical HPLC: >95% purity (4.6 mm x 75 mm 2.6 µm C18 column; 1 µL injection; 20–80 → 100:0 CH_3CN/H_2O TEA-FA 20 mM pH~4; 10 min run; 1 mL/min flow; HRMS (ESI) calcd for $C_{72}H_{67}N_9O_{21}S_2Si$ $[M-H]^+$ 1484.3589, found 1484.3609.

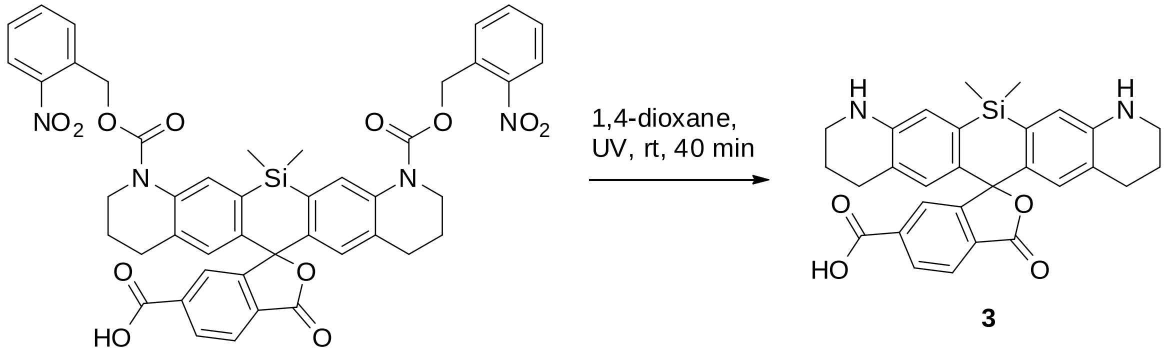

2-H-H

SiR (3): Compound **2**-H-H (3.0 mg, 3.5 µmol, 1 eq) was dissolved in 1,4-dioxane (3 mL). The solution in a quartz cuvette was irradiated with 365 nm UV lamp and stirred for 40 min. The reaction mixture was concentrated and the residue was subjected to prep. HPLC (A:B 0:100 → 100:0 over 20 min, A – acetonitrile, B – water + 0.1% trifluoroacetic acid) to afford SiRhodamine (**3**) trifluoroacetate as a deep blue solid (700 µg, 40%). Analytical HPLC: >95% HPLC area (75 mm x 4.6 mm, 2.6 µm C18 column; Solvent A: ACN; solvent B: water + 0.05% TFA. A/B 0:15 → 100:0 over 10 min run; 1 mL/min flow; ^{1}H NMR (CD_3OD, 400 MHz) δ 8.34 – 8.22 (m, 2H), 7.78 (dd, *J* = 1.6, 0.6 Hz, 1H), 6.98 (s, 2H), 6.56 (s, 2H), 3.48 – 3.44 (m, 4H), 2.48 (t, *J* = 6.2 Hz, 4H), 1.89 – 1.75 (m, 4H), 0.51 (s, 3H), 0.45 (s, 3H). ^{19}F NMR (CD_3OD, 376 MHz) δ -77.2 (s); HRMS (ESI) calcd for $C_{29}H_{28}N_2O_4Si$ $[M+H]^+$ 497.1891, found 497.1889.

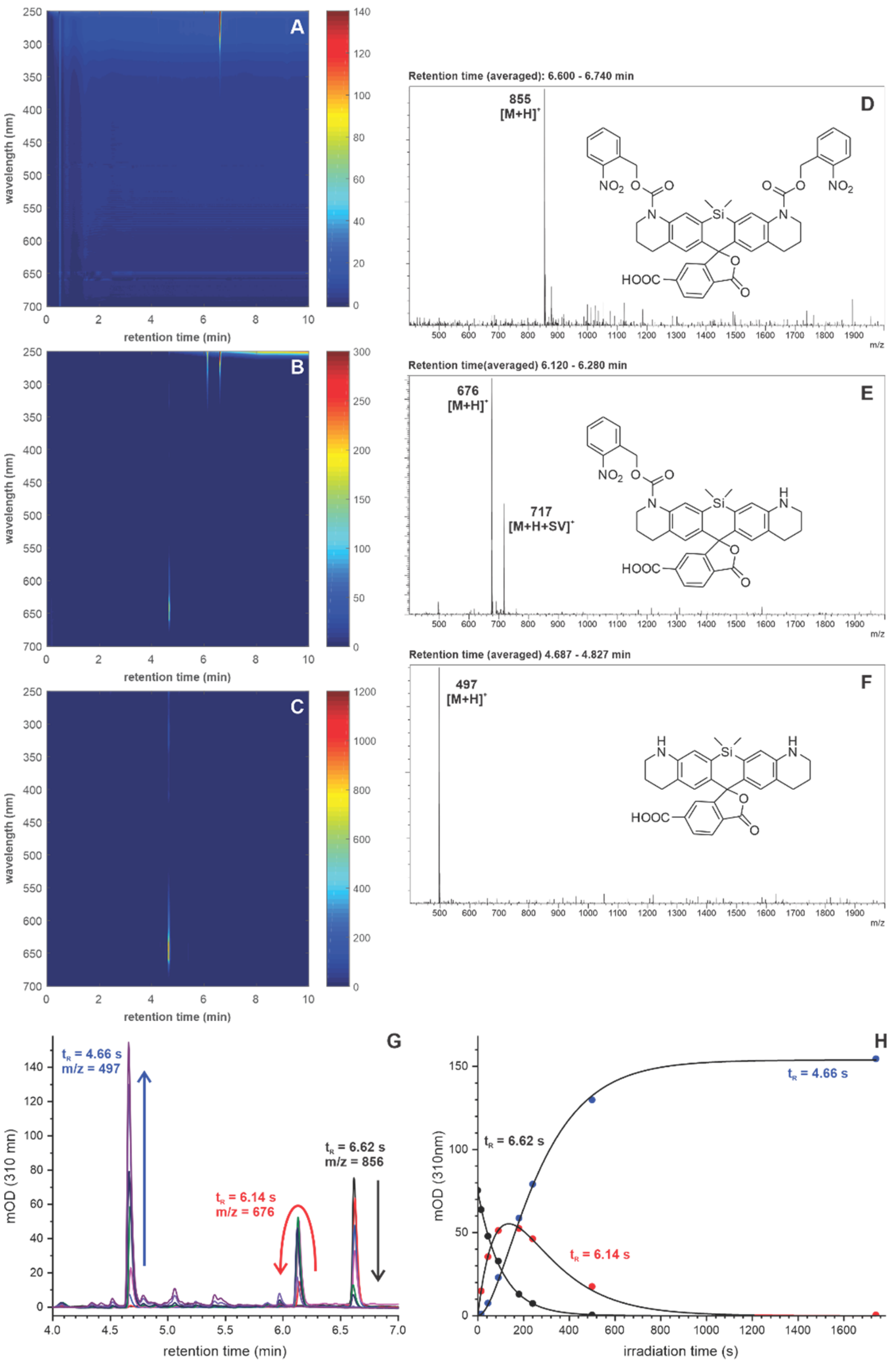

Figure S1. “Uncaging” (photoactivation) experiments with compound **2**-H-H, in a 1:1 mixture of acetonitrile and phosphate buffer (100 mM, pH = 7). Small aliquots of the irradiated solution were extracted at specific times and analyzed by LC-MS. (A, B,C) 3D absorption maps in mOD of the starting solution (A), a partially photoactivated solution (B), and the solution obtained at the end of the experiment (C). (D, E, F) Mass spectra of the three main peaks observed in the chromatogram of (B), when the reaction was incomplete. (G) Chromatograms at different irradiation times; the wavelength for detection was selected arbitrarily, to obtain a reasonable absorption for all reactants. (H) Temporal evolution of the intensities of the three main peaks. The curves correspond to a global fit based on two consecutive reactions.

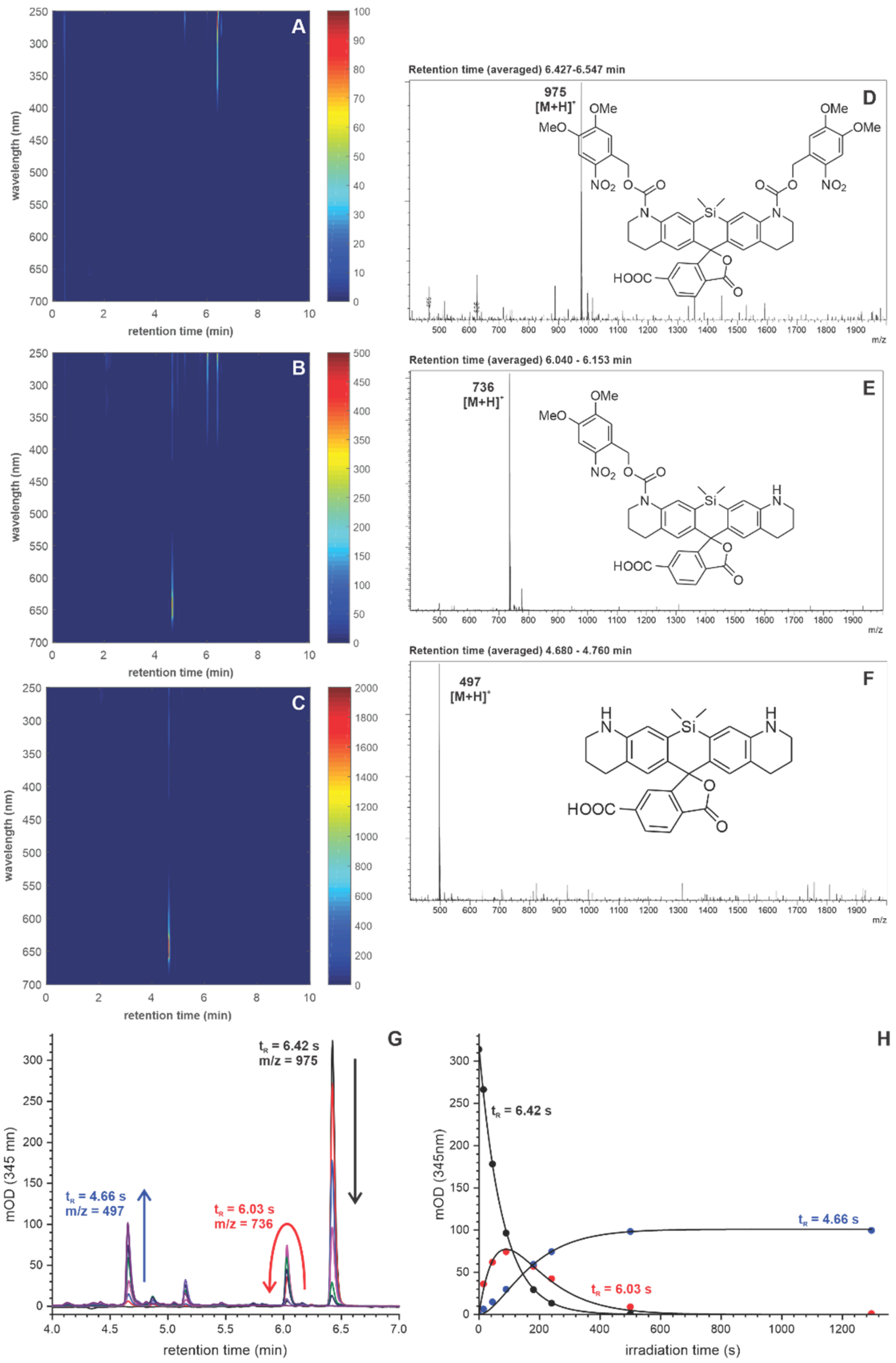

Figure S2. "Uncaging" (photoactication) experiments with compound **1**-OMe-H, in a 1:1 mixture of acetonitrile and phosphate buffer (100 mM, pH = 7). Small aliquots of the irradiated solution was extracted at specific times and analyzed by LC-MS. (A, B,C) 3D absorption maps in mOD of the starting solution (A), a partially photoactivated solution (B), and the solution obtained at the end of the experiment (C). (D, E, F) Mass spectra of the three main peaks observed in the chromatogram of (B), when the reaction was incomplete. (G) Chromatograms at different irradiation times; the wavelength for detection was selected arbitrarily, to obtain a reasonable absorption for all reactants. (H) Temporal evolution of the intensities of the three main peaks. The curves correspond to a global fit involving two consecutive reactions scheme.

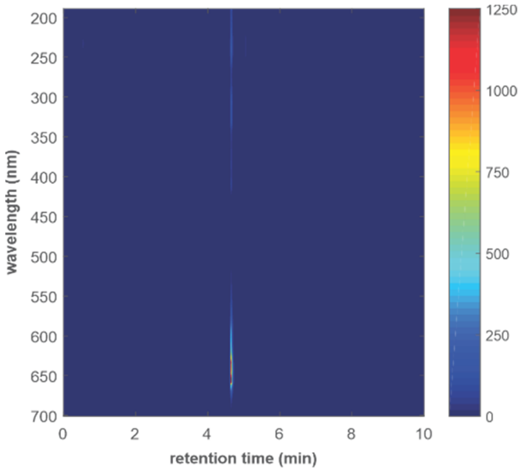

Figure S3. LC-MS (3D absorption map in mOD) of model compound **3** (free dye formed upon full photo-activation) recorded under the same conditions, as for the irradiated solutions of the two caged compounds (**2**-H-H and **1**-OMe-H).

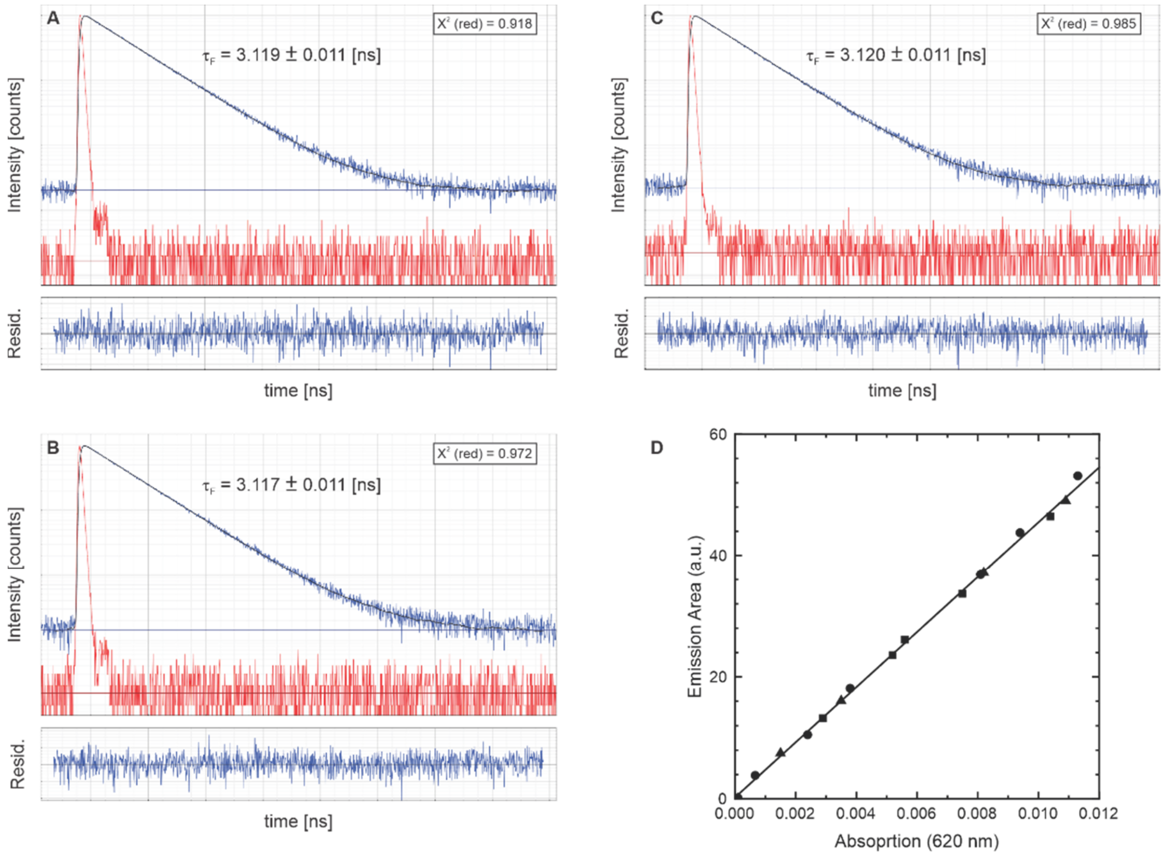

Figure S4. Fluorescence decays of the irradiated/partially "uncaged" compounds **1**-OMe-H (**A**) and **2**-H-H (**B**) in solutions and a diluted solution of compound **3** (**C**), in a 1:1 mixture of acetonitrile and phosphate buffer (100 mM, pH = 7). (D) The total integrated emission vs. the absorption at the excitation wavelength (620 nm) is shown for compounds **1**-OMe-H (triangles), **2**-H-H (circles) and **3** (squares), in the same solvent. For the "caged" (masked) compounds, starting solutions were stepwise irradiated, while the concentration for compound **3** was increased by additions of a stock solution. An emission quantum yield of 0,60 was calculated, using Nile Blue in ethanol as a reference dye (Φ_{Fluo} = 0.27)[7].

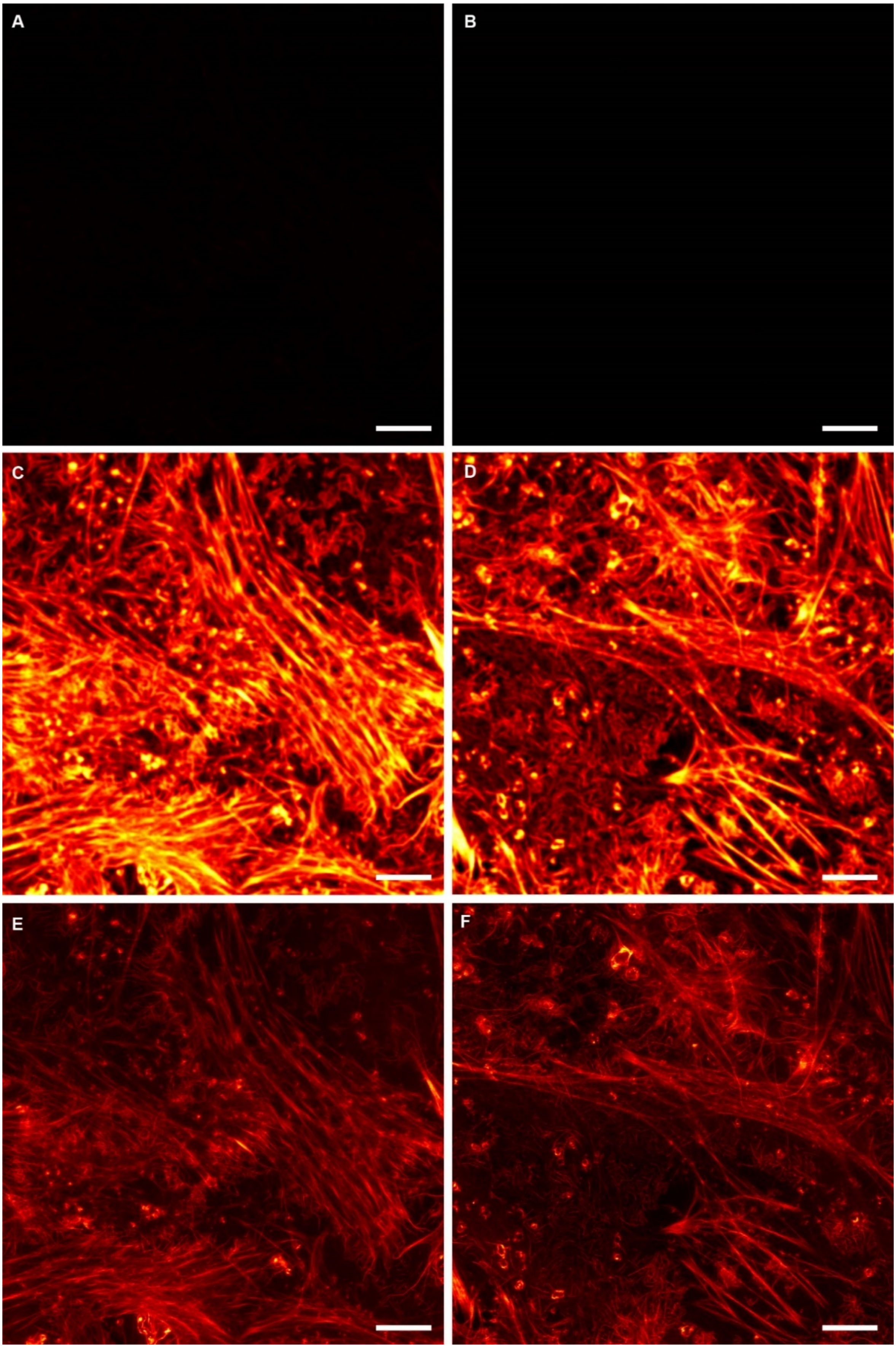

Figure S5. Confocal (**A** - **D**) and STED (**E**, **F**) images of fixed Hela labelled with compounds **12**-OMe-Phalloidin (**A**, **C**, **E**) and **13**-H-Phalloidin (**B**, **D**, **F**) before UV activation (**A**, **B**), after UV activation (**C** - **F**). Confocal images share the same colormap. Scale-bars: 5 µm

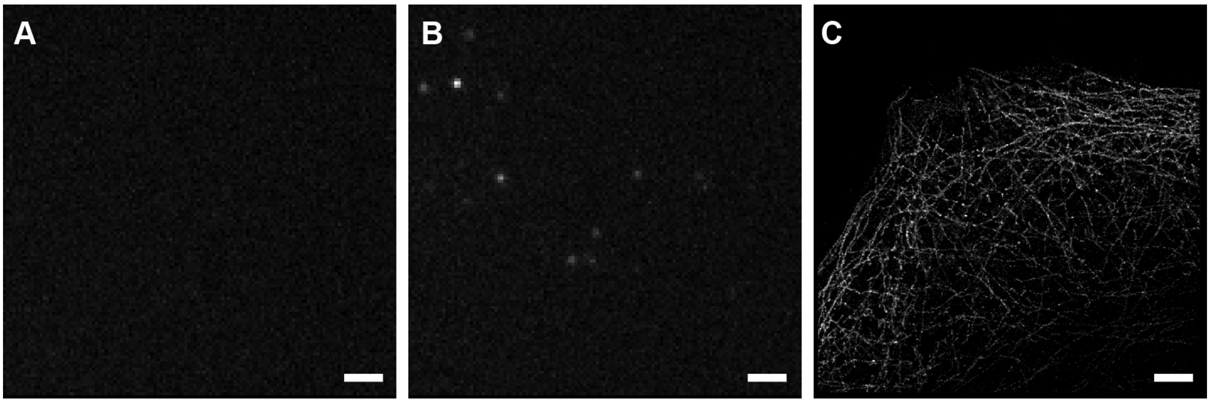

Figure S6. TIRF images of a PFA fixed Hela cells immunostained with primary antibody again alpha-tubulin and secondary antibodies with compound **14**-H-DBCO without **A** and with **B** 405 nm UV activation. **C**: PALM image of the same area. Scale-bars: 2 µm

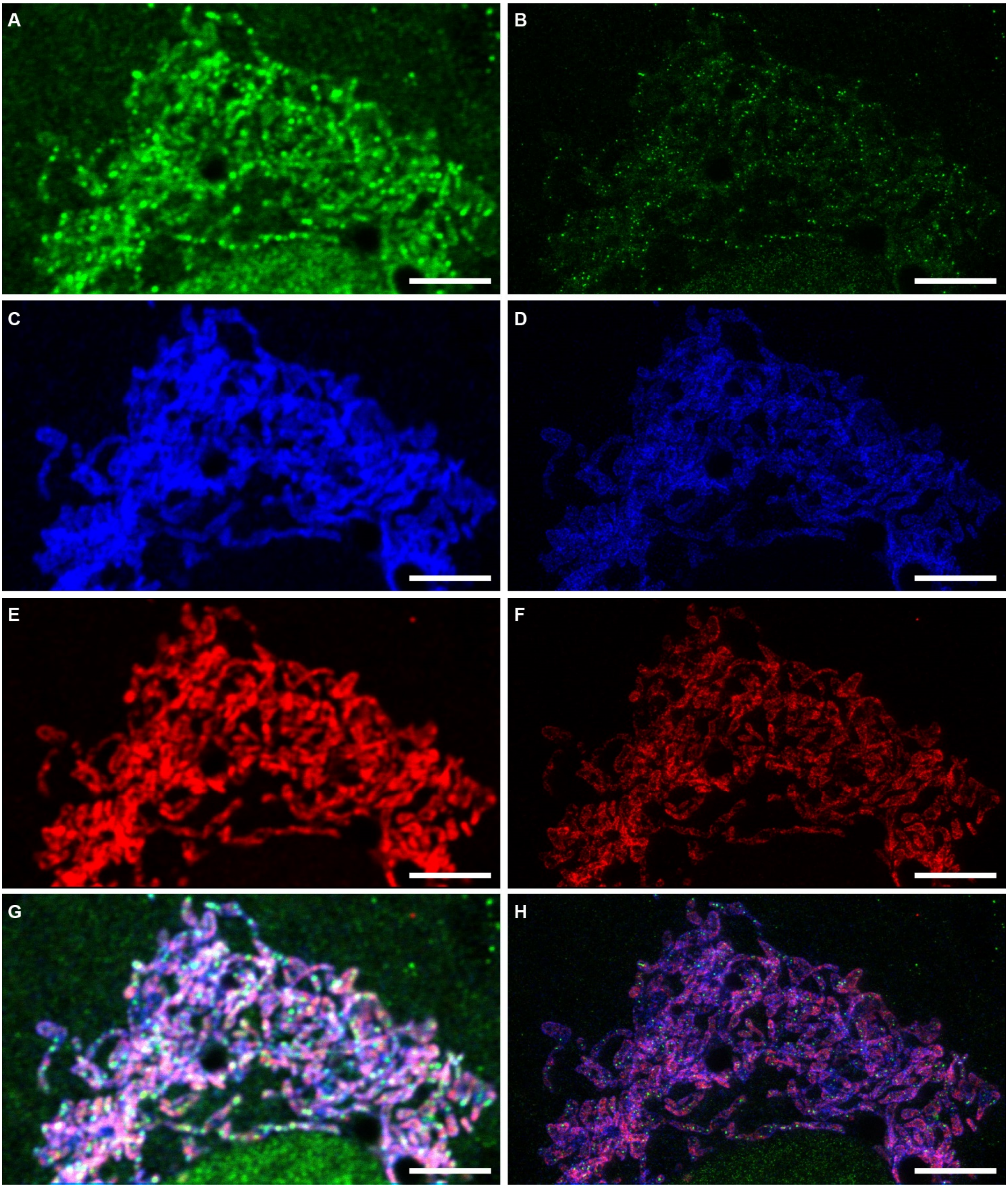

Figure S7 Confocal (**A**, **C**, **E**, **G**) and STED (**B**, **D**, **F**, **H**) images of fixed U2OS cells labeled with primary antibody against DNA (**A**, **B**, **G**/**H**: green), TOM 20 (**C**, **D**, **G**/**H**: blue) and ATP-Synthase B (**E**, **F**, **G**/**H**: red) conjugated with compound **14**-H-DBCO and secondary antibodies conjugated with fluorescent dyes STAR 635P (green) and Alexa 594 (blue). Scale-bars: 5 µm

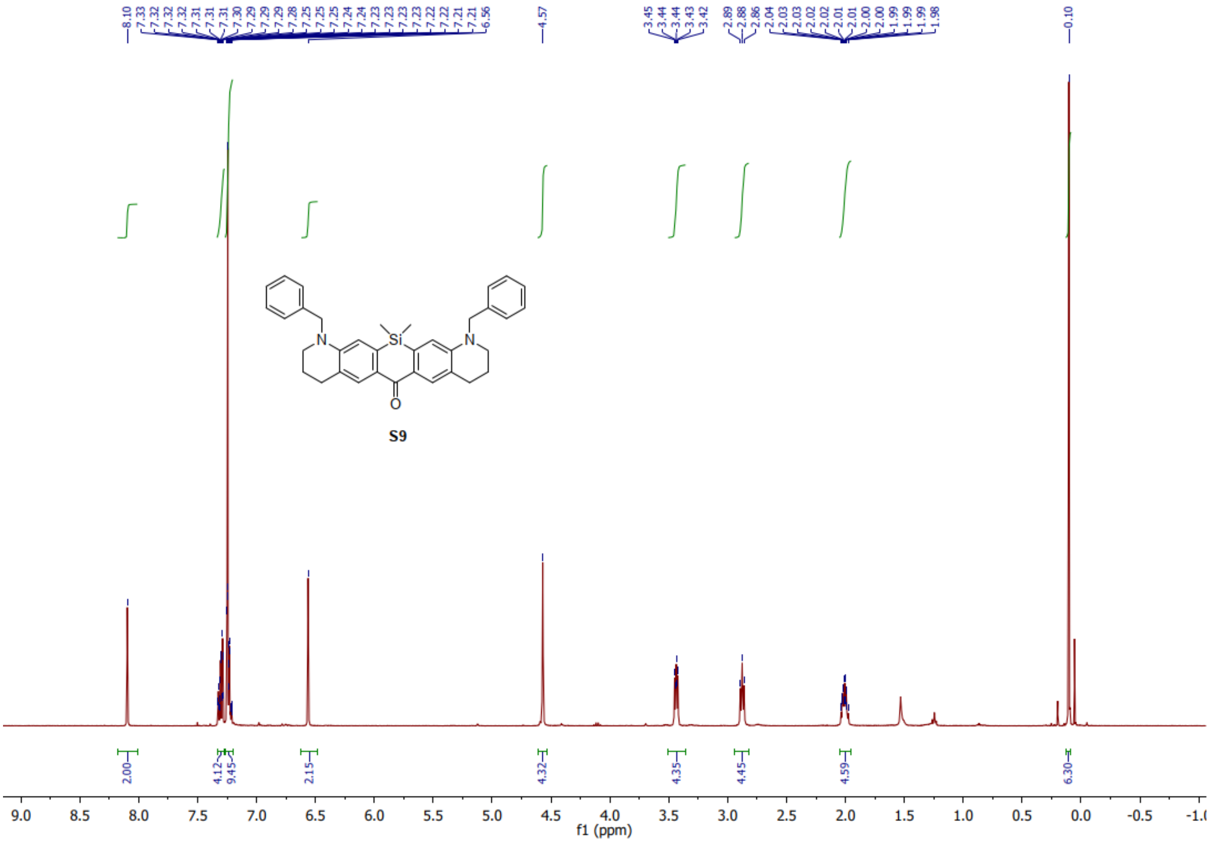

^1H NMR ($CDCl_3$, 400 MHz) of compound **S9**

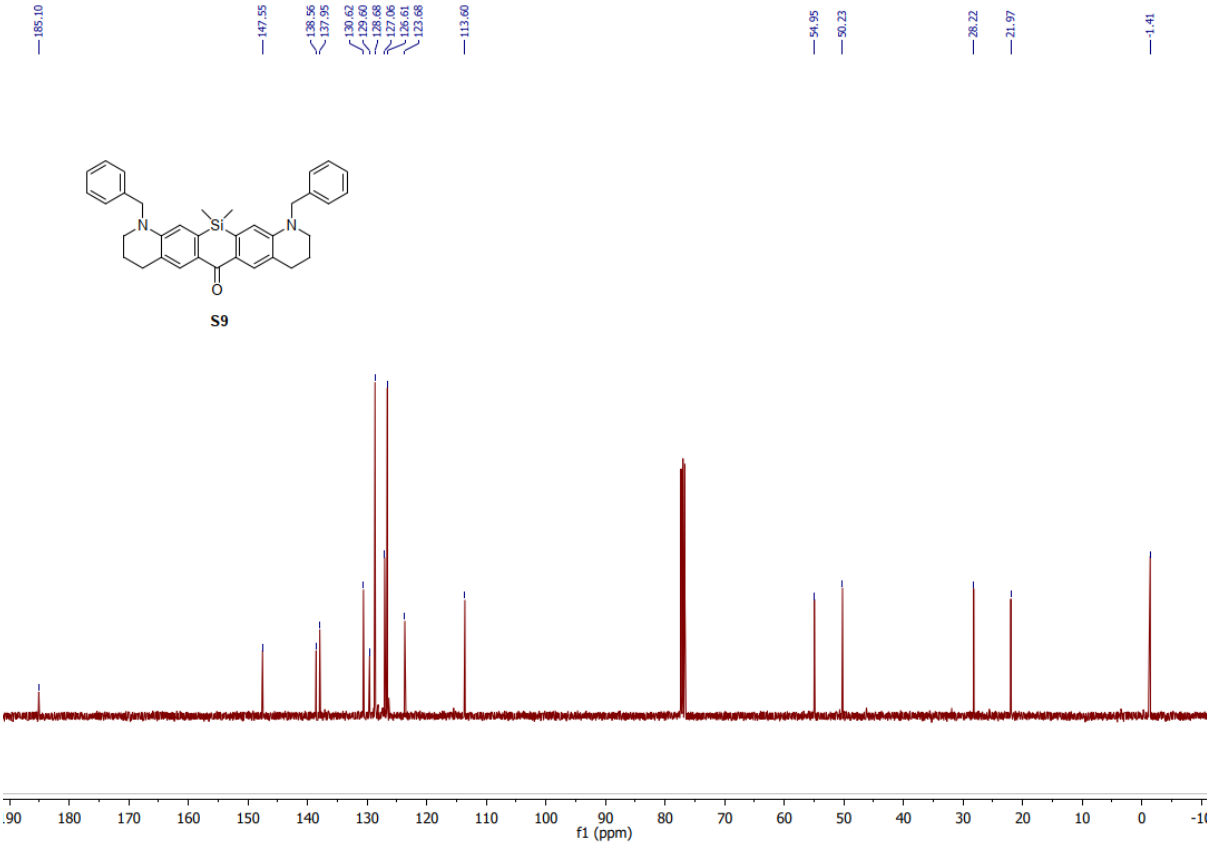

^{13}C NMR ($CDCl_3$, 101 MHz) of compound **S9**

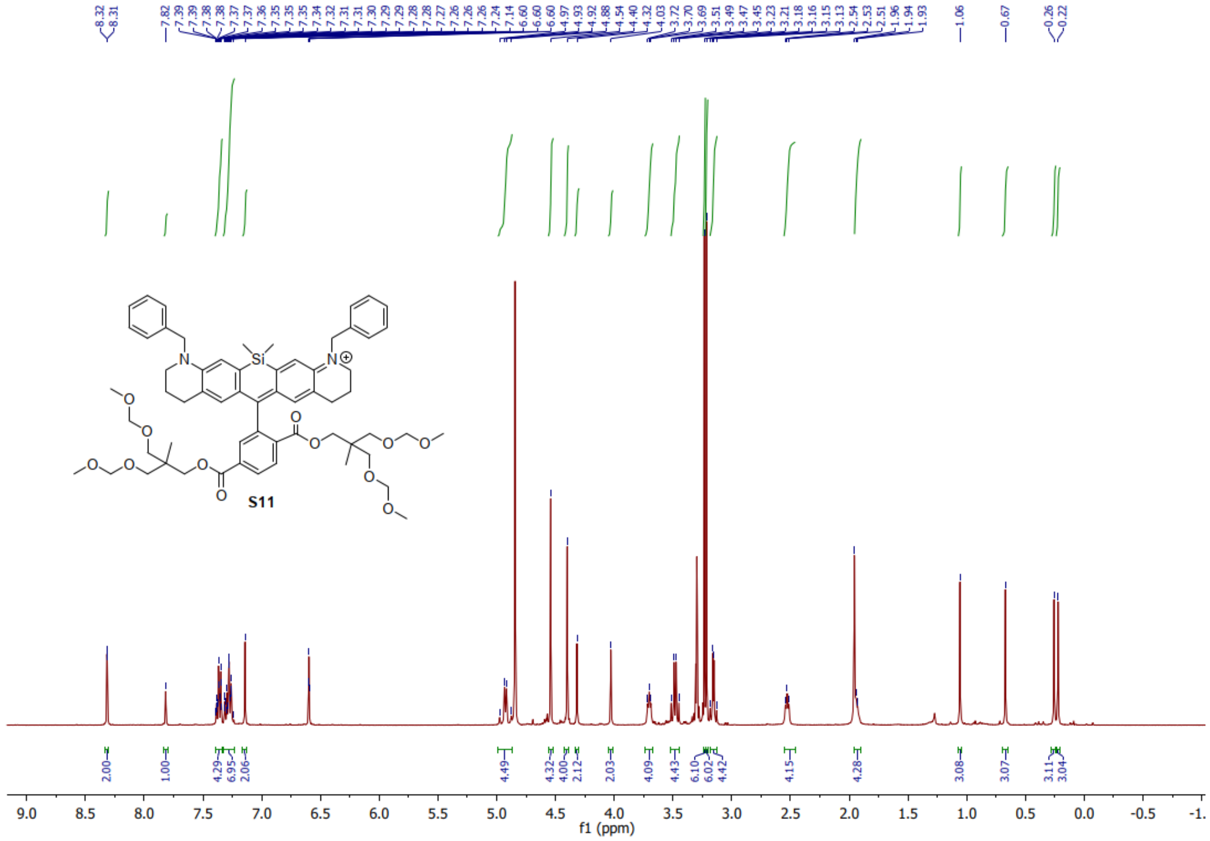

^{1}H NMR (CD_3OD, 400 MHz) of compound **S11**

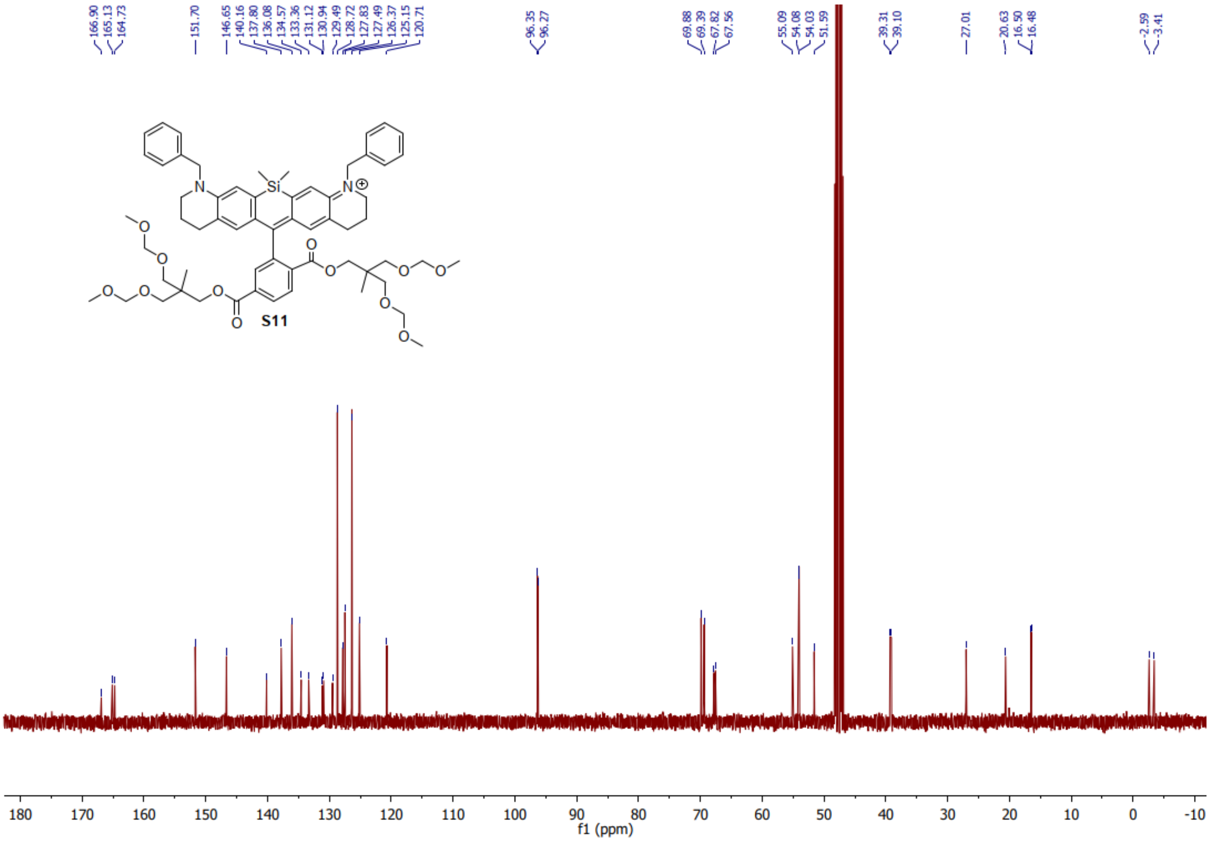

^{13}C NMR (CD_3OD, 101 MHz) of compound **S11**

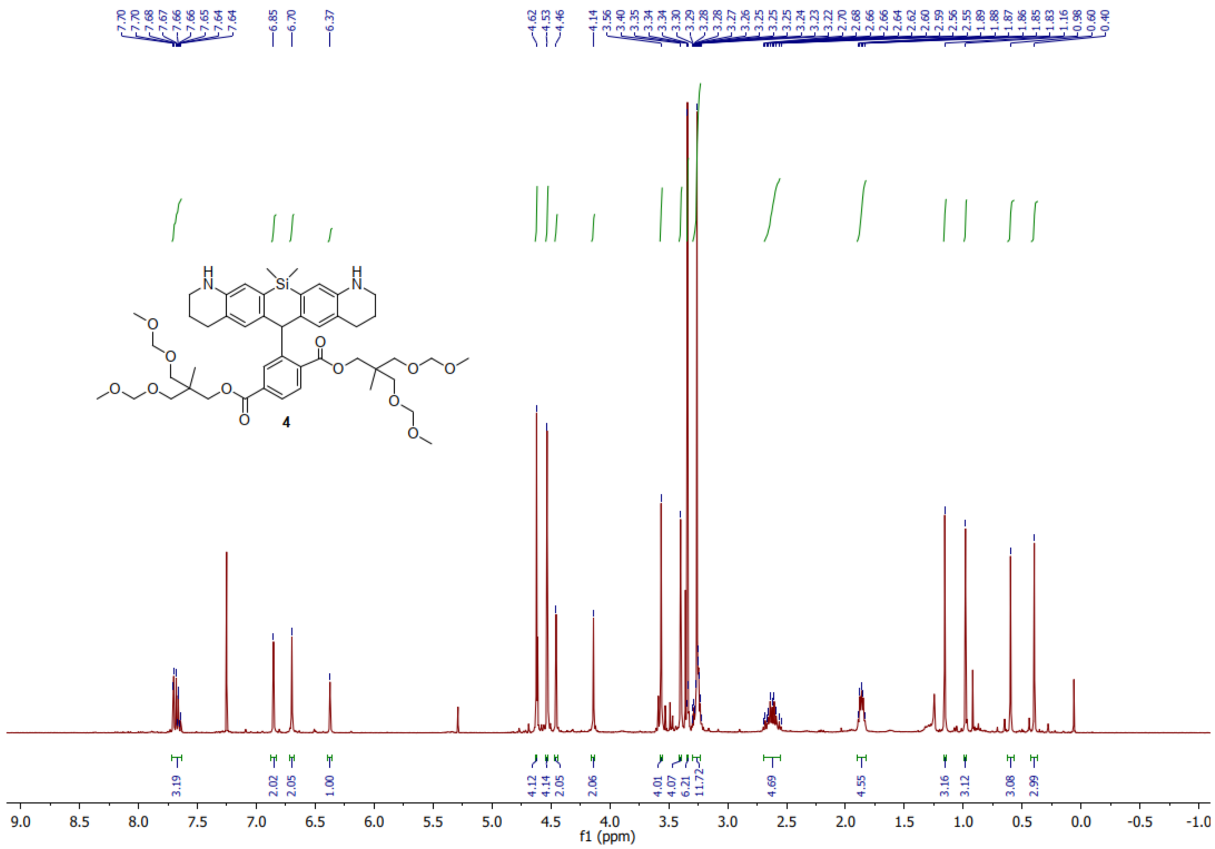

^{1}H NMR ($CDCl_3$, 400 MHz) of compound **4**

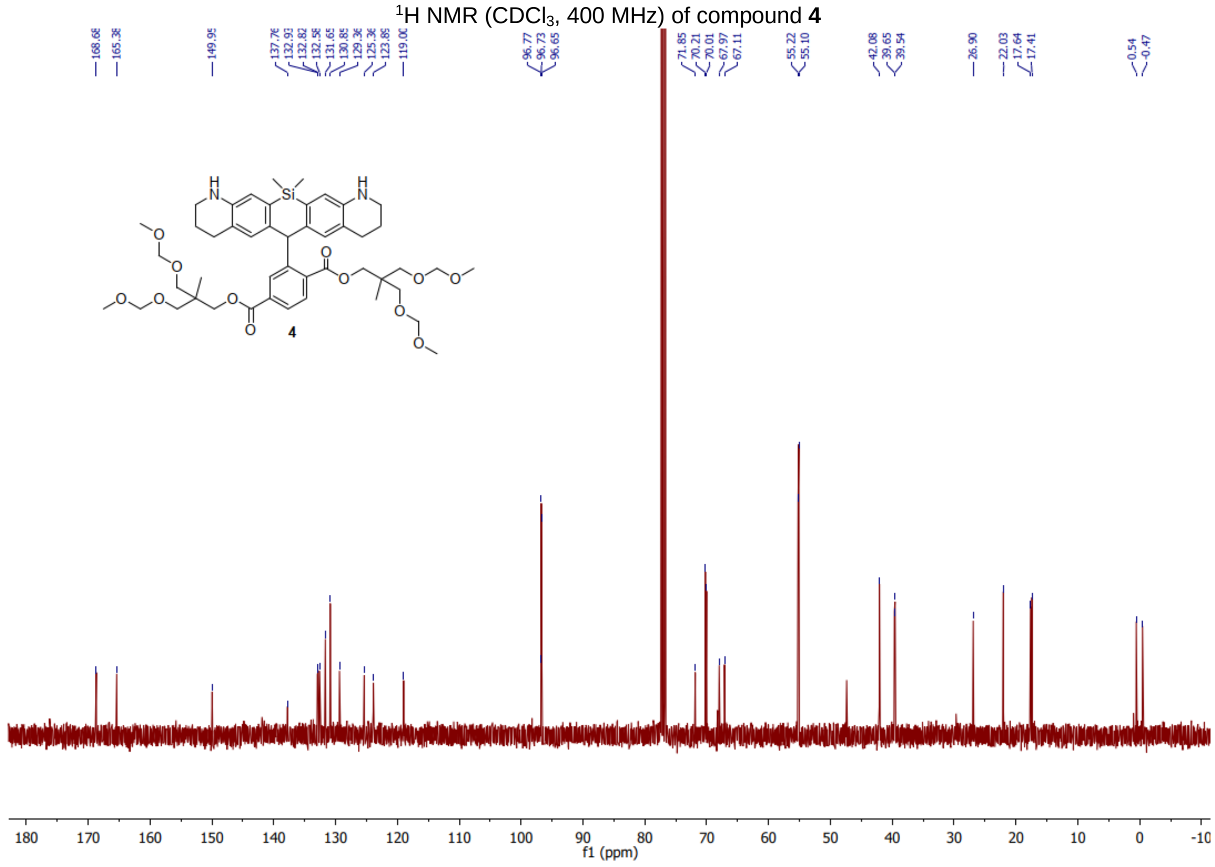

^{13}C NMR ($CDCl_3$, 101 MHz) of compound **4**

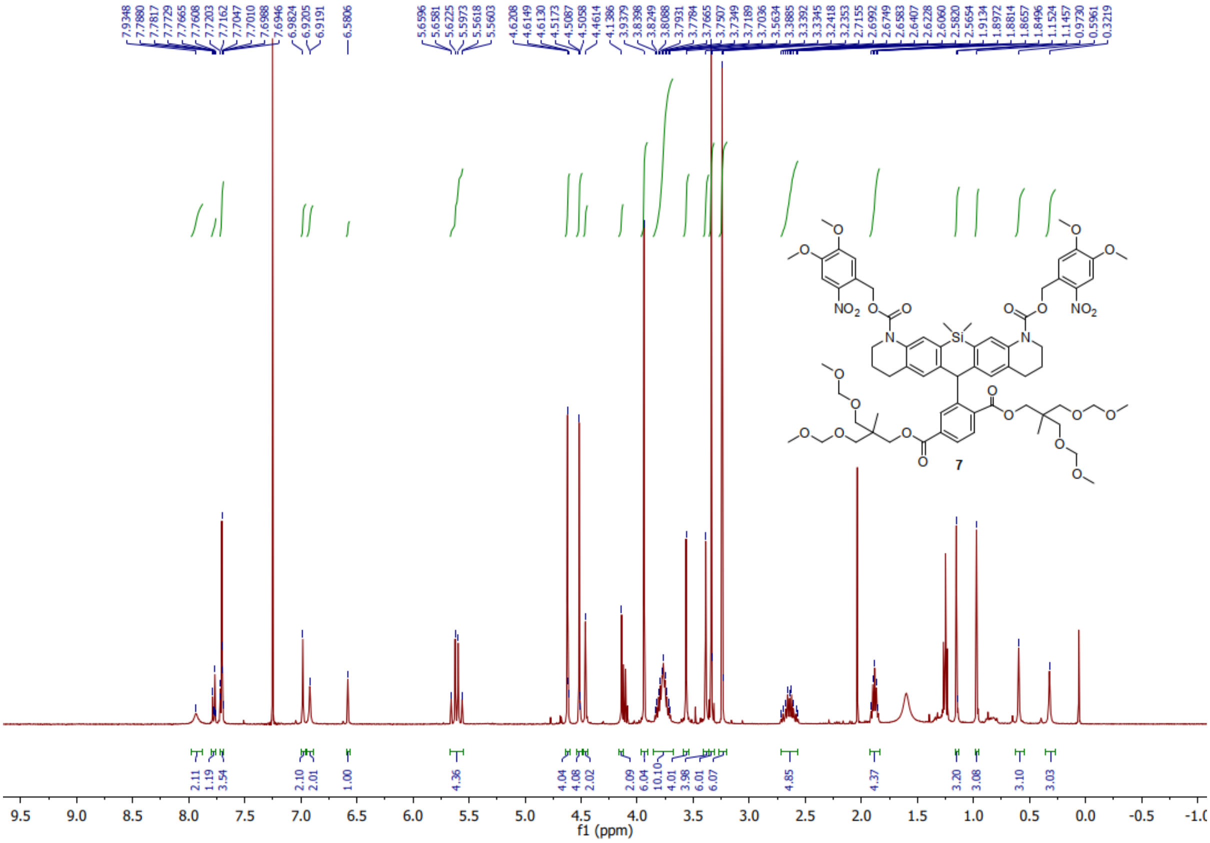

^{1}H NMR ($CDCl_3$, 400 MHz) of compound **7**

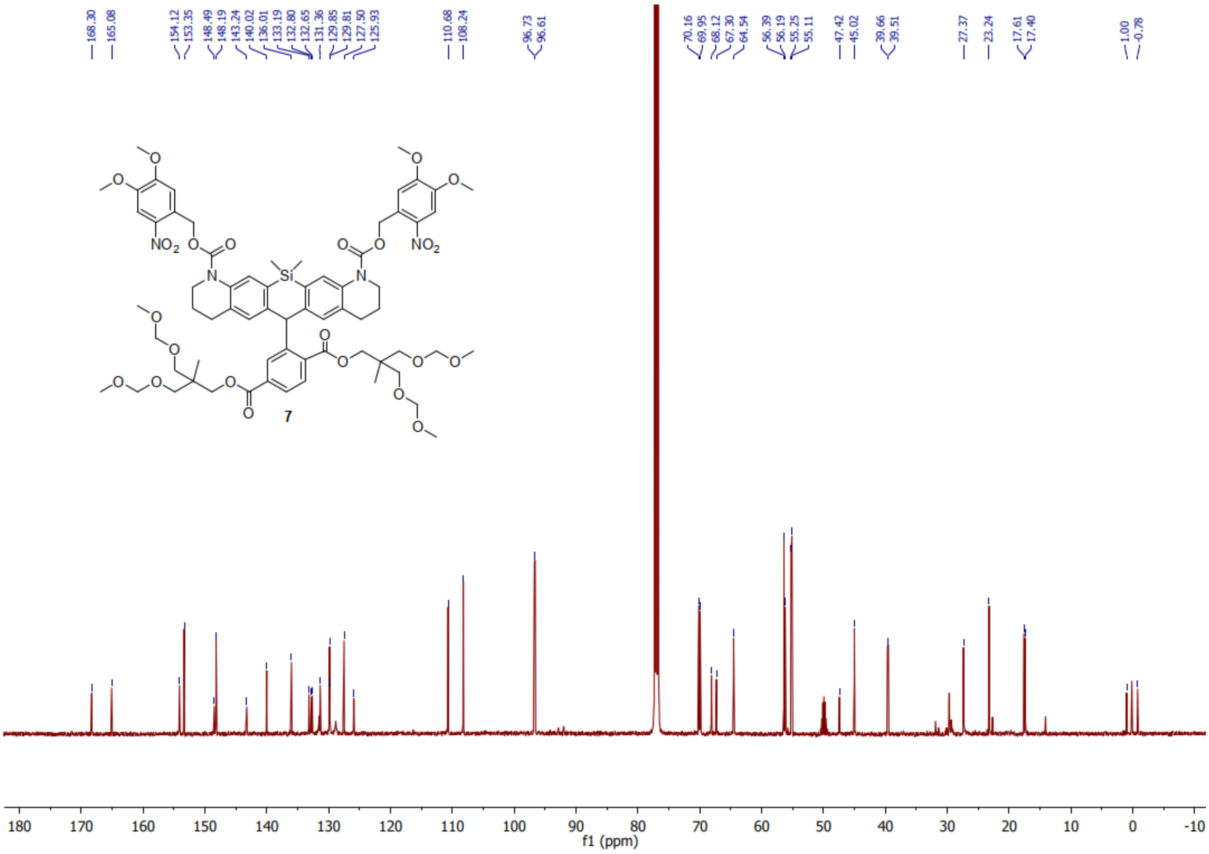

^{13}C NMR ($CDCl_3$, 126 MHz) of compound **7**

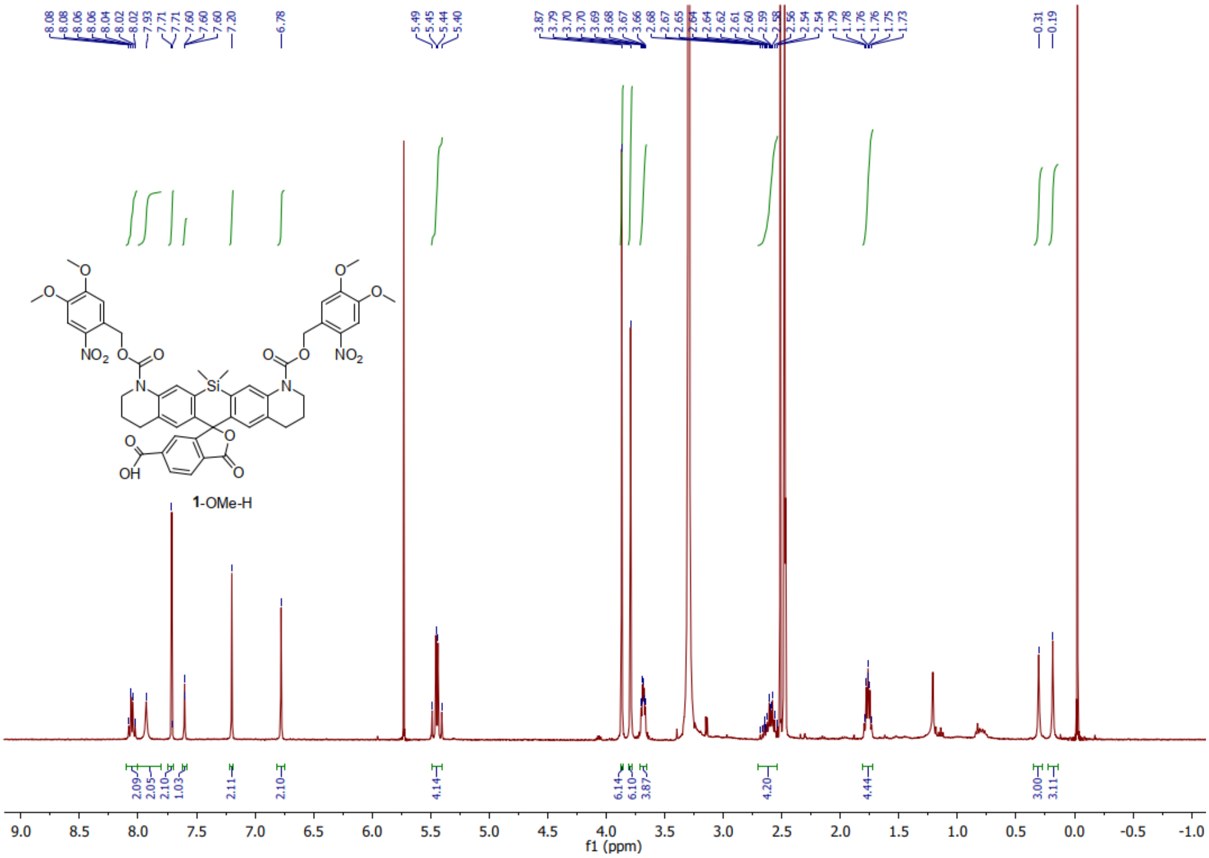

^{1}H NMR (DMSO-d_6, 400 MHz) of compound **1**-OMe-H

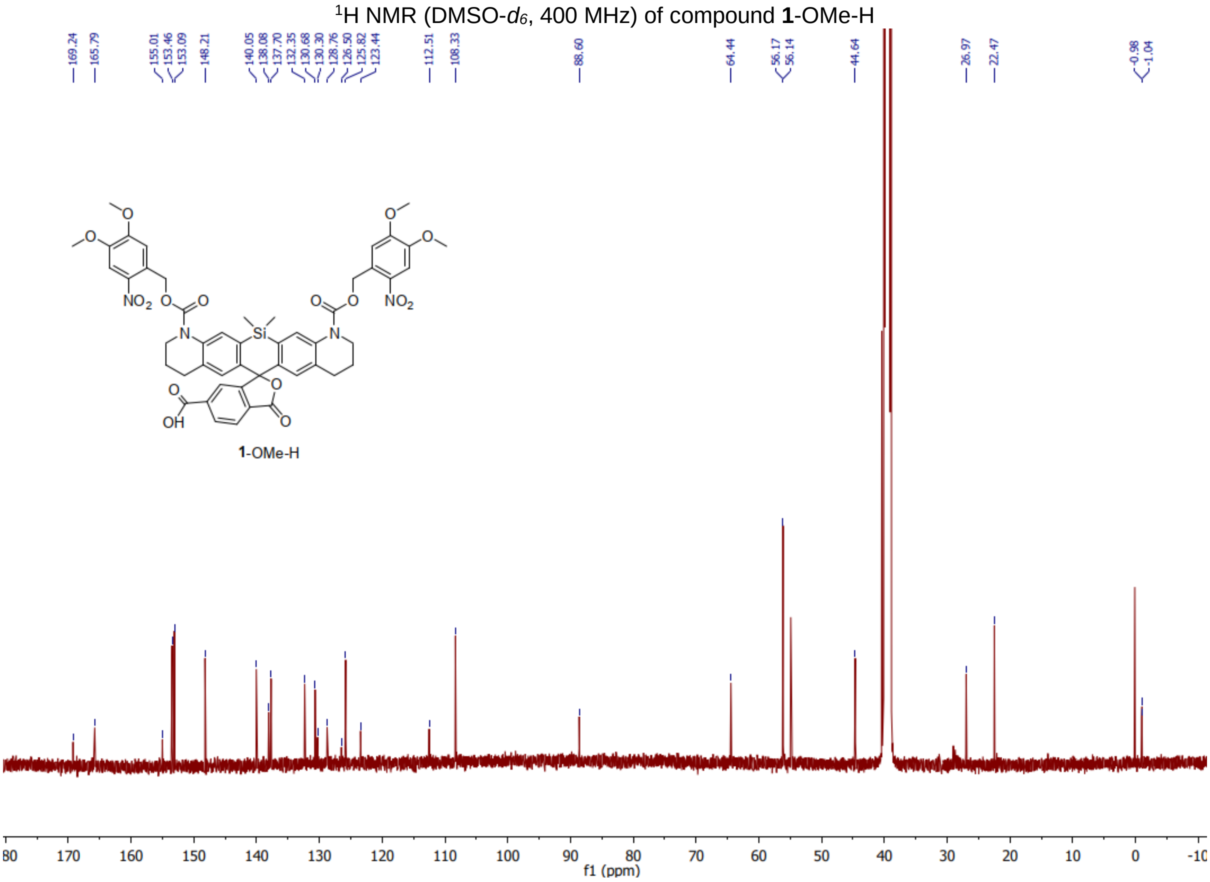

^{13}C NMR (DMSO-d_6, 126 MHz) of compound **1**-OMe-H

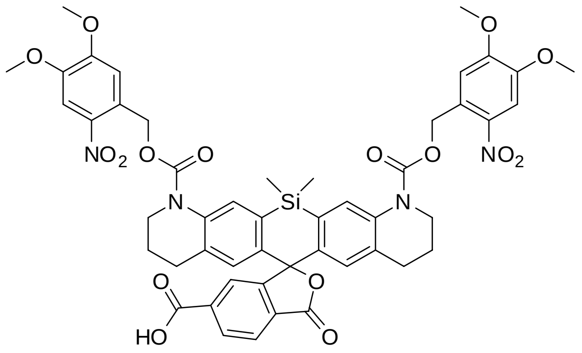

1-OMe-H

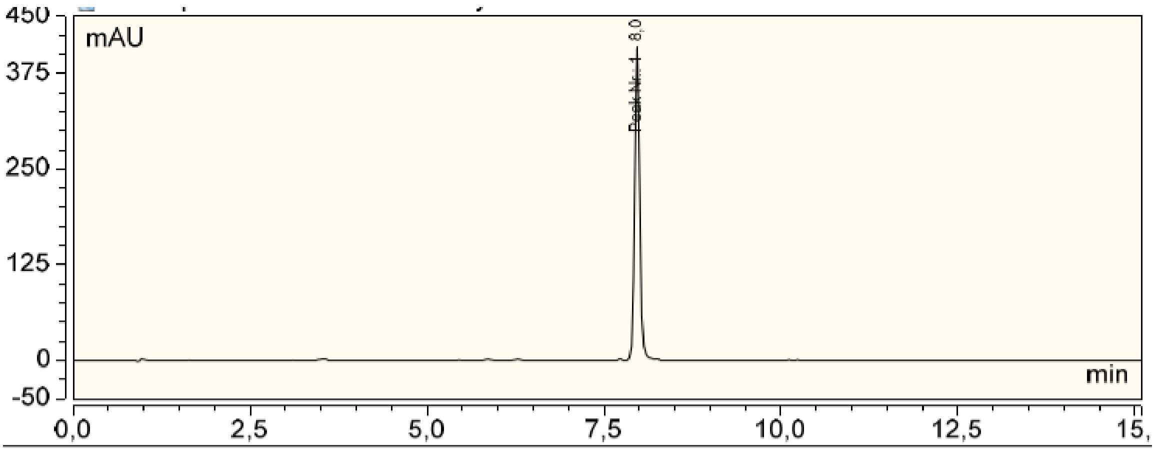

Analytical HPLC of compound **1**-OMe-H

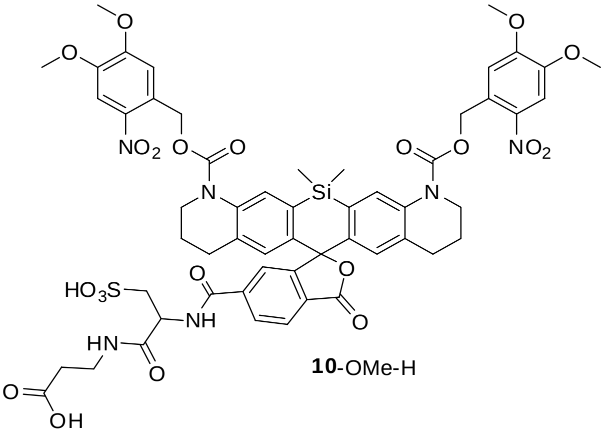

10-OMe-H

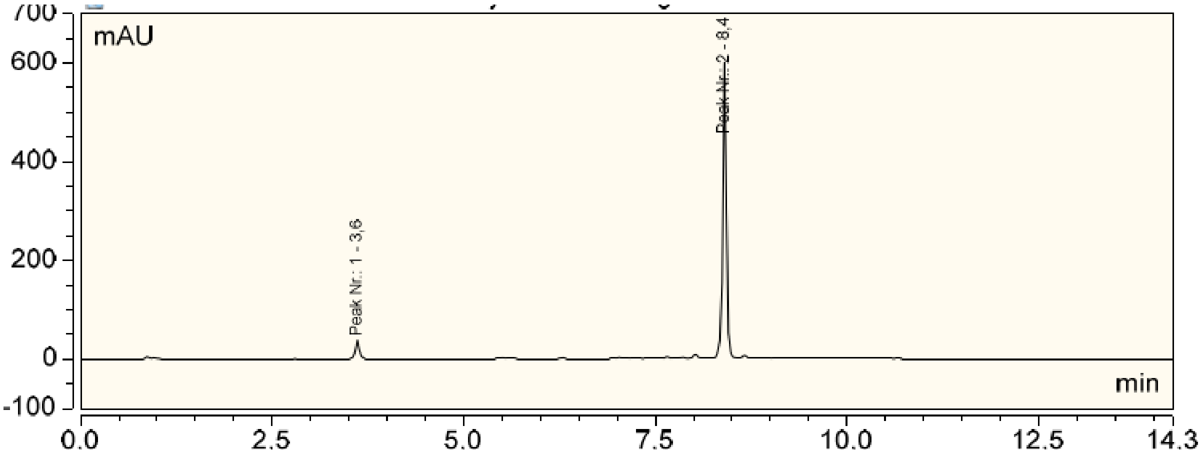

Analytical HPLC of compound **10**-OMe-H

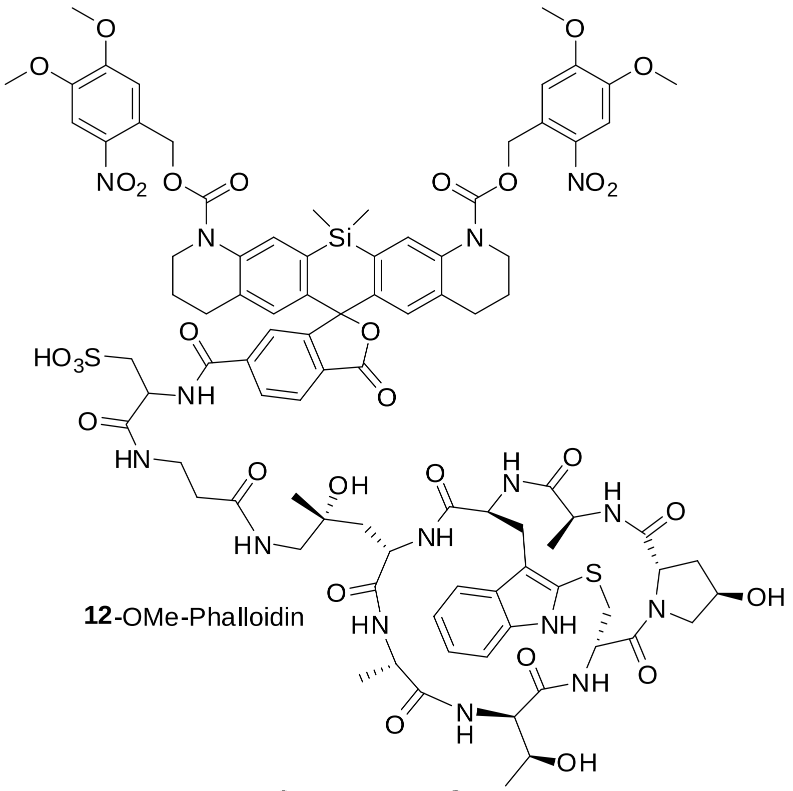

12-OMe-Phalloidin

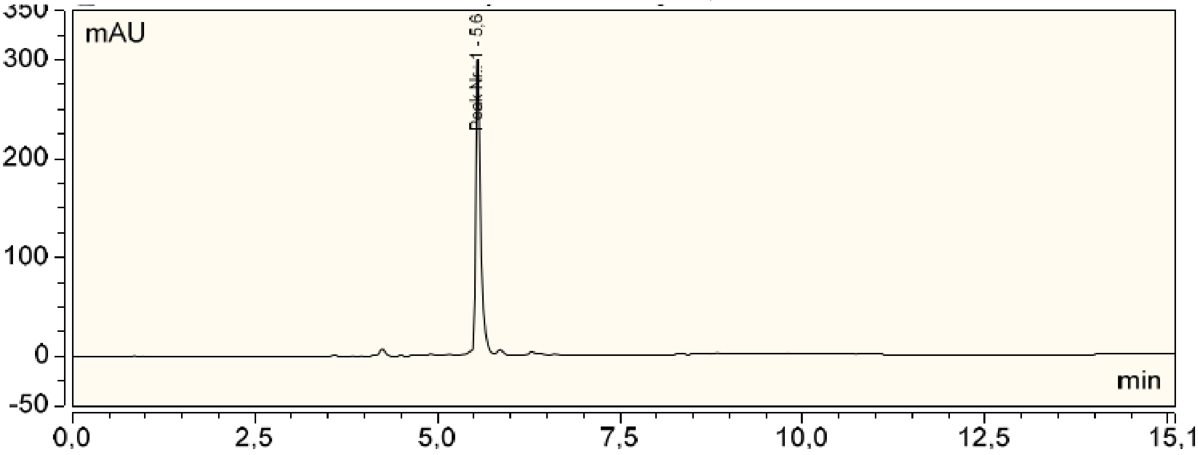

Analytical HPLC of compound **12**-OMe-Phalloidin

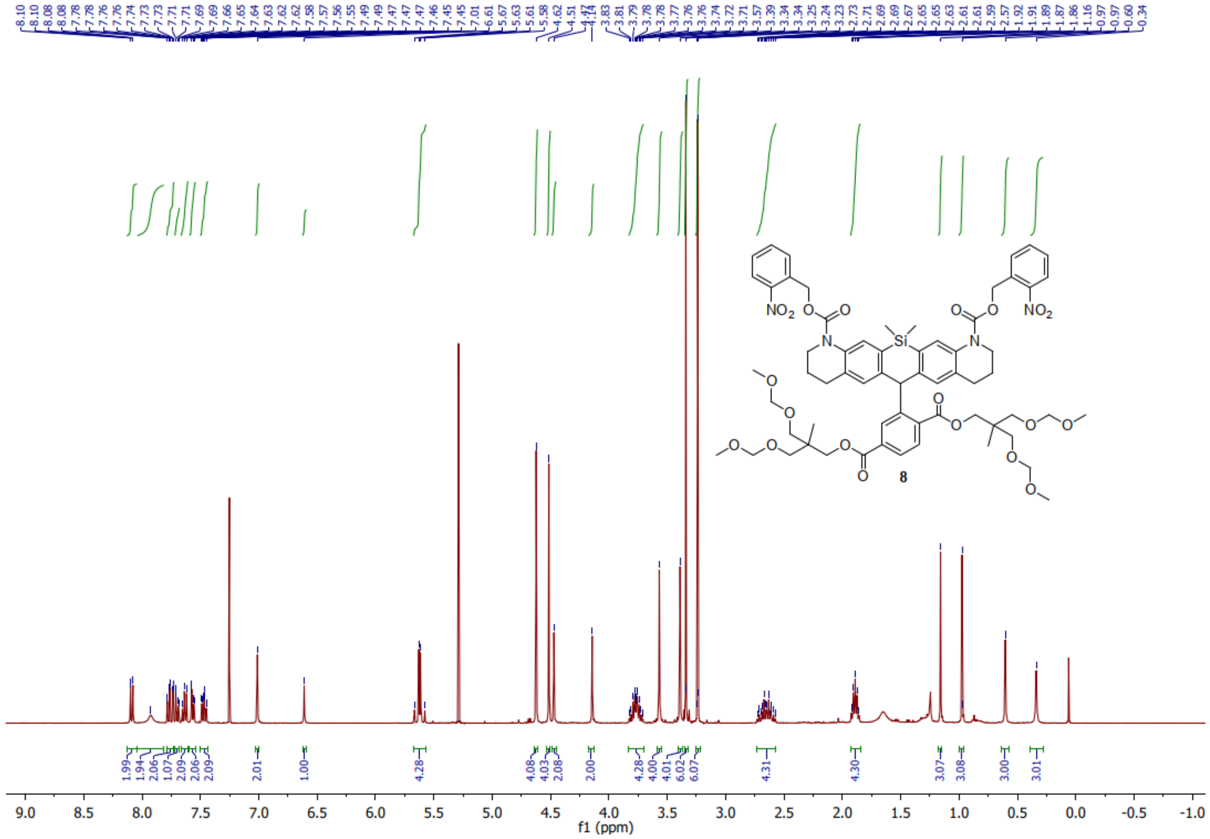

^{1}H NMR ($CDCl_3$, 400 MHz) of compound **8**

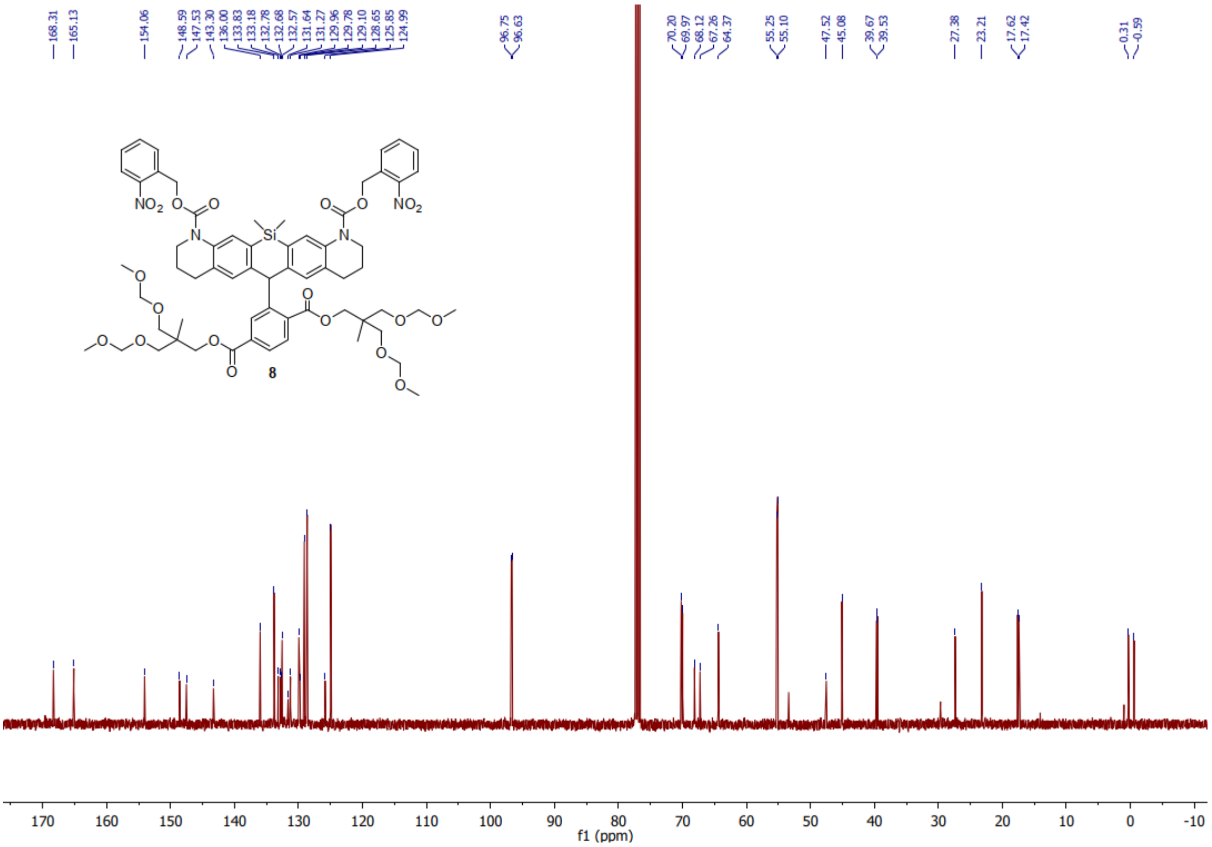

^{13}C NMR ($CDCl_3$, 101 MHz) of compound **8**

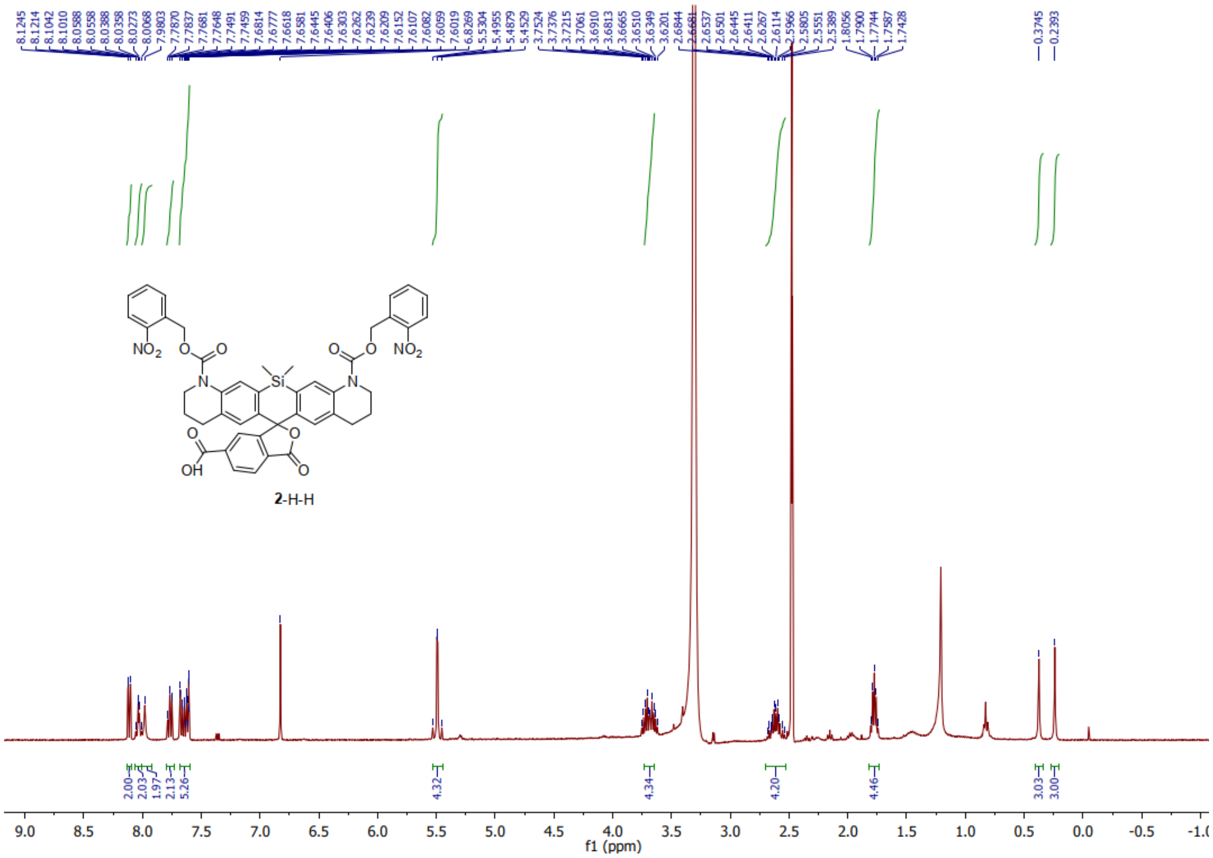

^{1}H NMR (DMSO-d_6, 400 MHz) of compound **2**-H-H

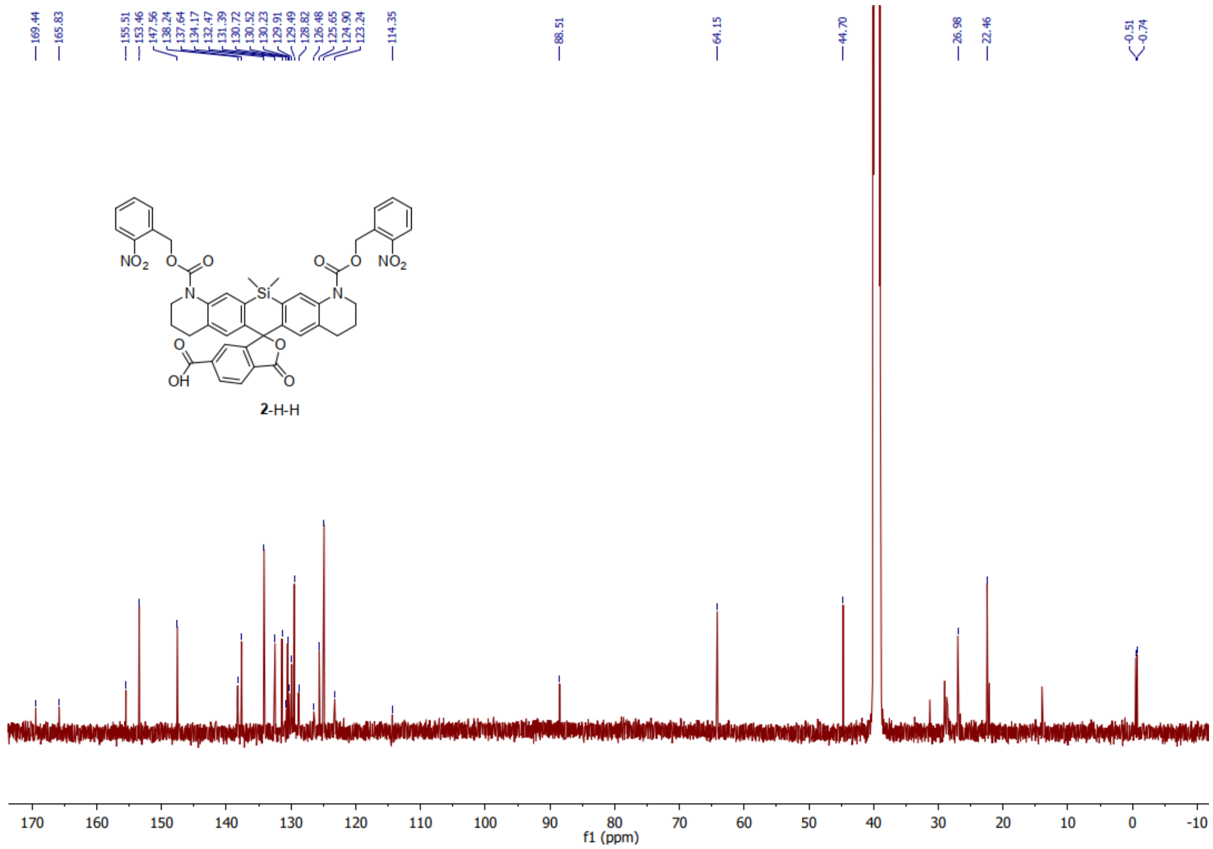

^{13}C NMR (DMSO-d_6, 101 MHz) of compound **2**-H-H

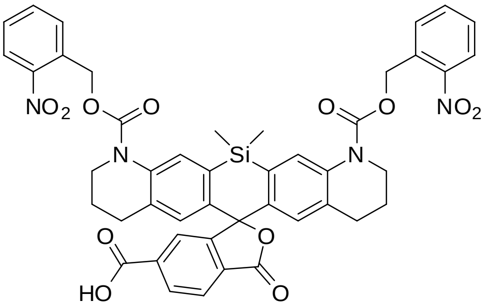

2-H-H

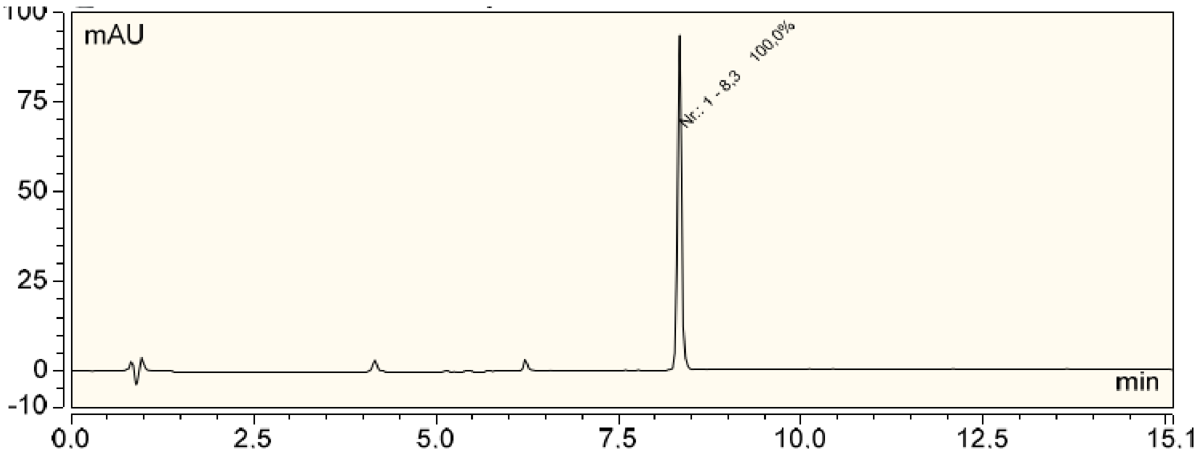

Analytical HPLC of compound **2**-H-H

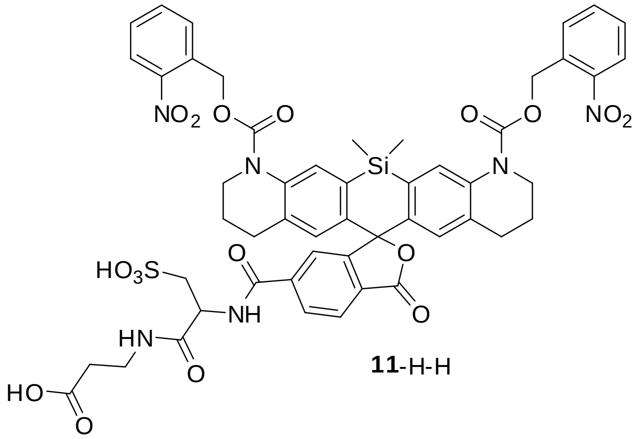

11-H-H

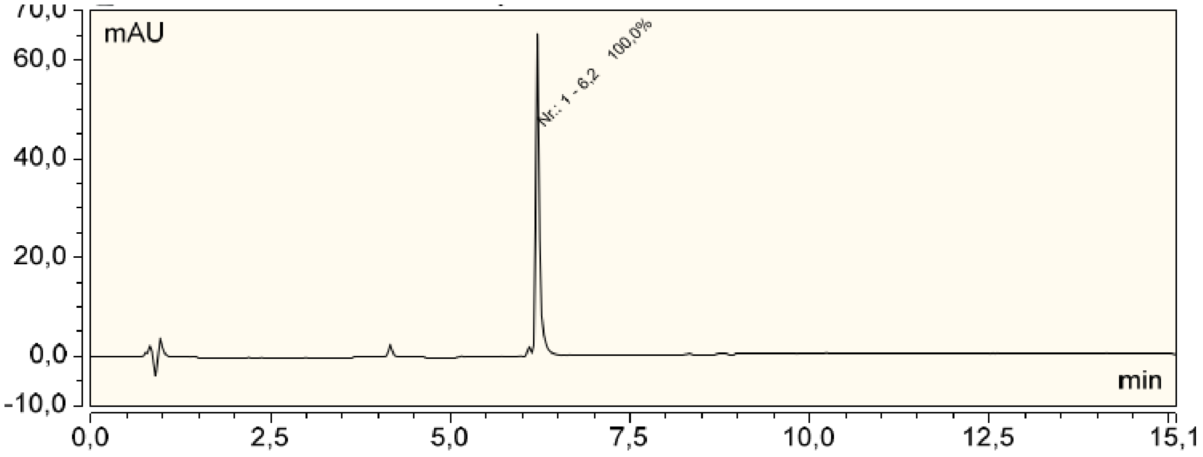

Analytical HPLC of compound **11**-H-H

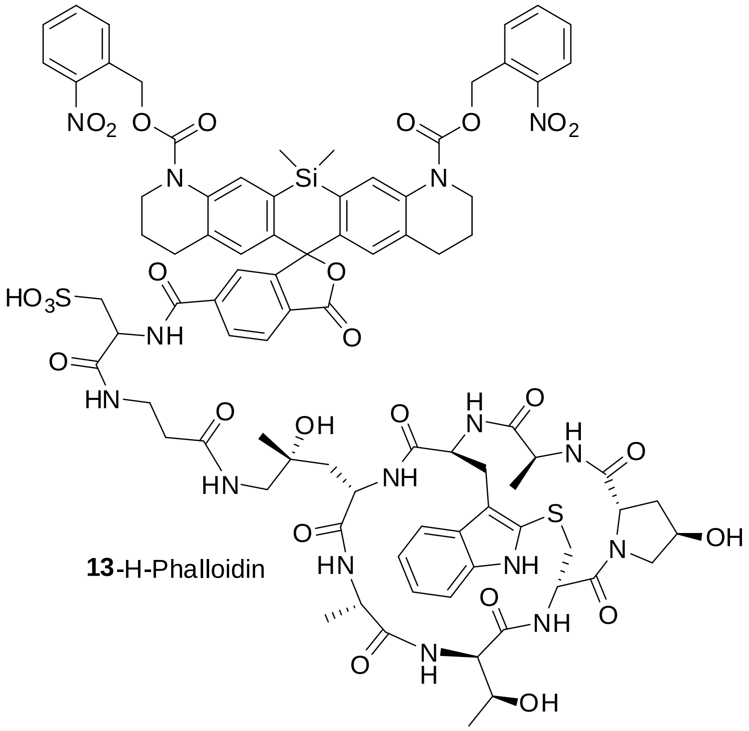

13-H-Phalloidin

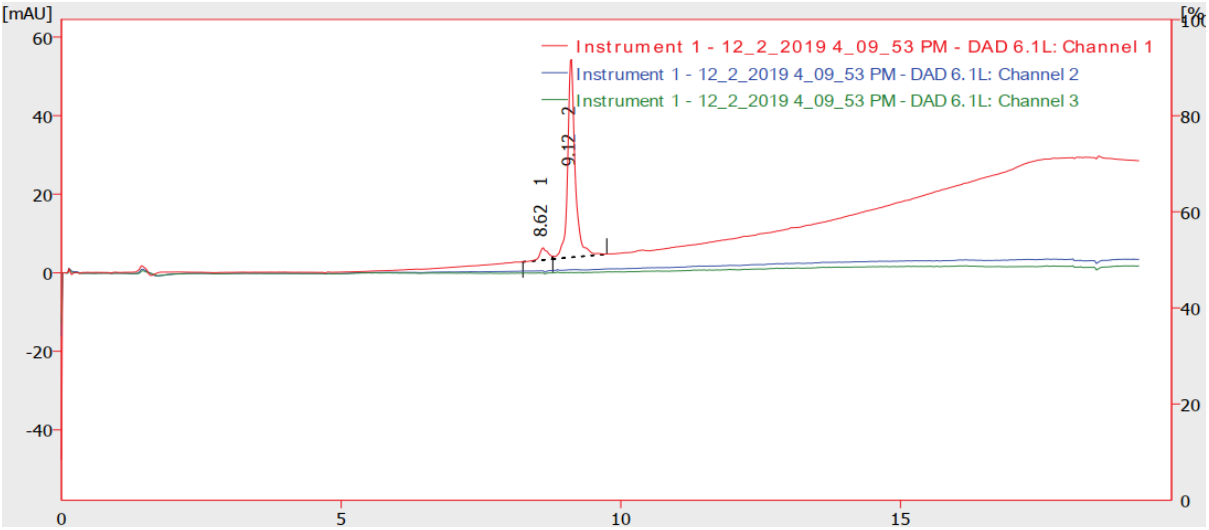

Analytical HPLC of compound **13**-H-Phalloidin

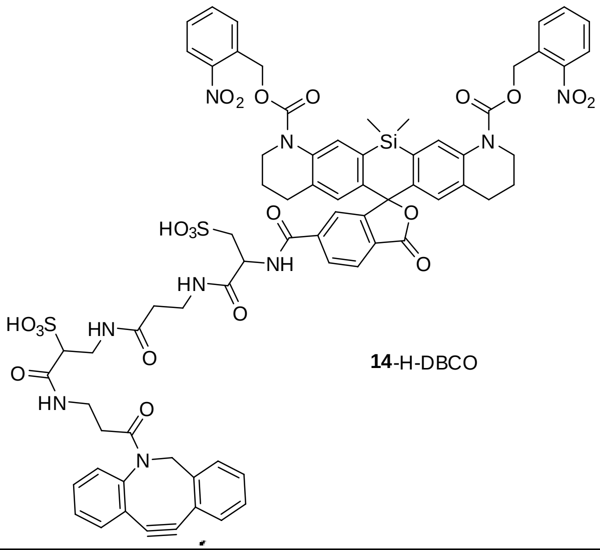

14-H-DBCO

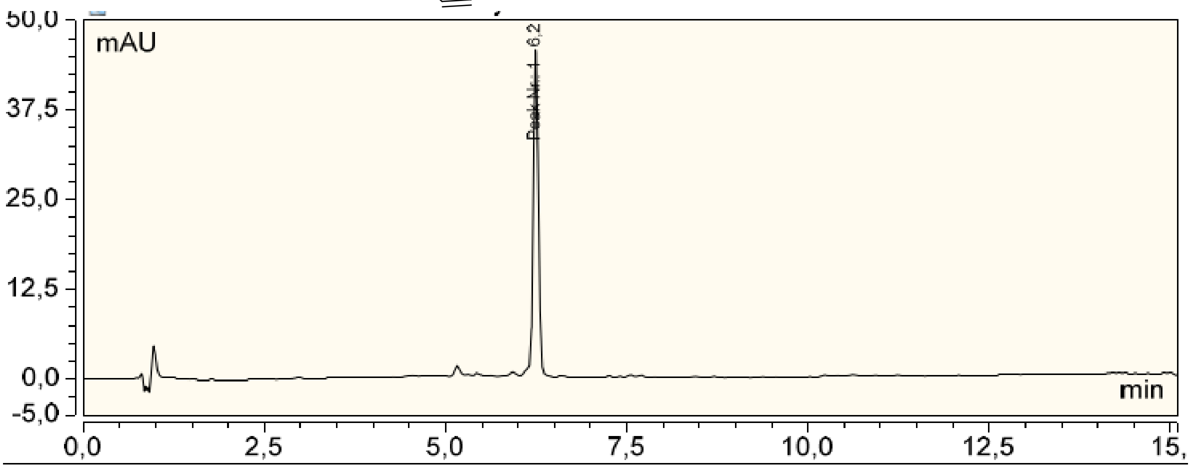

Analytical HPLC of compound **14**-H-DBCO

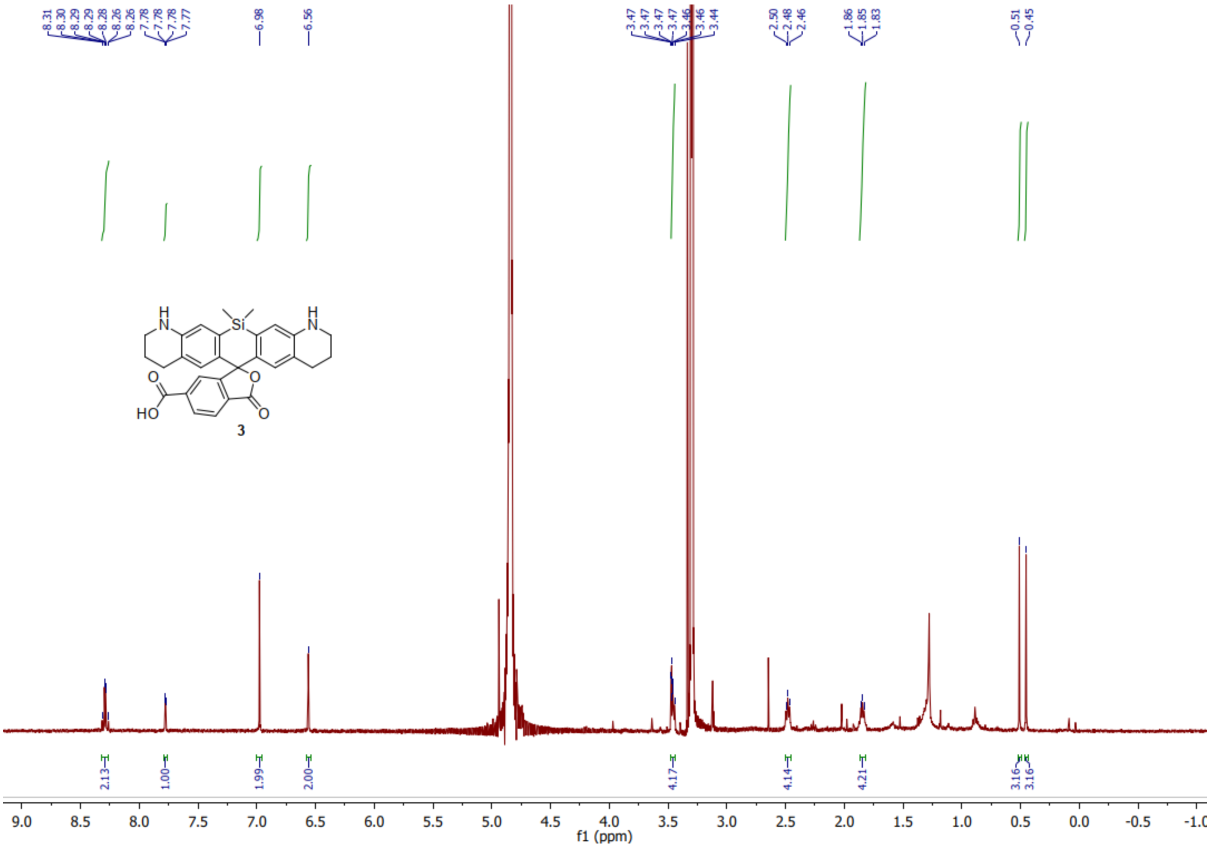

^{1}H NMR (CD_3OD, 400 MHz) of compound **3**

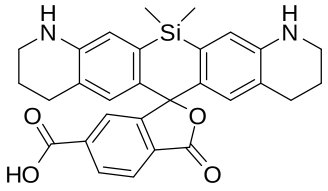

3

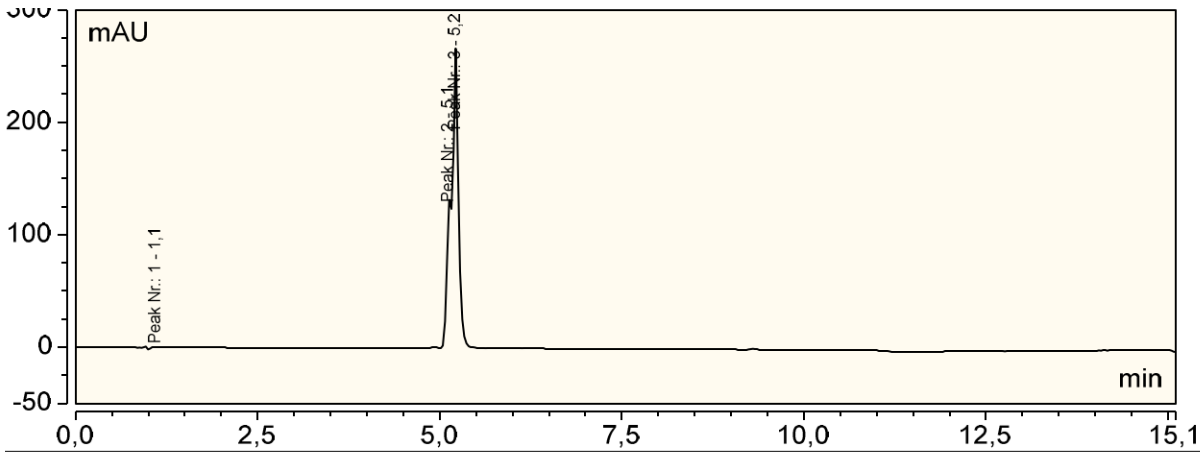

Analytical HPLC of compound **3**

References

[1] a) A. N. Butkevich, M. L. Bossi, G. Lukinavičius, S. W. Hell, *J. Am. Chem. Soc.* **2018**, *141*, 981-989; b) K. Uno, M. L. Bossi, T. Konen, V. N. Belov, M. Irie, S. W. Hell, *Adv. Opt. Mater.* **2019**, *7*.
[2] T. Pleiner, M. Bates, S. Trakhanov, C.-T. Lee, J. E. Schliep, H. Chug, M. Böhning, H. Stark, H. Urlaub, D. Görlich, *eLife* **2015**, *4*.
[3] a) A. N. Butkevich, G. Y. Mitronova, S. C. Sidenstein, J. L. Klocke, D. Kamin, D. N. H. Meineke, E. D'Este, P.-T. Kraemer, J. G. Danzl, V. N. Belov, S. W. Hell, *Angew. Chem. Int. Ed.* **2016**, *55*, 3290-3294; b) J. B. Grimm, T. A. Brown, A. N. Tkachuk, L. D. Lavis, *ACS Cent.l Sci.* **2017**, *3*, 975-985.
[4] J. B. Grimm, T. Klein, B. G. Kopek, G. Shtengel, H. F. Hess, M. Sauer, L. D. Lavis, *Angew. Chem. Int. Ed.* **2016**, *55*, 1723-1727.
[5] a) R. G. Dyer, K. D. Turnbull, *J. Org. Chem.* **1999**, *64*, 7988-7995; b) G. E. Negri, T. J. Deming, *ACS Macro Lett.* **2016**, *5*, 1253-1256.
[6] B. Roubinet, M. Bischoff, S. Nizamov, S. Yan, C. Geisler, S. Stoldt, G. Y. Mitronova, V. N. Belov, M. L. Bossi, S. W. Hell, *J. Org. Chem.* **2018**, *83*, 6466-6476.
[7] A. M. Brouwer, *Pure Appl. Chem.* **2011**, *83*, 2213-2228.
